# Sigmatropic rearrangements of ynamides in stereoselective synthesis of complex molecular architectures: a comprehensive review

**DOI:** 10.1039/d6ra06430b

**Published:** 2026-09-11

**Authors:** Archana Mishra, Subhransu Sekhar Pati, Jaya Prakash Das

**Affiliations:** a Organic Synthesis and Catalysis Laboratory, Department of Chemistry, Ravenshaw University Cuttack-753003 India jpdas@ravenshawuniversity.ac.in

## Abstract

Transition metal- and Lewis acid-catalysed stereoselective sigmatropic rearrangements of organic molecular scaffolds bearing ynamides or their derivatives have achieved substantial progress during the last two decades, showing contemporary interest in the exploration of ynamide chemistry. The reactivity and chemical characteristics of ynamides are mostly determined by the triple bond's intrinsic interaction with the adjacent electron-donating nitrogen atom. The typical polarity of C–N functionality coincides with a diverse array of synthetic possibilities that go well beyond conventional alkyne chemistry. Due to their electronic polarity, ynamides generate a keteniminium intermediate *in situ* in various catalytic systems, which can be employed in reactions that require high levels of regio- and stereoselectivity toward the development of strategies for the synthesis of structurally versatile N-containing complex molecular frameworks. These strategies reflect the constant striving and creativity in the design and execution of new synthetic methods utilizing ynamides. The present review summarises the current state of the art for the design of stereoselective sigmatropic rearrangements of ynamides and their derivatives, leading to the formation of natural product precursors and molecules bearing quaternary or tertiary stereocentres in both cyclic and acyclic frameworks. The organization of this review relies on the synthetic strategies developed to date and is divided into sections depending on the type of sigmatropic rearrangements. Due to the ready availability of a wide range of ynamides obtained from straightforward synthetic methods and commercial sources, it can be predicted that this field of chemistry will continue to grow in the coming years, providing an excellent opportunity for further enrichment of mainstream synthetic strategies.

## Abstract 2

In this review, stereoselective sigmatropic rearrangements of ynamides, leading to the formation of natural product precursors and molecules bearing quaternary or tertiary stereocentres in both cyclic and acyclic frameworks, have been highlighted.

## Graphical abstract

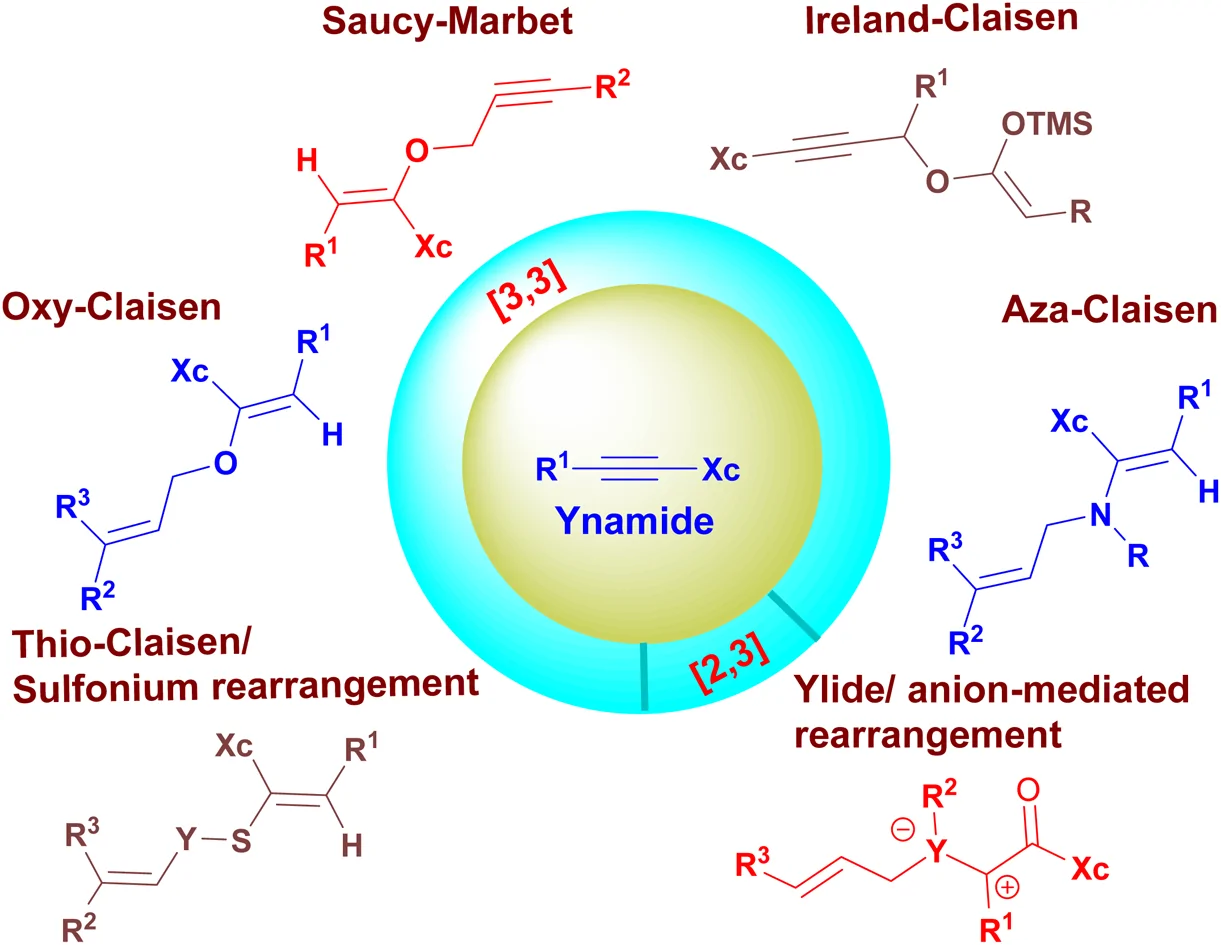

## 1 Introduction

Organic synthesis offers competitive techniques for creating a realm of entirely new synthetic frameworks and natural products and has emerged as one of the most powerful driving forces behind sustainable development and healthcare. It is well established that organic synthesis has been highly dependent upon molecular rearrangements to furnish complex natural products over the last few decades.^1^ Typically, rearrangement reactions involve both bond breaking and bond formation, resulting in changes in connectivity and, in some cases, metamorphosis of the original structures.^1*d*^ Furthermore, stereoselective molecular rearrangements such as pericyclic reactions,^2*a*^ photochemical rearrangements,^2*a*^ Schmidt rearrangement,^2*b*^ Hofmann rearrangement,^2*b*^ and Curtius rearrangement^2*b*^ play a pivotal role in the field of organic chemistry and pharmaceuticals. Among these categories of molecular rearrangements, sigmatropic rearrangements are a unique subset of pericyclic reactions that provide a catalyzed, direct rearrangement pathway for constructing various heterocycles and sophisticated molecular fragments with high levels of selectivity.^3^

**Fig. 1:**
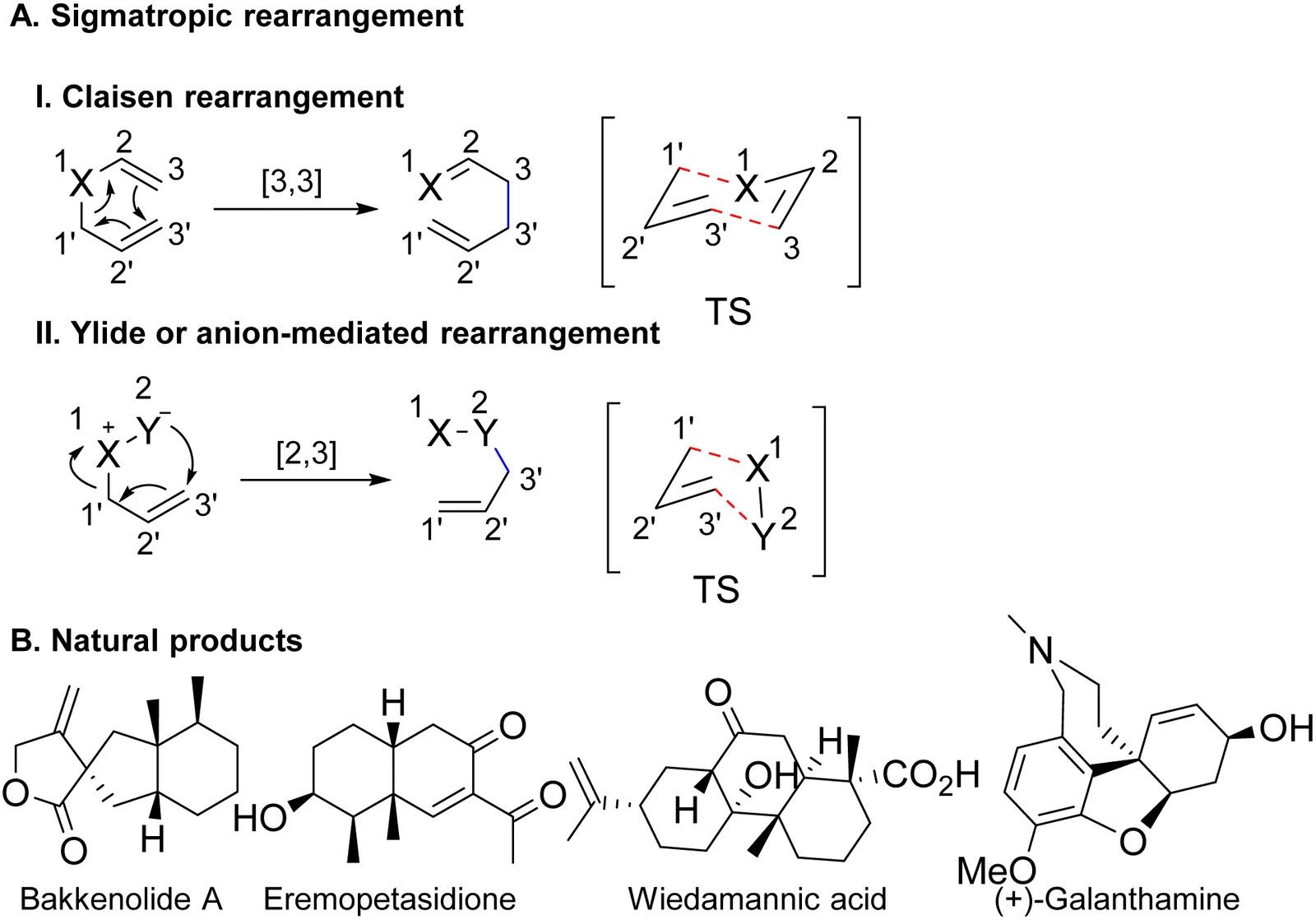
(A) Different types of Sigmatropic rearrangements (B) natural products.

The field of sigmatropic rearrangements is fairly extensive, and a substantial body of information exists on this topic, which offers clear synthetic advantages. Sigmatropic rearrangement reactions are defined as reactions involving the migration of a sigma bond across a conjugated pi system to a different position within the same molecule, without altering the overall count of sigma bonds and pi bonds (Fig. 1A).^1*b*^

These rearrangements play a pivotal role in constructing new carbon–carbon bonds, generating sterically congested centres, and regulating stereochemistry in the synthesis of macromolecular frameworks with diverse biological activities. The structurally diverse and complex natural products, such as polyketides, terpenes, and alkaloids, can be assembled through sigmatropic rearrangements to form the respective carbon skeletons (Fig. 1B).^3*b*–*d*^

The studies addressing [3,3]- and [2,3]-sigmatropic rearrangements involved in different reactions were the most prevalent; [1,3]-sigmatropic rearrangements were less common, and [1,2], [1,5], and [3,5]-sigmatropic rearrangements were highlighted in relatively few publications.^3*e*^ L. Claisen introduced the initial sigmatropic rearrangement in 1912, wherein allyl vinyl ether undergoes a thermal [3,3]-sigmatropic rearrangement to produce γ,δ-unsaturated carbonyl compounds (Fig. 1A and I).^4^ As one of the convenient processes for the generation of carbon–carbon bonds, many analogous reactions have been established to accomplish enhanced reactivity as well as for the synthesis of natural products and pharmaceuticals. In addition, the well-defined and predictable transition state of catalytic sigmatropic rearrangements provides a high degree of diastereoselectivity and enantioselectivity, enabling the construction of numerous chiral building blocks.^5^ The range of substrates has been extended from oxygen-containing molecules to nitrogen-, selenium- and sulphur-containing precursors, whereas alkenes, alkynes, and allenes have also been employed for various transformations.^6^ Alkynes with an allyl ether moiety served as one of the direct and efficient sigmatropic rearrangement precursors to produce functional backbones in an atom-economic and readily accessible manner.^7^ Despite the outstanding achievements in the simple alkyne system, achieving enhanced selectivity is a key issue and remains desirable. As a result, several fundamental studies on this transformation are incessantly being developed using activated unsymmetrical alkynes to introduce improved selectivity.^7^

**Fig. 2:**
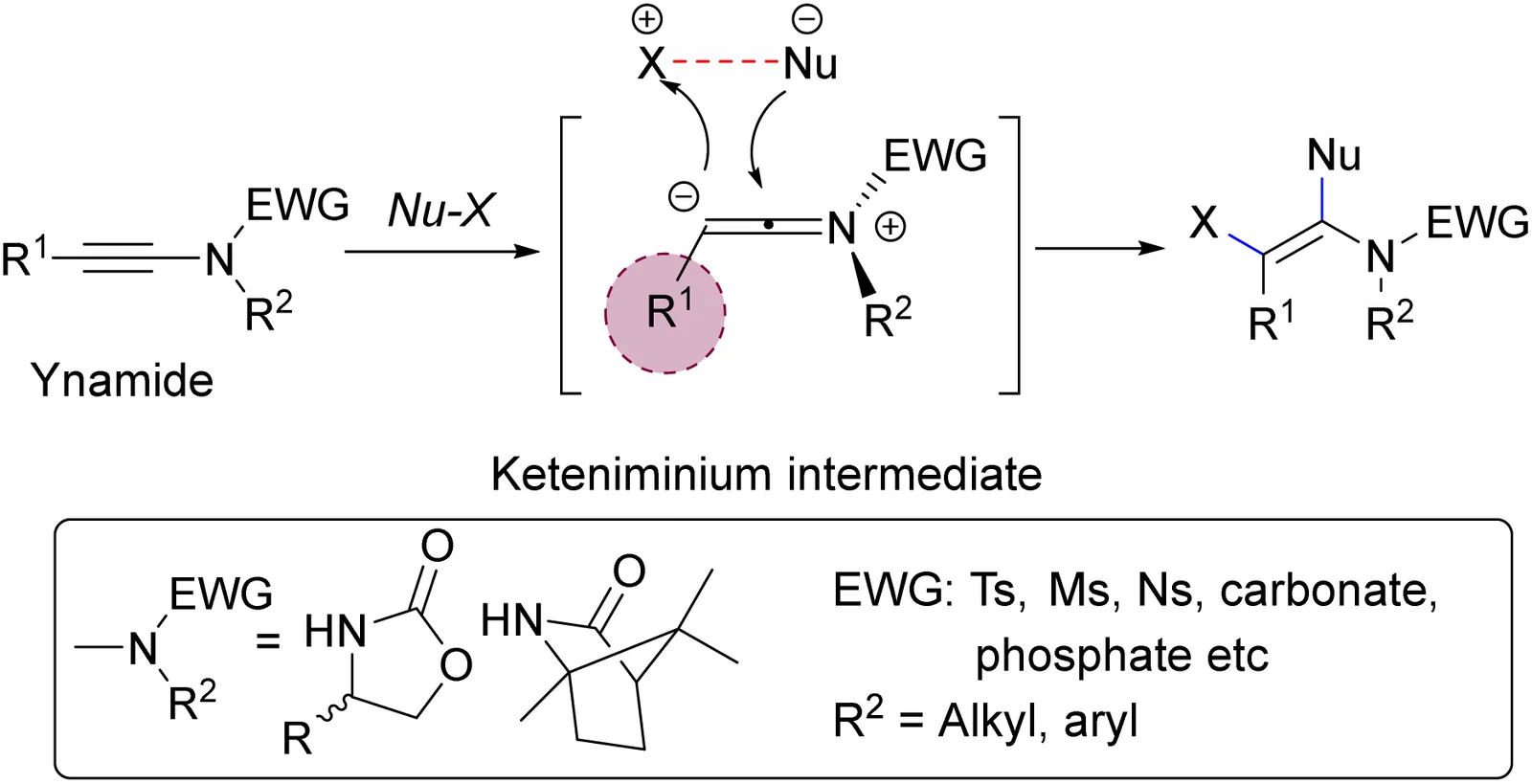
Nucleophilic addition reactions of ynamides.

Ynamides are a specific class of alkynes attached to a nitrogen atom, which bears an electron-withdrawing group (EWG) such as sulphonyl, alkoxycarbonyl, and acyl (Fig. 2).

They are not only commercially available and easily synthesised using straightforward methods but also effectively converted into a variety of C–C, C–N, C–O, and C–X bonds. The advancement and application of ynamide chemistry in the field of cycloadditions, cross-coupling reactions, ring-closing metathesis, and other tandem processes have garnered more attention over the past 20 years.^8^

The EWG or PG (protecting group) provides stability to the molecule under thermal and hydrolytic conditions. The reactivity and chemical characteristics of ynamides are mostly determined by the triple bond's interaction with the adjacent electron-donating nitrogen atom.^8*e*,*f*^ The typical polarity of C–N functionality coincides with a diverse array of synthetic possibilities that go well beyond conventional alkyne chemistry.^8*e*,*f*^ Due to their electronic polarity, ynamides generate a keteniminium intermediate *in situ* in various catalytic systems, which can be employed in reactions that require high levels of regio- and stereoselectivity (Fig. 2).^9^ This activation mechanism allows for the predictable addition of various types of nucleophiles (O, N, C, *etc.*) to the α-position of the nitrogen atom (Fig. 2).^8*h*^ Therefore, ynamides are proven to be more practical to work with and offer better reaction control in the synthesis of structurally versatile N-containing molecules.

**Fig. 3:**
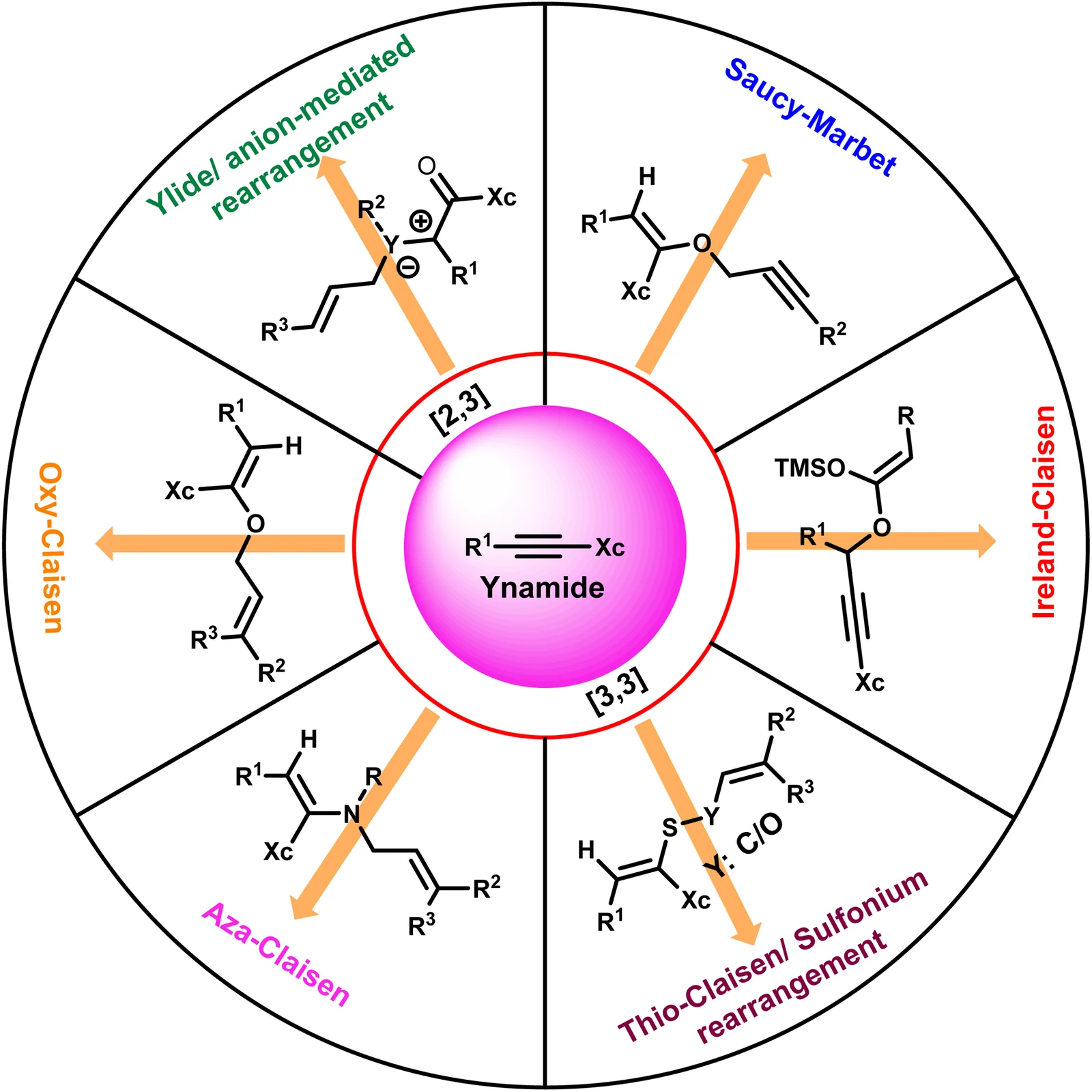
Current state of the art in the stereoselective sigmatropic rearrangement of ynamides.

However, owing to the structural importance of ynamides in organic synthesis, many sigmatropic rearrangement transformations using ynamides have been made possible by synthetic chemists in the last few decades. This offers an atom-economic pathway for the synthesis of allenamides and other functionalized organic molecules and for the construction of quaternary stereocentres in both cyclic and acyclic systems. In addition, the chiral ynamide substrate induces greater diastereoselectivity as well as excellent enantioselectivity during the sigmatropic rearrangement process. Nevertheless, a thorough conceptual summary of the developments in the stereoselective sigmatropic rearrangement processes of ynamides is lacking. With this prologue, we would like to summarise a series of interesting transformations *via* sigmatropic rearrangements and their conceptual inception, with ynamides serving as the key substrates. In this review, the current state of the art in the stereoselective sigmatropic rearrangement of ynamides is discussed, leading to the formation of natural product precursors, quaternary or tertiary stereocentres in both cyclic and acyclic systems. The discussion is organised by general reaction types, as illustrated in Fig. 3.

## 2 Current state of the art in the sigmatropic rearrangement of ynamides

Over the past few decades, various synthetic methods have been developed for the sigmatropic rearrangement of ynamides, exhibiting high regio- and stereoselectivity (Fig. 3).

Herein, we discuss the conceptual development of various synthetic approaches made towards the sigmatropic rearrangements of ynamides. Based on a thorough literature survey, we found that ynamides are mostly involved in [3,3]- and [2,3]-sigmatropic rearrangements (Fig. 3). The [2,3]-sigmatropic rearrangement reaction involves the ylide- or anion-mediated rearrangement for the formation of new complex molecular fragments. On the other hand, the [3,3]-sigmatropic rearrangement process is divided into five important subcategories, such as the formation of new C–C bonds in the place of C–X bonds (X = O, N, and S), *i.e.*, (A) oxy-Claisen rearrangement, (B) Ireland–Claisen rearrangement, (C) aza-Claisen rearrangement, (D) thio-Claisen rearrangement/sulfonium rearrangement, and (E) Saucy–Marbet rearrangement.

### 2.1. [3,3]-Sigmatropic rearrangement

Since its discovery in 1912, [3,3]-Claisen rearrangement reactions have been widely utilised in the synthesis of complex molecules.^4^ The technique has been integrated into numerous synthetic pathways, leading to a wide variety of natural products, and has proven useful for incorporating vicinal stereocentres with high stereoselectivity. The [3,3]-Claisen rearrangement not only provides access to newly formed C–C bonds from C–X bonds but also has the potential to form two contiguous stereocentres. A list of an enormous number of studies has been published in this area; this section emphasises important concepts for the diastereoselective and enantioselective [3,3]-Claisen rearrangement of ynamides involving the formation of new C–C bonds with concomitant cleavage of C–X (X = N, O, and S) bonds.

#### 2.1.1. Oxy-Claisen rearrangement

Over the last decade, the most important aspect of the [3,3]-sigmatropic rearrangement of ynamides has been the involvement of allylic alcohols as nucleophilic partners, which has resulted in the formation of carbonyl group-containing compounds along with vicinal stereocentres. These compounds are currently used as building blocks in the synthesis of complex natural products and pharmaceuticals.^3*c*^ The enolate-like chemistry behind this reaction has drawn the attention of the synthetic community significantly towards this field of research. Herein, we detail every accomplishment made thus far in the field of the oxy-Claisen rearrangement of ynamides.

**Scheme 1:**
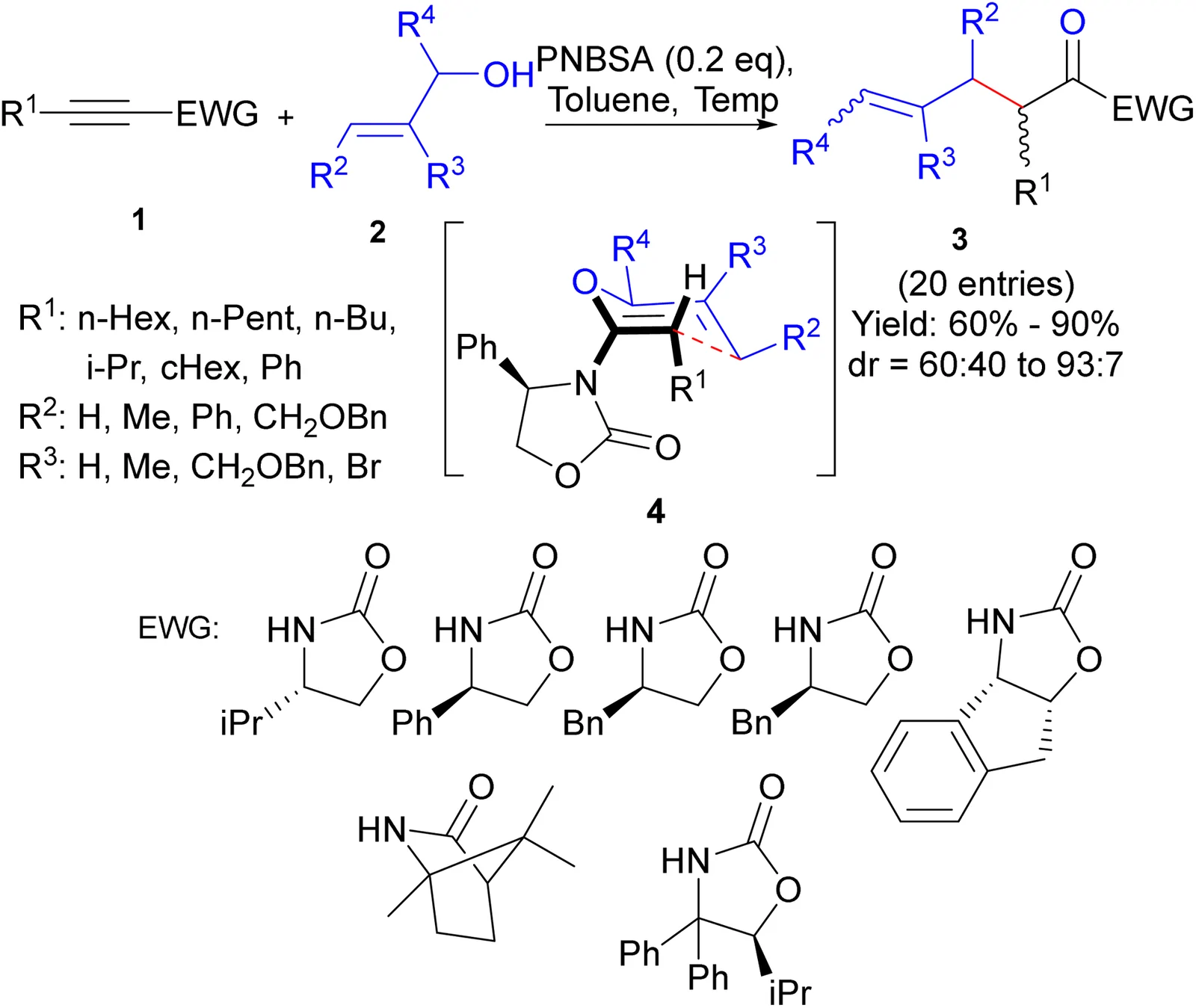
PNBSA-catalysed Ficini–Claisen rearrangement.

In 2002, the Hsung group^10^ presented the first stereoselective Ficini–Claisen rearrangement that furnishes chiral γ,δ-unsaturated amides 3 with vicinal tertiary–tertiary stereocentres *via* a diastereoselective rearrangement of chiral ynamides 1 and allylic alcohols 2, promoted by *p*-nitrobenzenesulfonic acid (PNBSA) catalysis (Scheme 1). This process yields a wide range of chiral moieties and lactones and proceeds smoothly with good diastereoselectivity. The plausible mechanistic pathway begins with the protonation of the ynamides with the Brønsted acid PNBSA, generating a keteniminium intermediate. This intermediate then reacts with the nucleophilic partner, allyl alcohol 2, to produce an allyl vinyl ether intermediate. The reaction then proceeds through a [3,3]-Claisen rearrangement to produce the appropriate chiral products in good to excellent yield and diastereoselectivity 4 (Scheme 1).

**Scheme 2:**
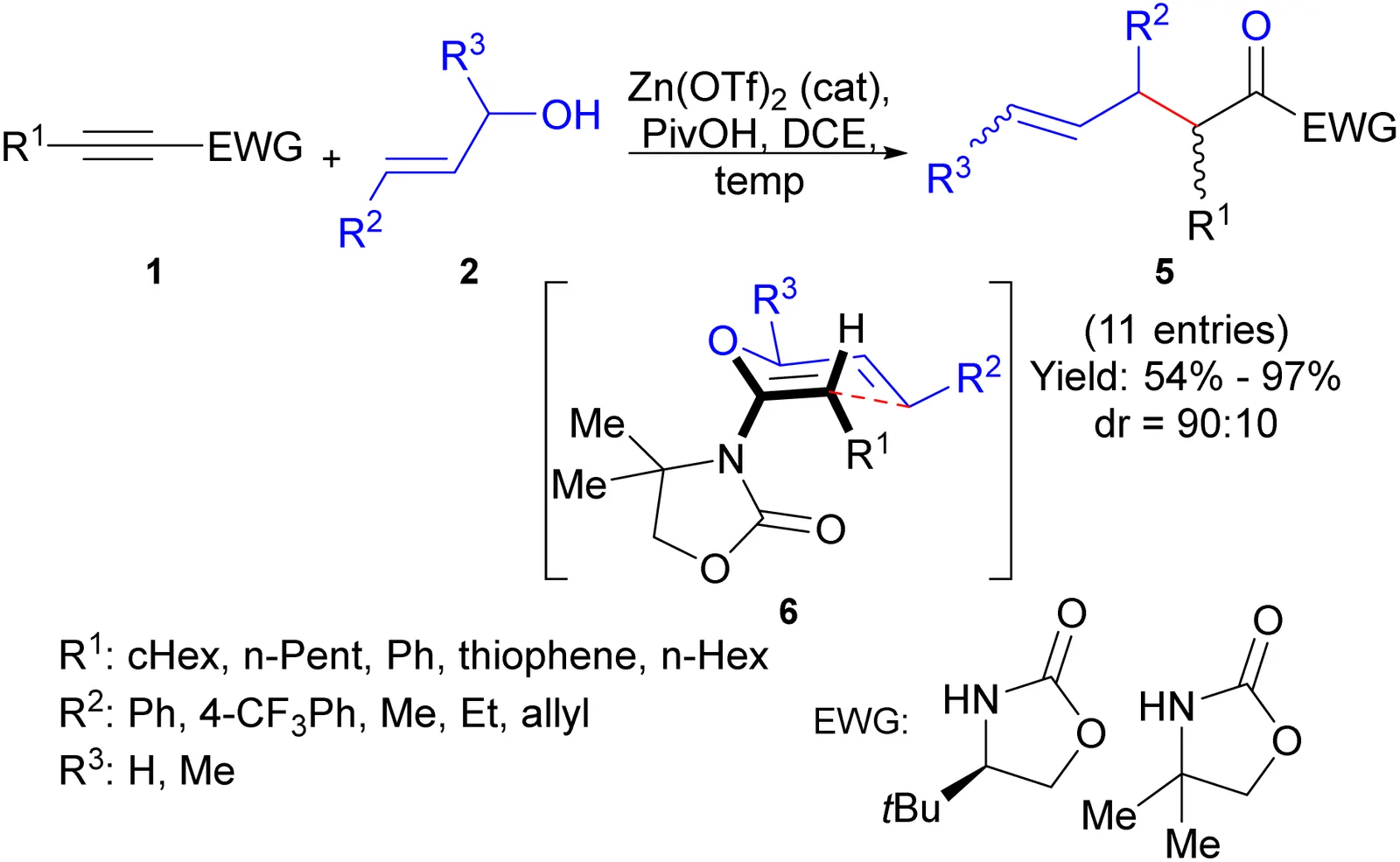
Zn-catalysed Claisen rearrangement of ynamides.

In 2010, Gaunt and co-workers^11^ developed a transition metal-catalysed *syn*-selective hydroalkoxylation reaction, followed by a [3,3]-sigmatropic rearrangement reaction under acidic conditions, resulting in α,β-disubstituted imides with 54–97% yield and good diastereoselectivity (Scheme 2). This procedure was applied to an achiral ynamide system in the presence of 10 mol% Zn(OTf)_2_ and 50 mol% *t*-BuCO_2_H at room temperature.

At the same time, the unsubstituted auxiliary system containing ynamides failed to give the desired adduct due to the complete hydrolysis of the starting material under the same reaction conditions. The *in situ* generated TfOH (from Lewis acid Zn(OTf)_2_ and allylic alcohol) activates the ynamides, which react with alcohol to produce transition state 6. Subsequent Claisen rearrangement provides a γ,δ-unsaturated amide with vicinal tertiary–tertiary stereocentres. The stereochemical result is consistent with the non-chelated Zimmerman–Traxler transition state 6.

**Scheme 3:**
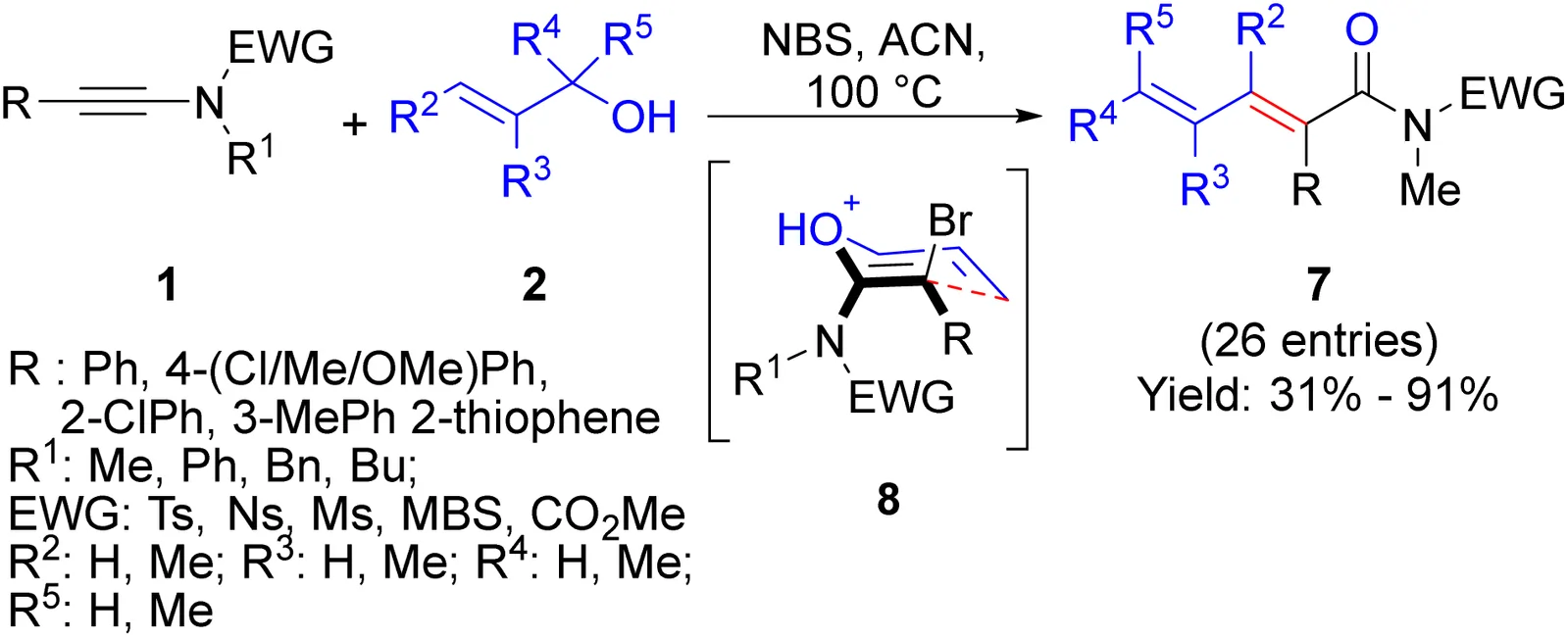
NBS-catalysed Claisen rearrangement of ynamides.

In 2015, Zhai and co-workers revealed a cascade reaction sequence involving the NBS-promoted hydroalkoxylation of ynamides, Claisen rearrangement, and a dehydrobromination reaction process (Scheme 3).^12^ The reaction provided an efficient and straightforward method for the synthesis of (2*Z*)-2,4-dienamides, which have been used as important intermediates in organic synthesis. Owing to their enhanced nucleophilicity, feasible synthesis and easy handling, the authors chose ynsulfonamides as the ideal substrates instead of lactam- or oxazolidinone-containing ynamides. The reaction proceeds through the nucleophilic attack of the bromine atom of NBS on the ynamides, resulting in a keteniminium intermediate, followed by the nucleophilic addition of allylic alcohol to furnish transition state 8, which undergoes Claisen rearrangement, leading to the formation of α-aryl-α-bromo-γ,δ-unsaturated amide. This transition state 8 afforded the desired product (*2Z*)-2,4-dienamides 7 by the elimination of the HBr molecule through a *trans*-coplanar conformation. The (*Z*)-configuration of the final adduct was confirmed by NOESY analysis of the specified protons.

**Scheme 4:**
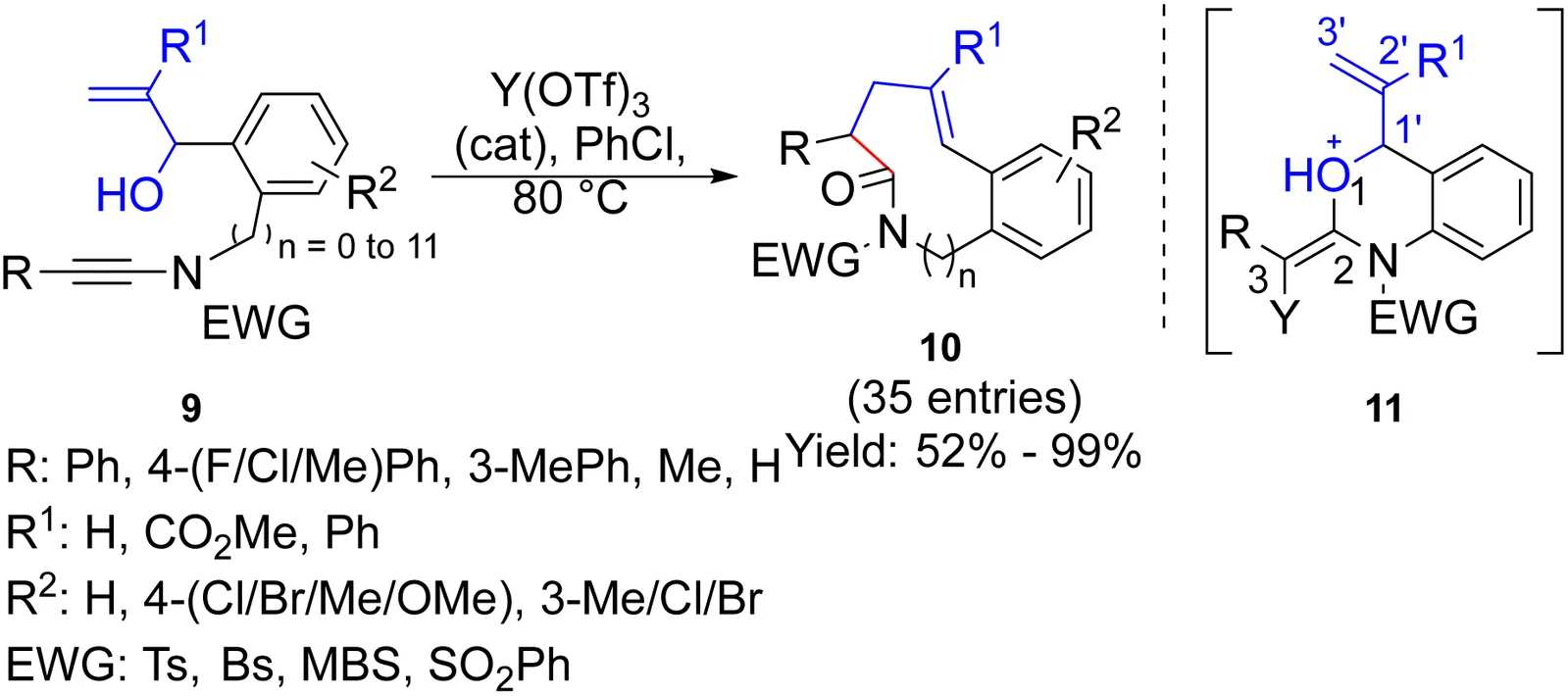
Yttrium-catalysed intramolecular Claisen rearrangement of ynamides.

Lewis acid-catalysed intramolecular hydroalkoxylation, followed by a Claisen rearrangement reaction of ynamide 9, was presented by L. W. Ye *et al.* in 2017 for the effective synthesis of valuable medium-sized lactams (Scheme 4).^13^ The reaction was observed in the presence of the Y(OTf)_3_ catalyst upon heating at 80 °C in toluene for 2 h. This protocol was successfully applied to a variety of substituted ynamides, as well as terminal ynamides, to deliver the desired 8–19-membered medium-lactams and macrocycles with yields ranging from 52% to 99%.

The resulting lactams were further treated in basic media such as SmI_2_, NaBH_4_/I_2_, and LiAlH_4_ to prepare synthetically useful molecules in good yields. The plausible mechanism starts with the attack of the hydroxyl group on the yttrium-activated ynamide to produce vinyl-yttrium intermediate 11, which undergoes subsequent proton transfer and Claisen rearrangement reactions to furnish adduct 10.

**Scheme 5:**
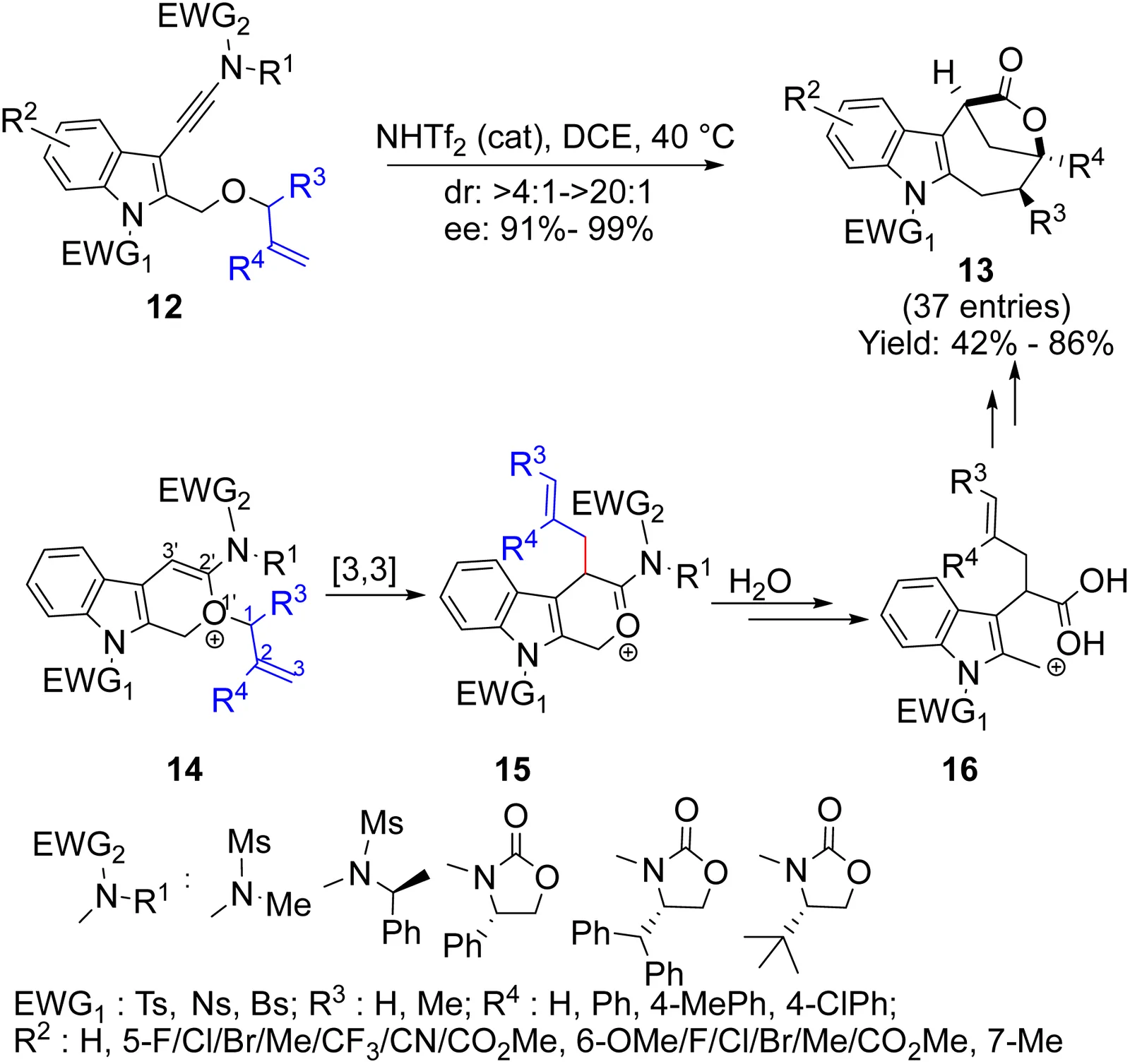
Brønsted acid-catalysed tandem intramolecular alkoxylation/Claisen rearrangement.

In 2019, Ye and co-workers introduced a metal-free, Brønsted acid-catalysed tandem intramolecular alkoxylation/Claisen rearrangement, followed by alkene carbo-oxygenation for the synthesis of bridged [4,2,1] lactones 13 (Scheme 5).^14^ The authors chose an indole-tethered ynamide as the model substrate for this transformation. Both electron-donating and -withdrawing groups on the indolyl unit of the ynamide were well tolerated, yielding the desired product in good to excellent yields. The use of benzofuran-, pyrrole-, and alkoxy-arene ring-containing ynamides also afforded the corresponding products with optimal yields and dr values. The indole-tethered ynamide is protonated by a Brønsted acid, followed by attack of the alkoxy group to form Claisen rearrangement precursor 14*via* a keteniminium intermediate, which undergoes a subsequent [3,3]-sigmatropic rearrangement to yield intermediate 15. Thereafter, in the presence of a trace amount of water, it was rearranged to various bridged [4,2,1] lactones.

**Scheme 6:**
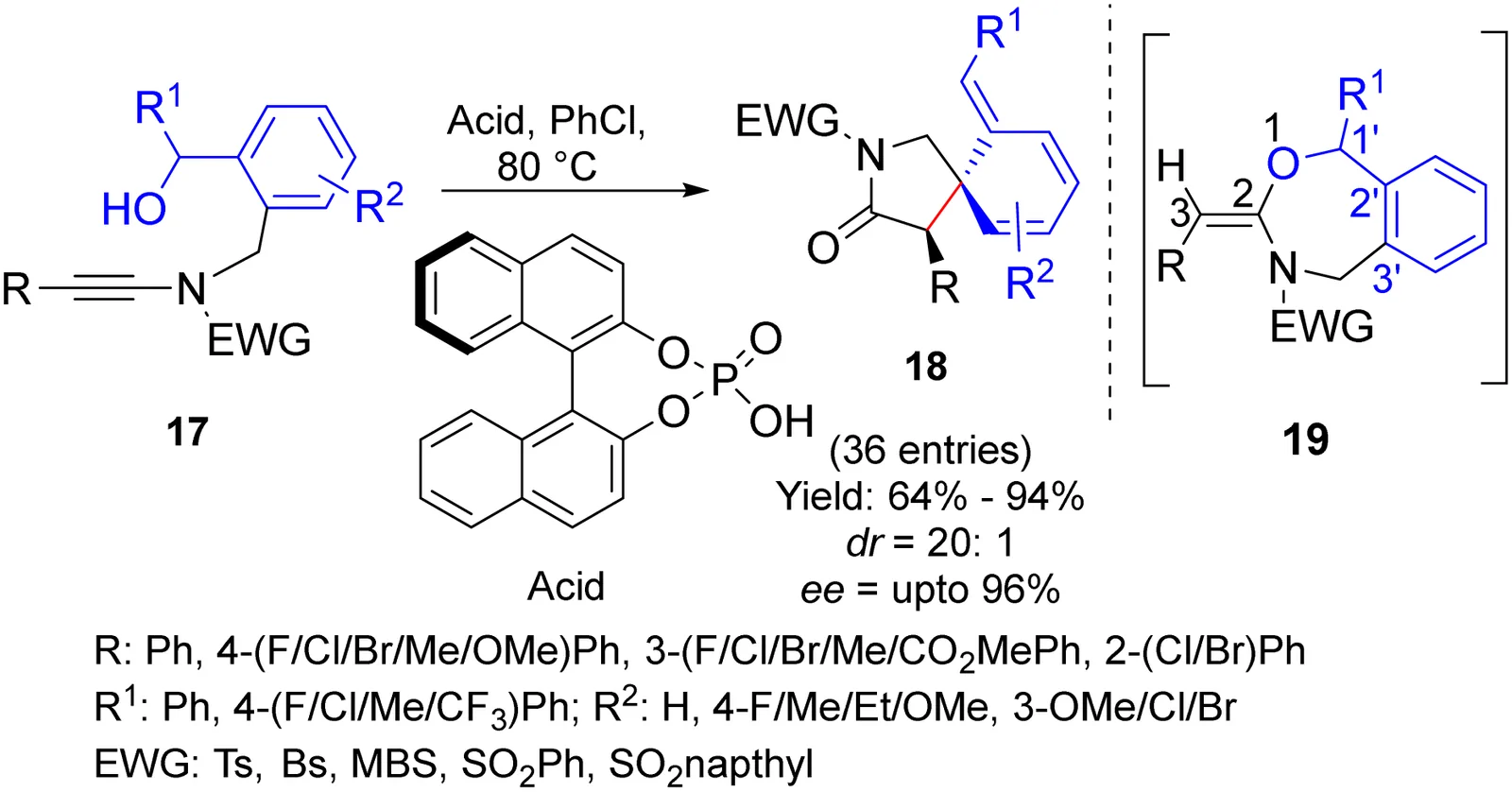
Acid-catalysed intramolecular dearomative Claisen rearrangement of ynamides.

A similar intramolecular hydroalkoxylation, followed by a Claisen rearrangement reaction for the diastereo- and enantioselective synthesis of valuable spirolactams *via* a Brønsted acid-catalysed dearomatization process, was introduced by the same group in 2021 (Scheme 6).^15^ With good to exceptional yields and excellent diastereoselectivities, the reaction produced an environmentally friendly, atom-economic synthesis of a wide range of substrates using a phosphoric acid catalyst. However, chiral phosphoric acid was also employed efficiently to achieve the asymmetric version of this tandem dearomatisation reaction with an excellent enantiomeric ratio. The reaction proceeds through intermediate 19.

**Scheme 7:**
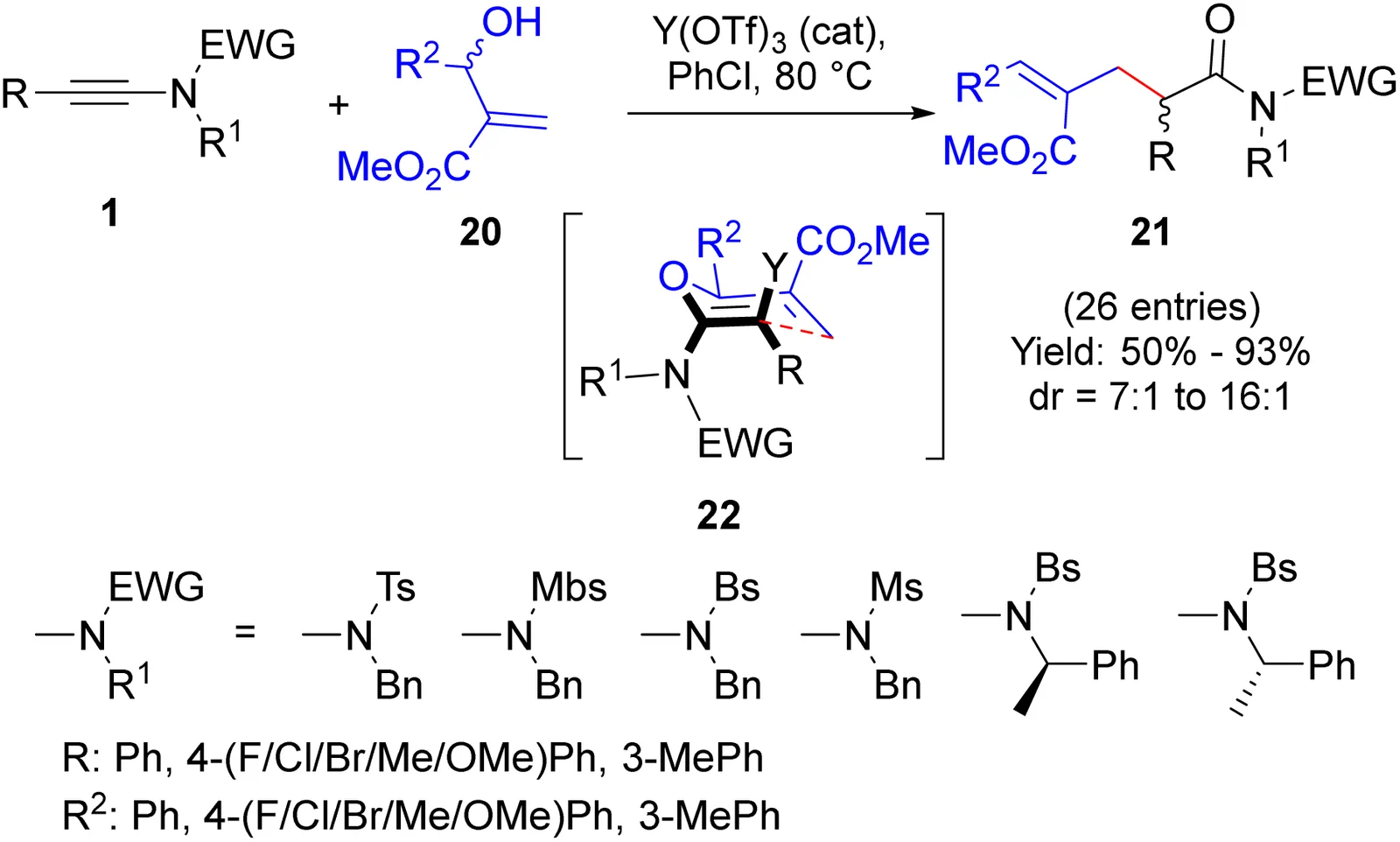
Yttrium-catalysed intermolecular Claisen rearrangement of ynamides.

Chiral ynamides are also used as great starting materials for the above tandem hydroalkoxylation/Claisen rearrangement sequence. To achieve this task for the generation of γ,δ-unsaturated amides, the same group explored a yttrium-catalysed intermolecular diastereoselective method by selecting MBH alcohol as the alkoxylation partner (Scheme 7).^16^ Both chiral and achiral ynamides and alcohols were used to obtain the intended product with vicinal tertiary stereocenters in good yields and moderate to good diastereoselectivities. After treatment with LiOH, the resultant target molecule was easily transformed into the corresponding synthetically valuable chiral carboxylic acids. The reaction proceeds *via* activation of the ynamide through coordination of yttrium triflate to the triple bond, followed by nucleophilic attack of allylic alcohol to generate vinyl-yttrium transition state 22, which subsequently undergoes a [3,3]-Claisen rearrangement reaction.

**Scheme 8:**
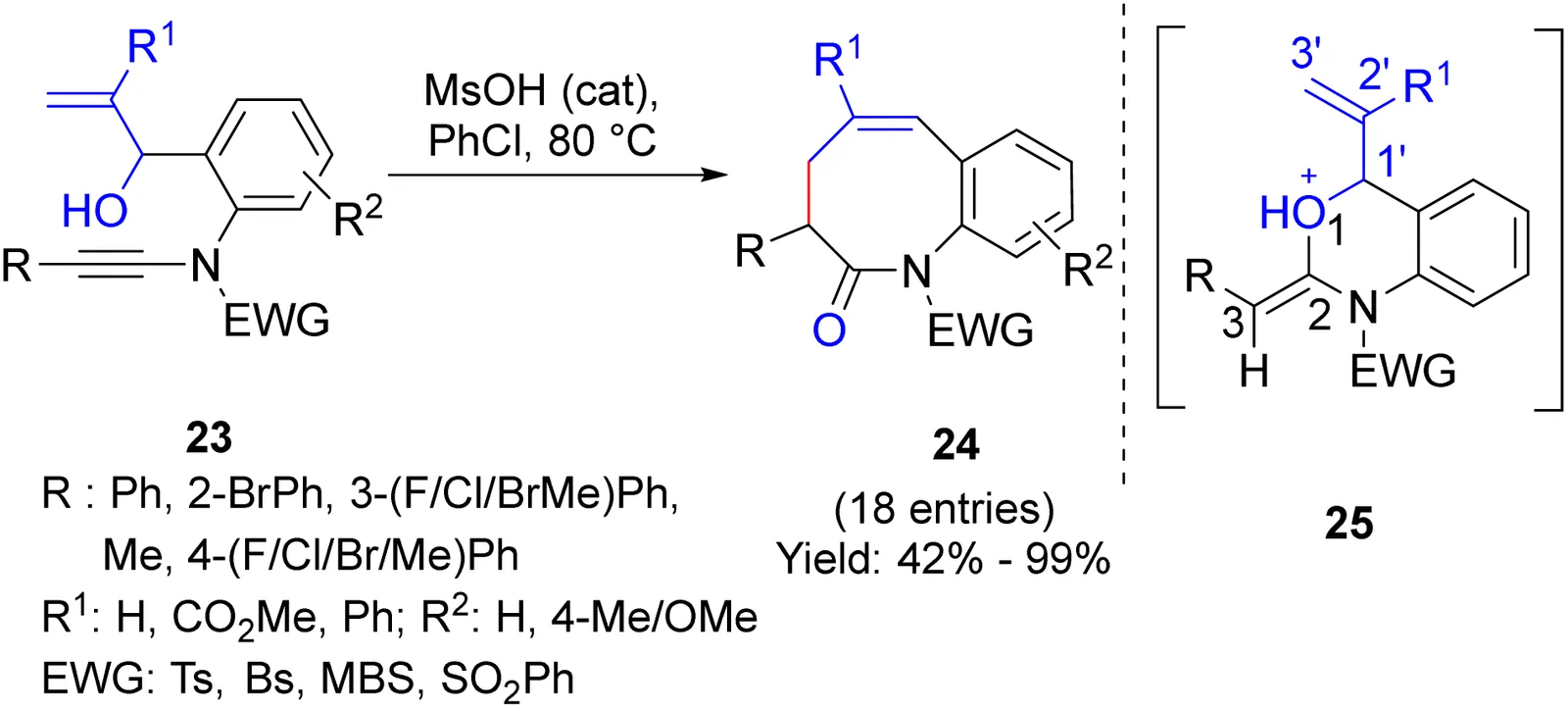
Brønsted acid-catalysed intramolecular Claisen rearrangement of ynamides.

Inspired by their previous studies^13–15^ on the intramolecular hydroalkoxylation/Claisen rearrangement reaction of ynamides, in 2023, L. W. Ye and co-workers reported a Brønsted acid-catalysed method for the synthesis of biologically important eight-membered lactams (Scheme 8).^17^ The final products were obtained in moderate to good yields. The reaction proceeds through intermediate 25. The authors also performed a kinetic resolution study of ynamides using phosphorus-based chiral Brønsted acids, which resulted in the formation of chiral eight-membered lactams with 35–88% enantiomeric excess.

**Scheme 9:**
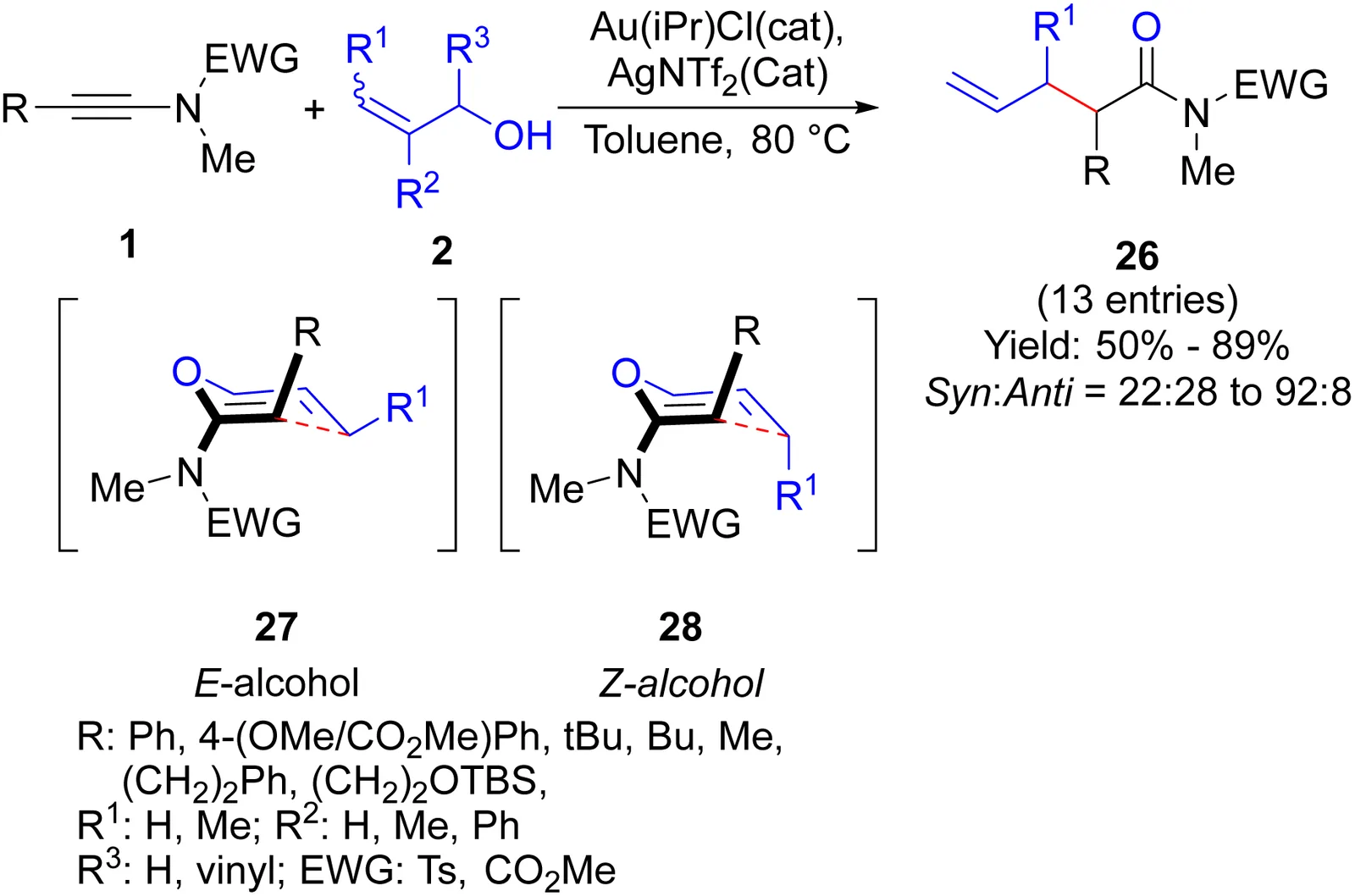
Au-catalysed Claisen rearrangement of ynamides.

Sato and co-workers developed a novel Au(i)-catalysed cascade hydroalkoxylation/Claisen rearrangement reaction of ynamides with allylic alcohols (Scheme 9).^18^ The reaction involves the regioselective attack of allylic alcohol on the gold-activated ynamide, followed by a Claisen rearrangement reaction to give γ,δ-unsaturated amides in good to excellent yield. The [Au(IPr)NTf_2_] (1 mol%) catalyst gave optimum results in this methodology rather than the use of a mixture of [Au(IPr)Cl] (1 mol%) and AgNTf_2_ (1 mol%). Optimised reaction conditions were applied to a variety of alkyl/aryl-substituted ynamides and simple/branched allylic alcohols to furnish the corresponding products in good yields. The use of (*E*)- or (*Z*)-allylic alcohols resulted in the formation of *anti*- or *syn*-products as the major products through transition states 27 or 28, respectively.

**Scheme 10:**
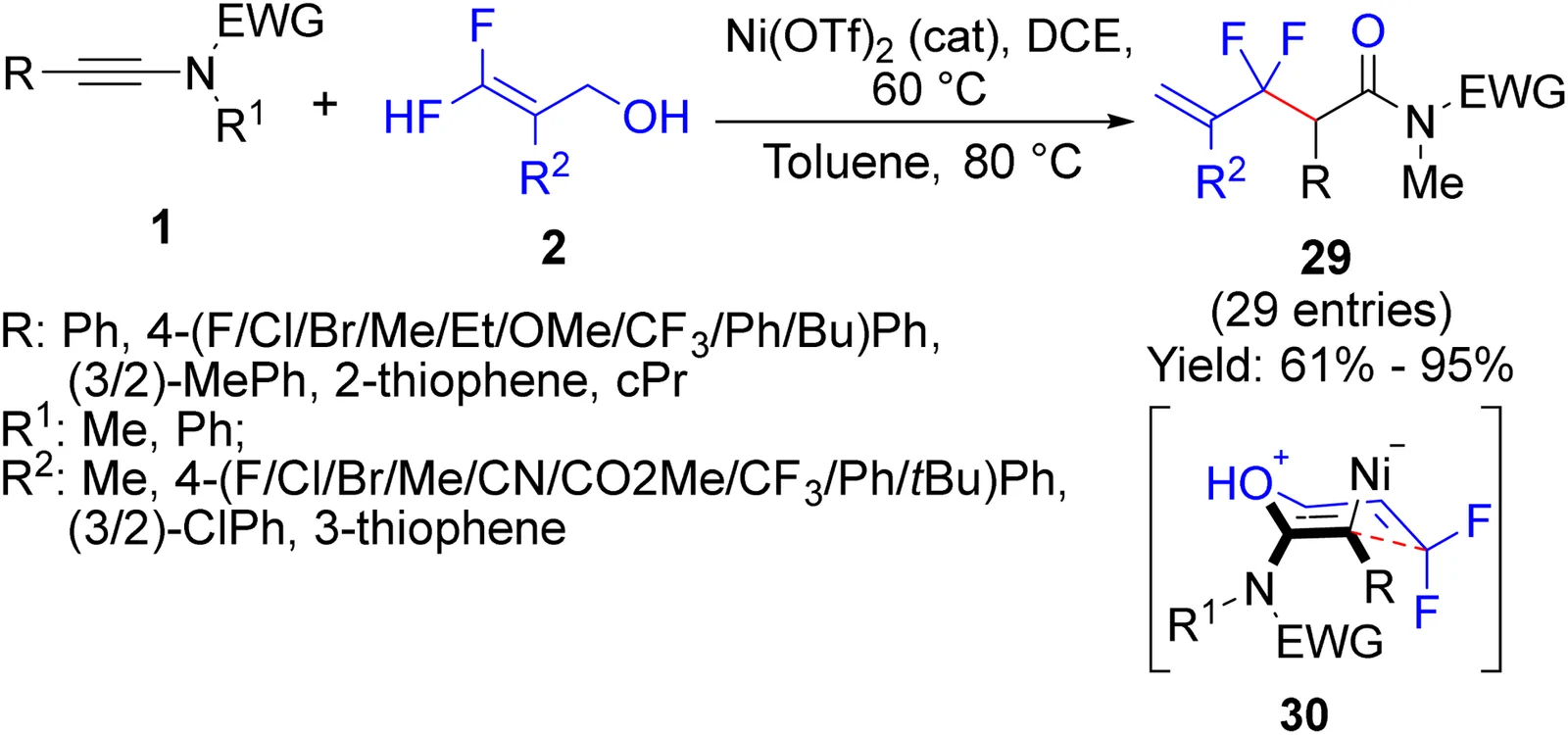
Ni-catalysed Claisen rearrangement of ynamides.

In 2024, Jiang and co-workers demonstrated a Lewis acid-catalysed tandem alkoxylation/Claisen rearrangement reaction of ynamides for the synthesis of β,β-difluoromethylenamide derivatives *via* an oxy-difluoroallylation process (Scheme 10).^19^ For the first time, the authors used 3,3-difluoroallyl alcohols to construct fluorine-containing building blocks. The reaction offers mild conditions, atom economy, good tolerance of functional groups, and remarkable yields. By employing 10 mol% of Ni(OTf)_2_, the triple bond of ynamide was activated, generating a keteniminium intermediate, which underwent nucleophilic attack by 3,3-difluoroallyl alcohol, generating vinyl-nickel transition state 30, followed by Claisen rearrangement to give the desired β,β-difluoromethylenamide 29 in 61–95% yield.

**Scheme 11:**
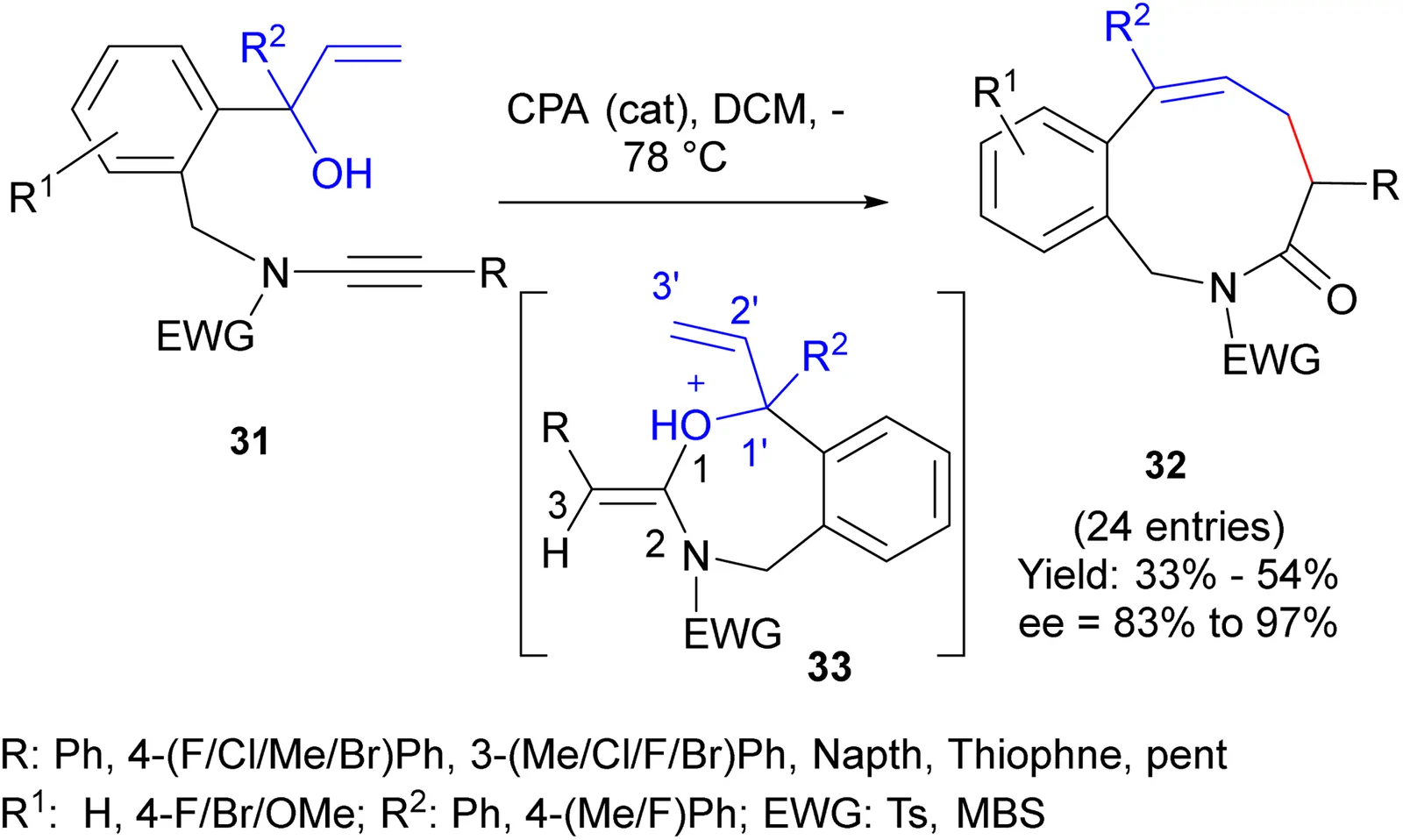
Bronsted-acid-catalysed alkyne functionalisation.

Recently, in 2025, Ye and co-workers developed a Brønsted-acid-catalysed alkyne functionalisation through a kinetic resolution study of tertiary alcohols (Scheme 11).^20^ The authors reported a catalytic asymmetric transformation of tertiary alcohol-tethered ynamides 31 in the presence of chiral phosphoric acid (CPA) catalysts. This led to the formation of the respective chiral nine-membered lactams 32 in moderate to good yields with 75–97% enantiomeric ratios. A wide variety of alkyl- and aryl-substituted ynamides (bearing both electron-withdrawing and electron-donating groups) reacted smoothly under optimised reaction conditions. The attempt to prepare eight-membered lactams using this protocol was unsuccessful. The reaction proceeds through a tandem intramolecular hydroalkoxylation and [3,3]-sigmatropic rearrangement pathway to deliver the desired cyclic lactams 32 through intermediate 33.

**Scheme 12:**
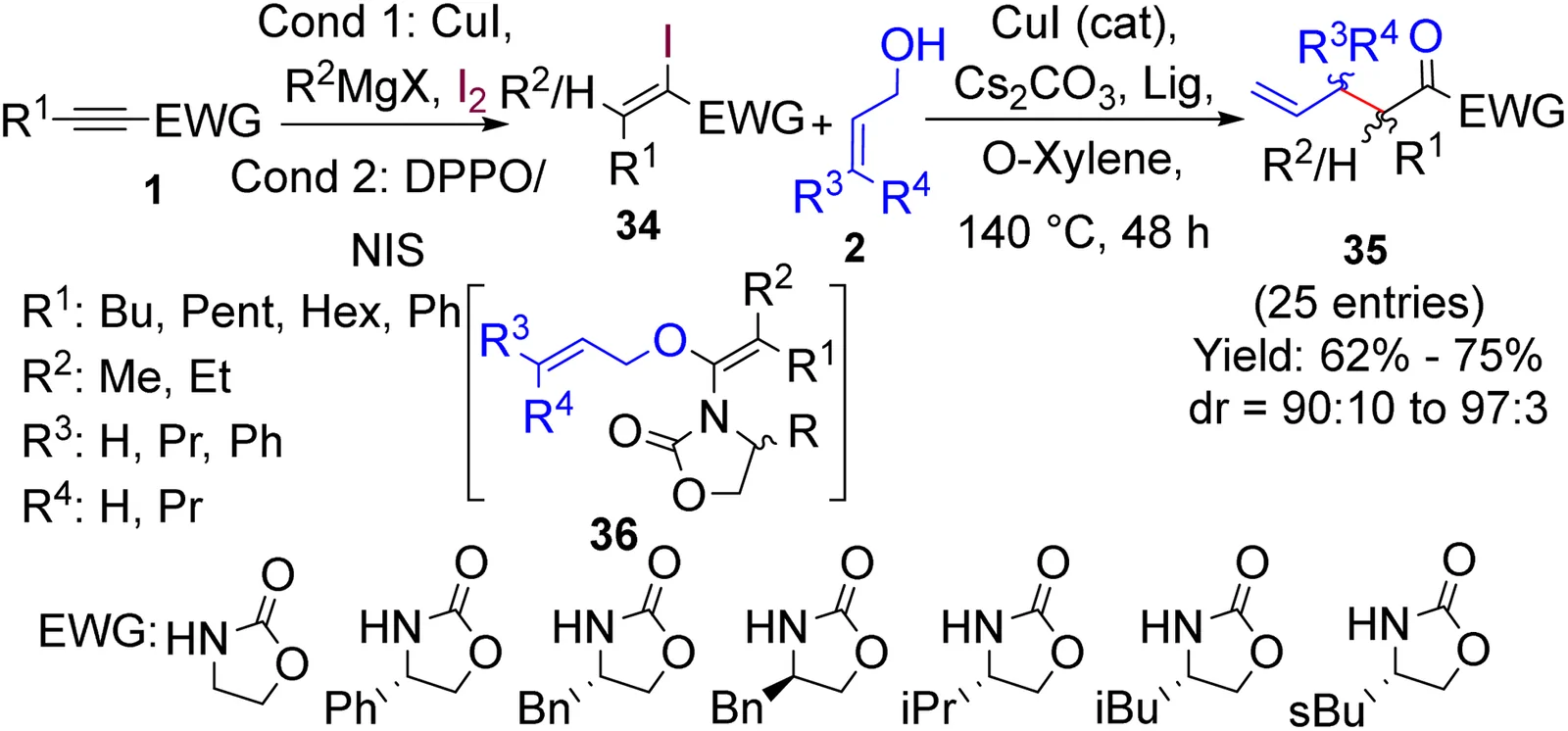
Cu(i)-catalysed stereoselective Claisen rearrangement of ynamides.

Very recently, in 2025, our group reported a Cu(i)-catalysed stereoselective Claisen rearrangement, starting from ynamides to form vicinal stereocentres bearing the desired all-carbon quaternary stereocentres (Scheme 12).^21^ The reaction involves stereocontrolled carbocupration/iodination or hydroiodination (using DPPO/NIS) of ynamides to form β,β-disubstituted α-haloenamide or β-monosubstituted α-haloenamide, respectively, which serve as precursors for the Claisen rearrangement reaction. The resulting vinyl iodide undergoes Ullman-type coupling with the allyl alcohol in the presence of a copper ligand complex to generate allyl vinyl ether intermediate 36. This intermediate 36, in the subsequent Claisen rearrangement reaction at high temperature, afforded the final product 35 with high diastereoselectivity but in moderate to good yields. A wide range of ynamides 34 and allylic alcohols were successfully used to describe the productivity of the optimised reaction conditions. Surprisingly, the use of (*E*)- or (*Z*)-allylic alcohols gave the same diastereomers, as confirmed *via* NMR and specific rotation analyses. The resulting products were further converted to chiral alcohols, chiral decarbonylated products, and chiral oxazolines with greater enantiomeric purity, showing the versatility of the reaction methodology. This is the only method in this category that provides vicinal all-carbon quaternary and tertiary stereocentres in an acyclic framework.

#### 2.1.2. Ireland–Claisen rearrangement

Sigmatropic Ireland–Claisen rearrangements are commonly employed in the synthesis of natural products,^22^ offering synthetic versatility that enables the generation of congested stereocentres with excellent stereocontrol. This method highlights the generation of silyl ketene acetals from esters by treatment with a strong base, which are rather unstable and spontaneously rearrange at room temperature without the need for an additional catalyst. Although this field of study is well established and involves a variety of alkenes and alkynes, there has been only one documented instance of the utilisation of ynamides in the Ireland–Claisen rearrangement to date.

**Scheme 13:**
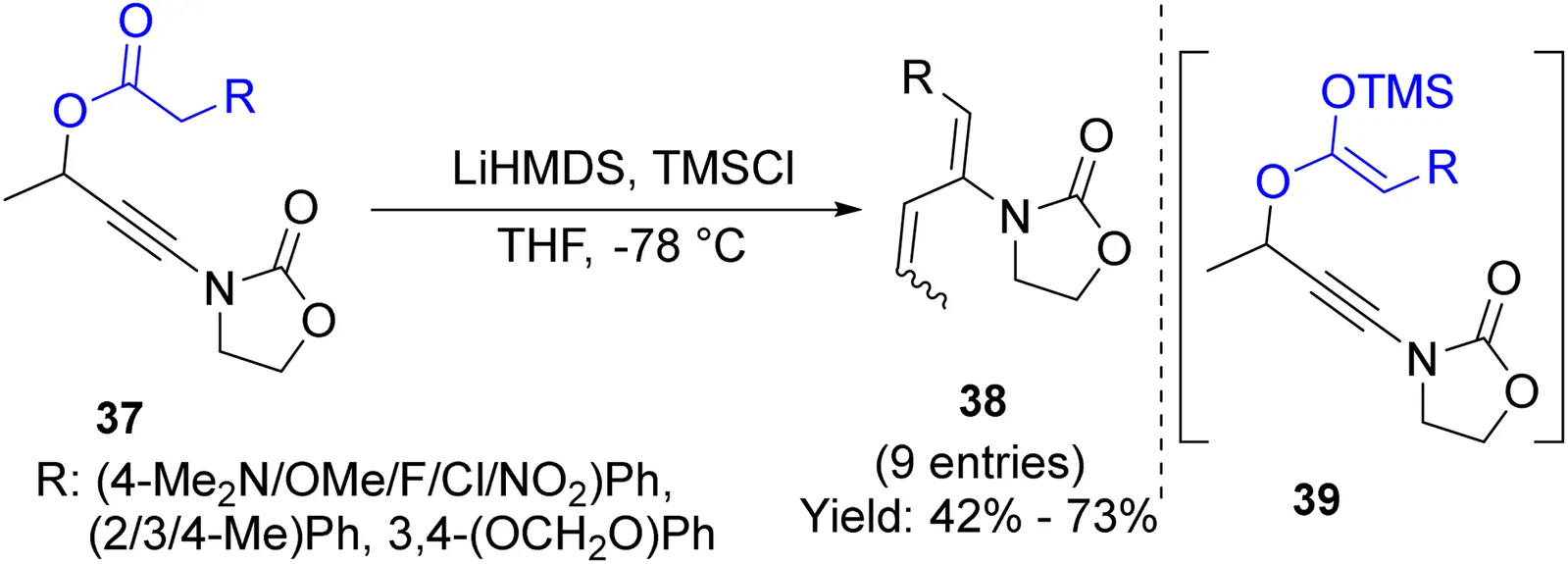
Ireland–Claisen rearrangement of the ynamido esters.

In 2012, Heffernan and Carbery^23^ introduced the first Ireland–Claisen rearrangement of ynamido esters 37 for the synthesis of a series of trisubstituted amidodienes 38 in good yields and with excellent *E*/*Z* selectivity (Scheme 13). Temperature plays an important role in the synthesis of the target molecule. There was no reaction observed at a temperature of −40 °C or above. Unfortunately, under the conditions of the base-catalysed reaction, they failed to isolate the required allenamide carboxylic acid product. Rather, post-rearrangement decarboxylation produced a substantial yield of amidodiene adducts with excellent stereoselectivity.

However, the reaction requires silyl chloride for a smooth rearrangement process through the formation of transition state 39. The reaction with maleic anhydride was used to investigate whether the resultant diene could be used as a diene component in a Diels–Alder synthesis, but no reaction was observed.

#### 2.1.3. Aza–Claisen rearrangement

The aza-Claisen rearrangement is an important tool for the synthesis of various nitrogen-containing molecules and natural products.^24^ However, it has not attracted the attention of synthetic chemists for a long time due to the formation of undesirable by-products and decomposition of the starting materials under thermal conditions. After the development of metal complex catalysis, it became feasible to reduce both the reaction temperature and reaction time, allowing the transformation to proceed comparatively smoothly to afford the desired products in high yields. This section elaborates the advancements in the aza-Claisen rearrangement involving the generation of keteniminium intermediates, which further rearrange to give the required adduct.

**Scheme 14:**
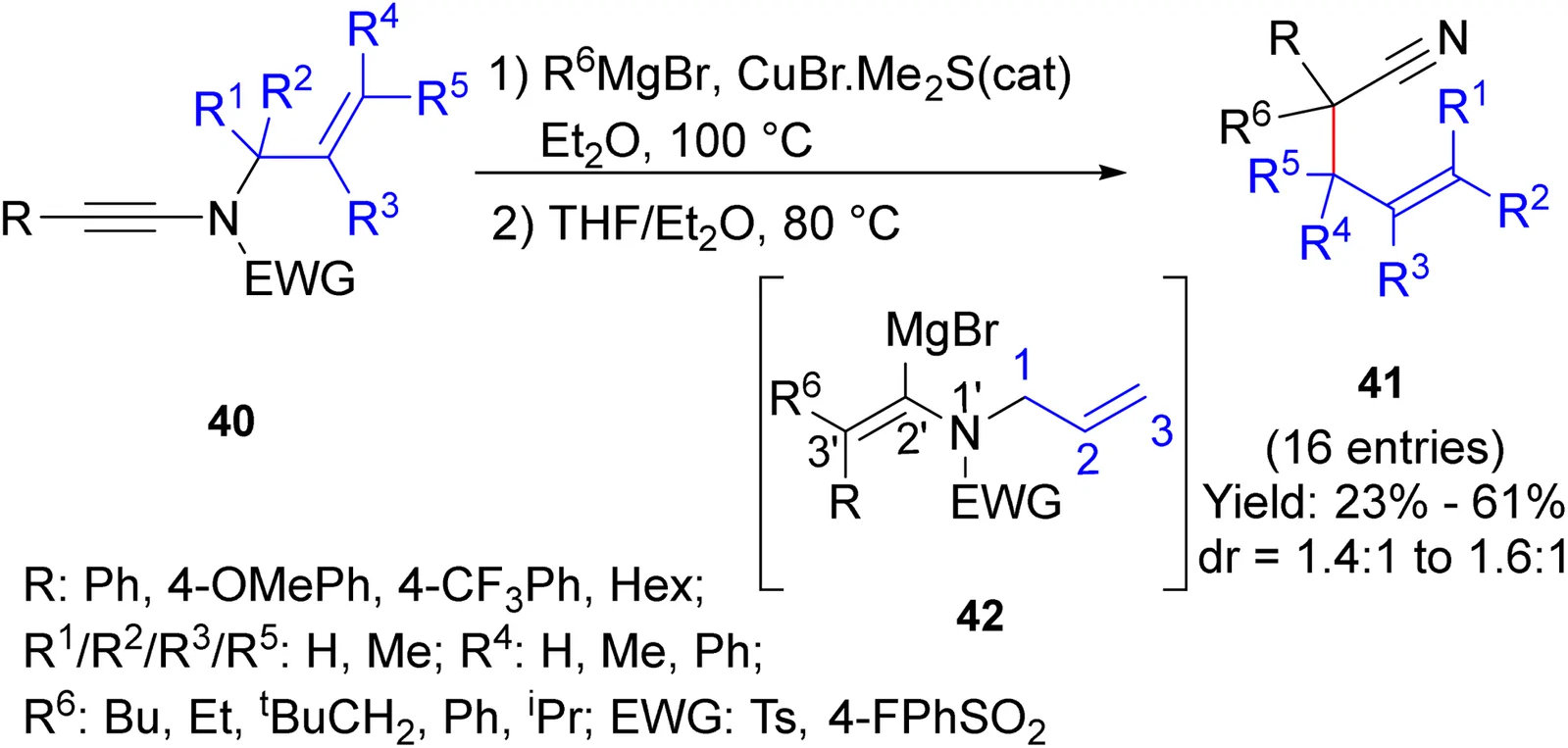
Cu-catalyzed carbomagnesiation reaction of ynamides.

In 2007, Oshima and co-workers proposed a Cu-catalyzed carbomagnesiation reaction of ynamides (Scheme 14),^25^ followed by an aza-Claisen rearrangement for the synthesis of nitriles in moderate to good yields. The ynamide containing an allyl group on N-atom 40, on reaction with the Grignard reagent in the presence of a copper catalyst, underwent a carbomagnesiation reaction regioselectively, resulting in the formation of intermediate 42. Then, subsequent [3,3]-sigmatropic rearrangement, followed by elimination of magnesium sulfinate, delivered the desired nitrile compound 41 with a quaternary centre. However, no result was obtained when the authors tried silyl-cupration, followed by an aza-Claisen rearrangement of ynamides.

**Scheme 15:**
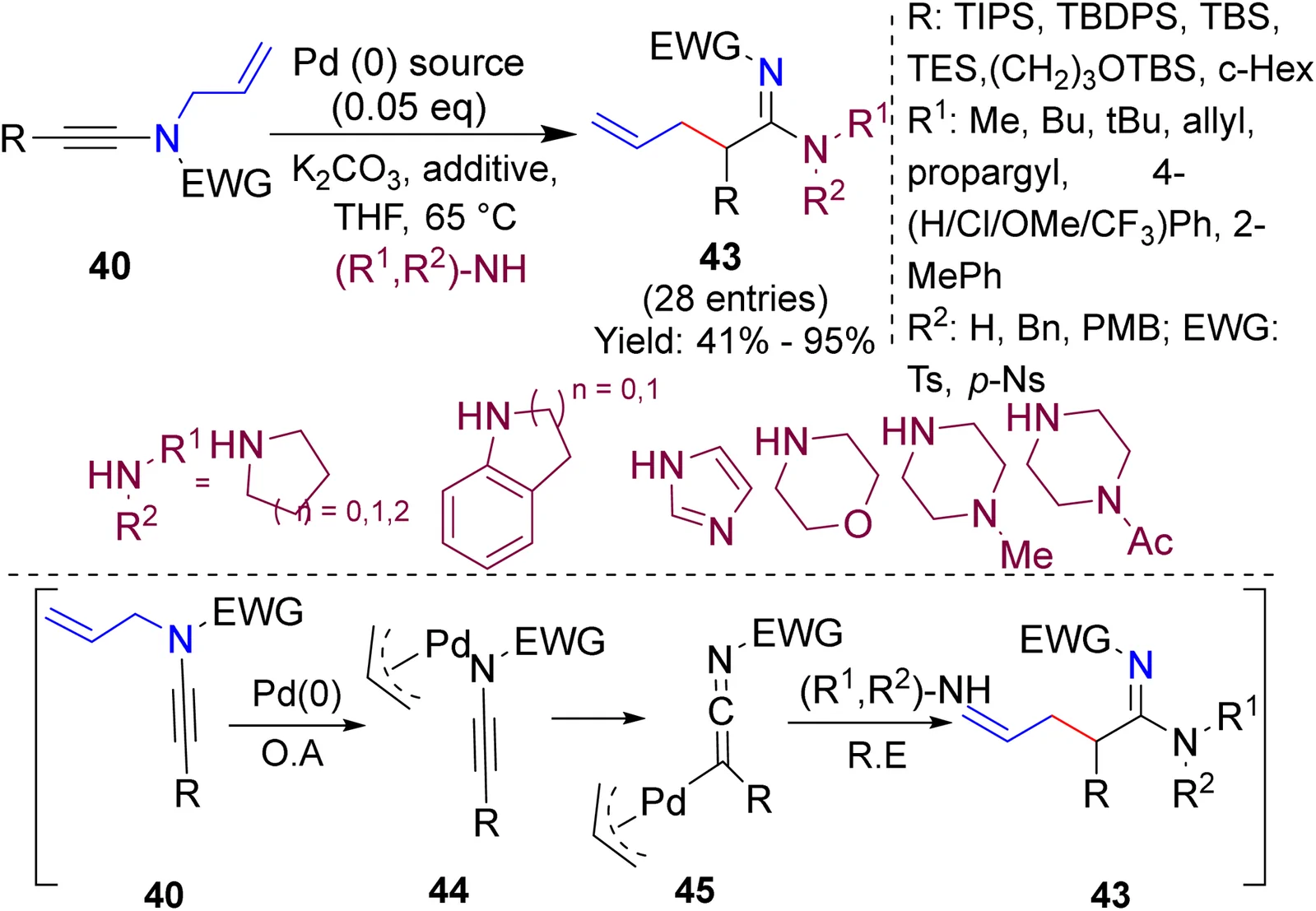
Pd(0)-catalysed aza-Claisen rearrangement of *N*-allylynamides.

In 2009, the first Pd(0)-catalysed aza-Claisen rearrangement of *N*-allylynamides with a detailed mechanistic study was reported by Hsung and co-workers (Scheme 15).^26*a*^ The reaction pathway involves the initial oxidative addition (O.A) of the Pd–L_*n*_ complex to *N*-allylynamides to form ynamido–π-allyl complexes 44 and 45. Through a reductive elimination (R.E) pathway, 45 reacts with 3 equivalents of a primary or secondary amine, resulting in an amidine in moderate to good yields. The variation in the substituents on the ynamides and the temperature mainly determines the productivity of the reaction. A brief mechanistic study was carried out by the same group using various substituents.^26*b*^

**Scheme 16:**
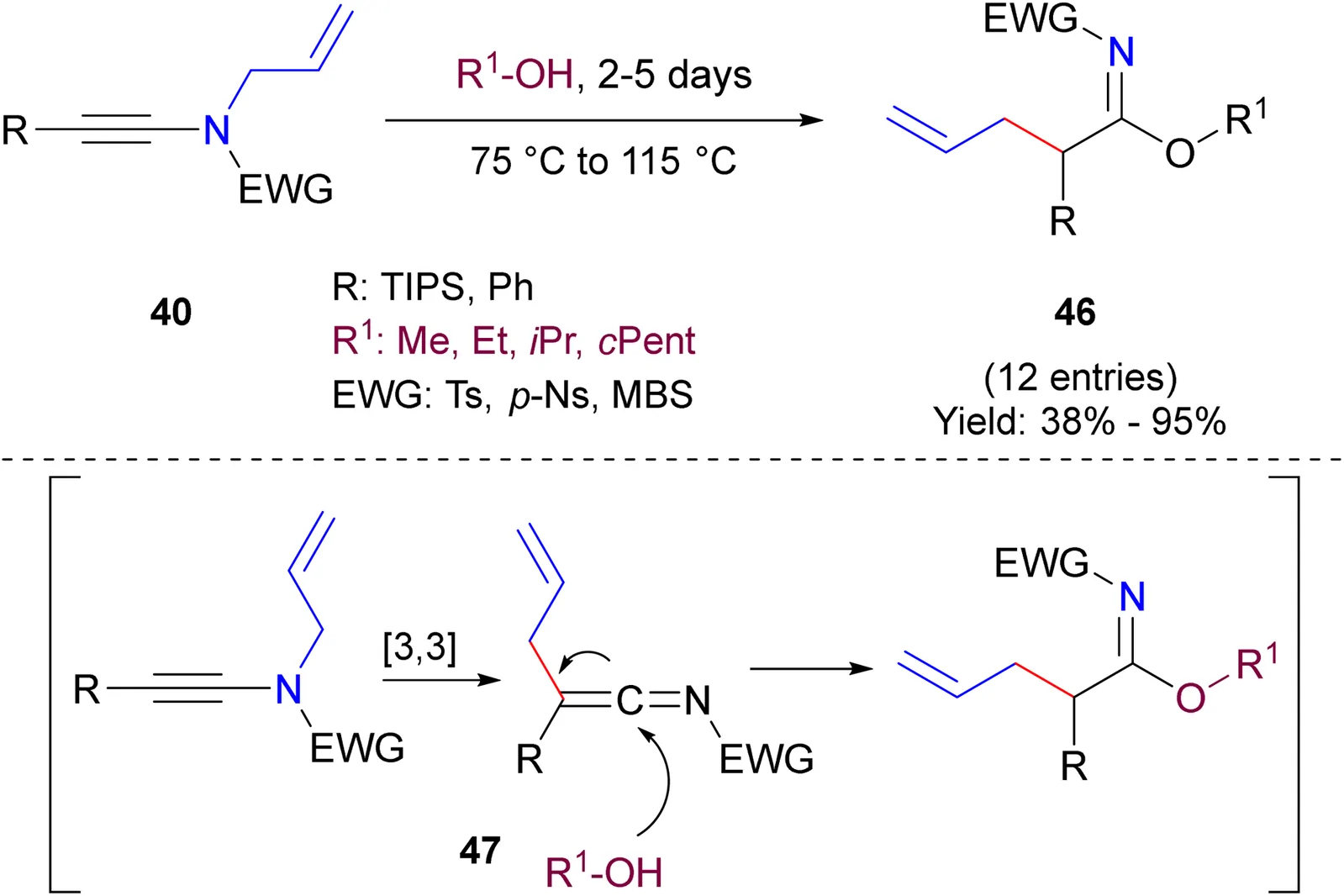
Synthesis of imidates through the aza-Claisen rearrangement of ynamides.

A similar approach was proposed by the same group in 2010 for the synthesis of imidates 46 through an aza-Claisen rearrangement in the absence of any metal–ligand complex (Scheme 16).^27^ The reaction proceeds through the *in situ* formation of keteniminium intermediate 47, which reacts with alcoholic solvents to deliver the desired imidates in moderate to excellent yields. Both silyl- and allyl-terminated ynamides were well tolerated under these reaction conditions, but the *N*-sulfonyl protecting groups, with a strong electronic effect, such as *N*-Boc or *N*-Ac, failed to yield the required products. This indicates that the reaction is sensitive to sterically hindered nucleophiles. Further, the imidates were utilised for the synthesis of azepin-2-ones *via* a ring-closing metathesis reaction. However, the attempt at intramolecular imidate formation through an aza-Claisen rearrangement process was unsuccessful.

**Scheme 17:**
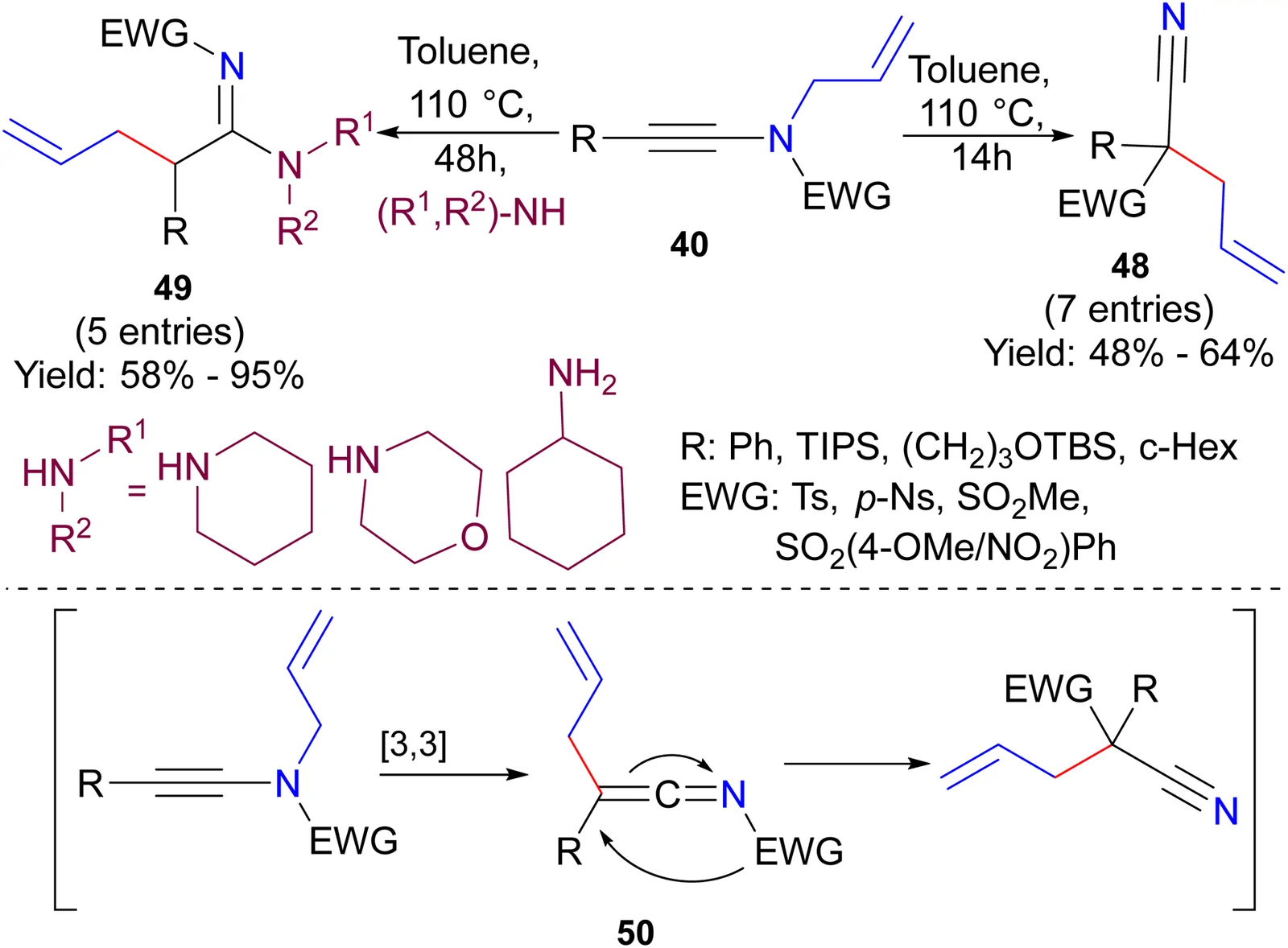
Synthesis of amidines and nitriles *via* the thermal aza-Claisen rearrangement of ynamides.

Later, in 2011, the same group extended their work to the synthesis of nitriles 48 and amidines 49*via* a thermal non-metal-catalysed aza-Claisen rearrangement pathway (Scheme 17).^28^ The substitution on the ynamides and the nucleophilicity of amines played a critical role in product formation. The ynamides resulted in the formation of quaternary nitriles 48 in the absence of amines. This underwent a tandem aza-Claisen rearrangement for the generation of intermediate 50, followed by an N-to-C 1,3-sulfonyl shift pathway. However, in the presence of primary and secondary amines, *N*-allylynamides led to the formation of amidines 49 in excellent yields with wide functional group tolerance. This followed the same route as the formation of imidates, as shown in Scheme 16.

**Scheme 18:**
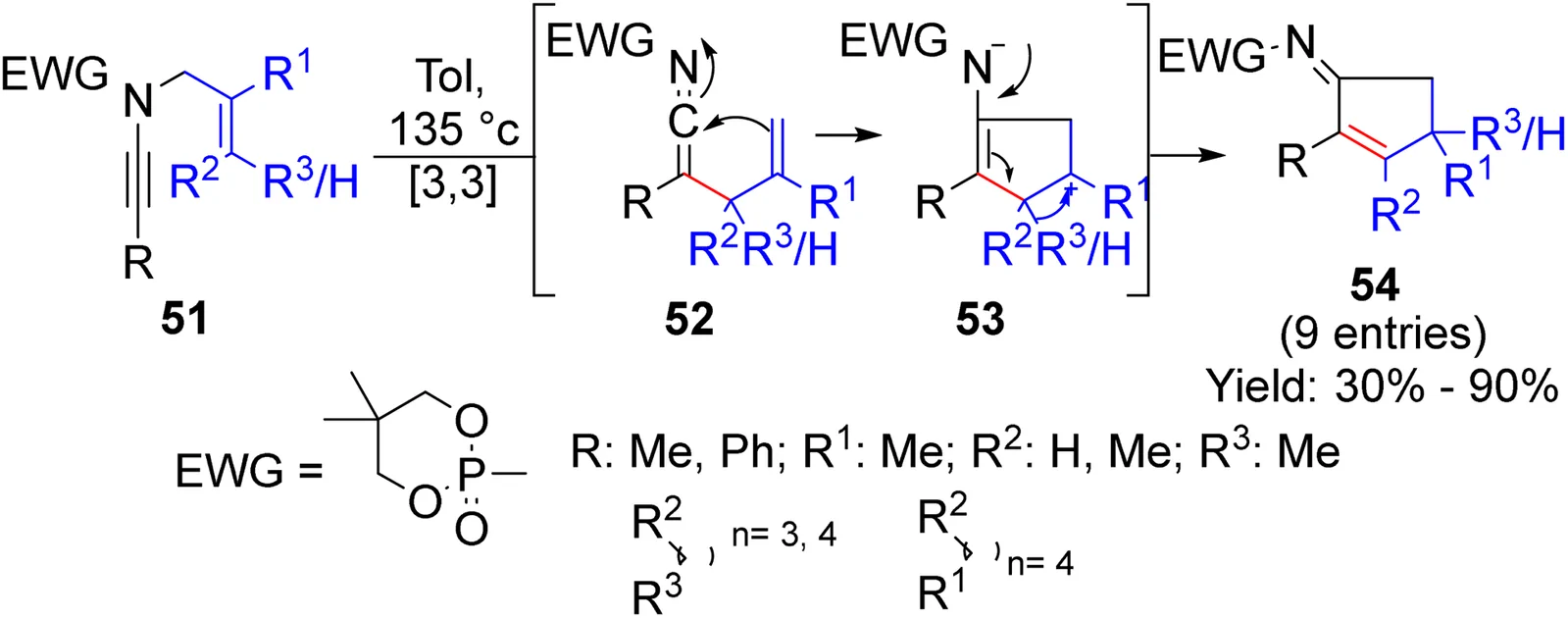
Thermal aza-Claisen rearrangement of *N*-phosphoryl-*N*-allyl-ynamides.

After several achievements in the aza-Claisen rearrangement of *N*-sulfonyl-*N*-allyl-ynamides in previous reports, in 2012, Hsung and co-workers developed a thermal aza-Claisen rearrangement of *N*-phosphoryl-*N*-allyl-ynamides 51 through the carbocyclization cascades of allyl ketenimines for the synthesis of α,β-unsaturated cyclopentenimines 54 (Scheme 18).^29^ Unlike the 1,3-sulfonyl shift in the case of *N*-sulfonyl-*N*-allyl-ynamides, *N*-phosphoryl-*N*-allyl-ynamides underwent an aza-Claisen rearrangement to form allyl ketenimines 52, followed by a carbocyclization pathway, leading to the generation of ionic intermediate 53, which underwent a further Wagner–Meerwein 1,2-H/alkyl shift to yield α,β-unsaturated cyclopentenimines. Detailed observations were made by changing each substituent on the *N*-phosphoryl-*N*-allyl-ynamides. First, it was observed that *N*-prenyl ynamide delivered a 1 : 1 tautomeric mixture of products in excellent yield *via* deprotonation from the α-carbon to enamide instead of the methyl shift. The ynamides bearing methylcyclopentylidine and methylcyclobutylidine resulted in the corresponding spirocyclic product and ring expansion product as the major ones, respectively, due to the increased ring strain in the case of methylcyclobutylidine-containing ynamides. A case of ring contraction was also noticed when a Ph-substituted ynamide with a methylcyclohexene group was treated under the same reaction conditions.

**Scheme 19:**
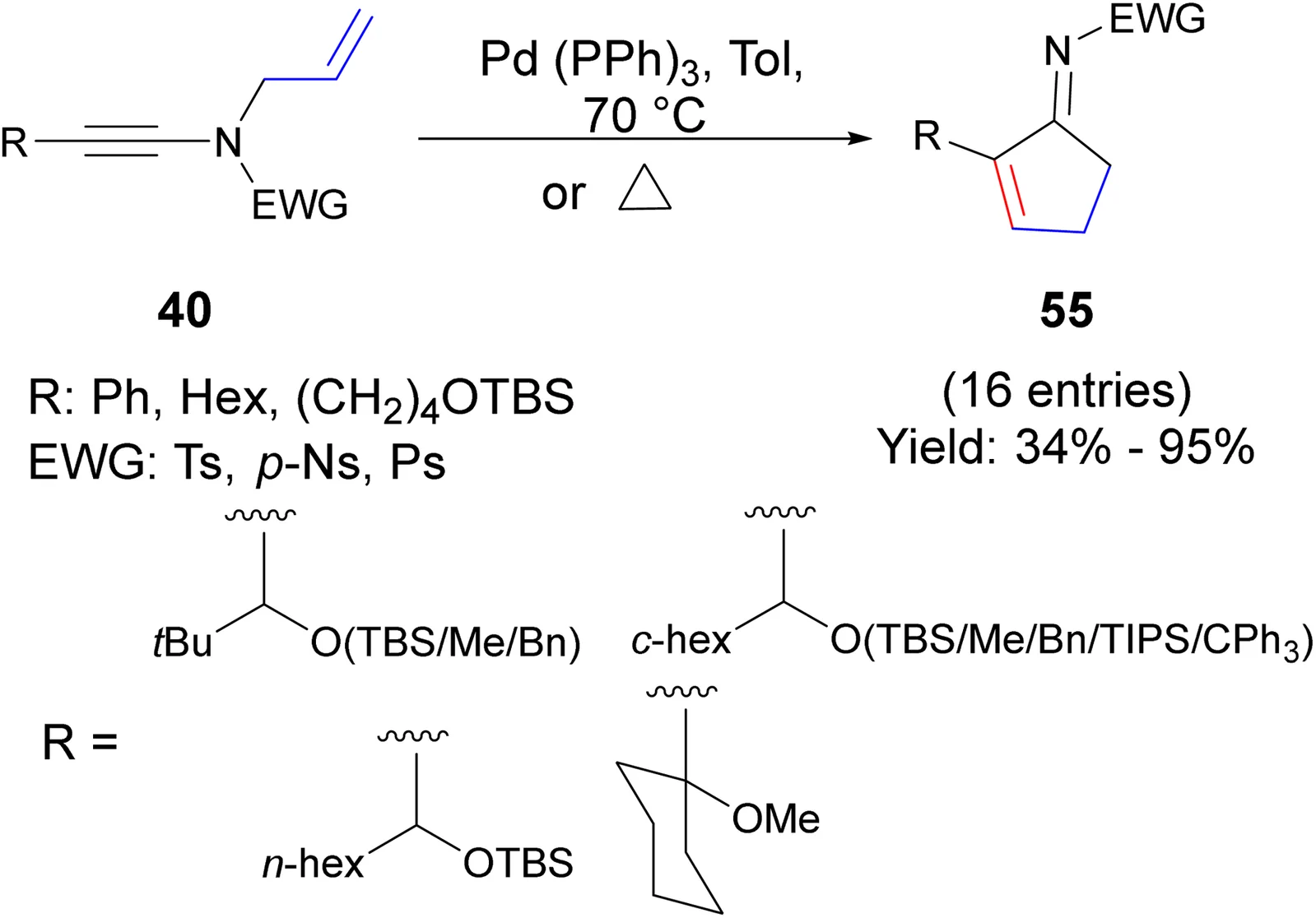
Aza-Claisen reaction followed by carbocyclization of *N*-allylynamides.

In continuation of this strategy, the same group in 2013 established a palladium-catalysed or thermal aza-Claisen rearrangement, followed by a carbocyclization reaction of *N*-allylynamides 40 to prepare a variety of α,β-unsaturated cyclopentenimines 55 in moderate to excellent yields (Scheme 19).^30^ The effect of electron-withdrawing groups and β-substituents of the ynamides on this tandem process was critically studied. The use of a palladium catalyst is mandatory for the synthesis of cyclopentenimines from *N*-sulfonyl ynamides as it proceeds through Pd–π-allyl complexes *via* a Rautenstrauch rearrangement. However, both Pd-catalysed and thermal conditions were proven to be successful for cyclopentenimine synthesis from *N*-phosphoryl ynamides *via* N-promoted 1,2-H shifts through zwitterionic intermediates, as shown in Scheme 15. *N*-Allyl-γ-branched *N*-sulfonyl and phosphoryl ynamides were smoothly reacted in the palladium-catalysed aza-Claisen-carbocyclization process to deliver the corresponding cyclopentenimines in good yields.

**Scheme 20:**
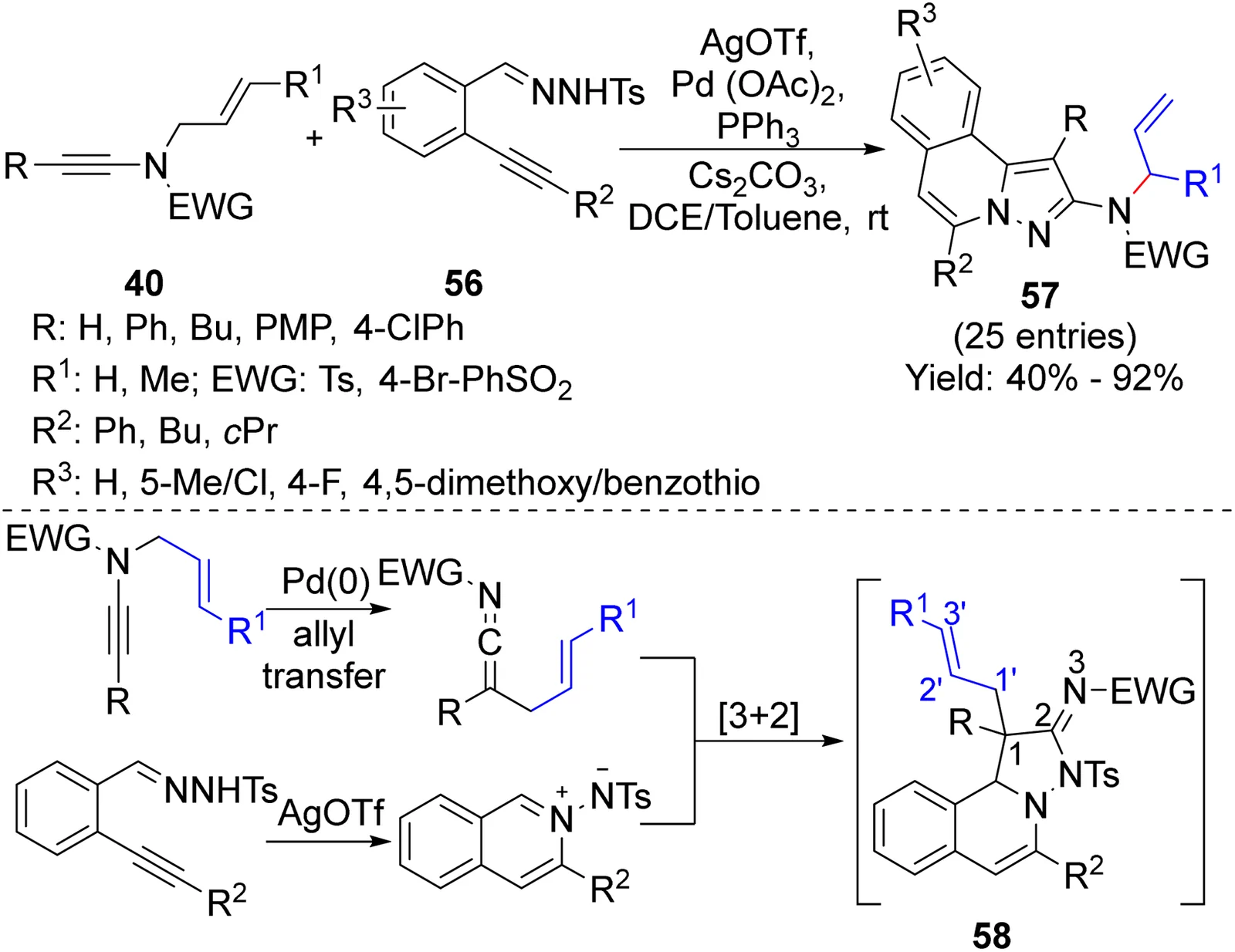
Multicatalytic reaction of *N*-allyl ynamide with *N*′-(2-alkynylbenzylidene) hydrazides.

Prompted by the advancements in the studies mentioned above, Peng and co-workers suggested a multicatalytic reaction of *N*-allyl ynamides 40 with *N*′-(2-alkynylbenzylidene)hydrazide 56 to obtain isoquinoline derivatives 57 in good to excellent yields (Scheme 20).^31^ To obtain the ideal reaction conditions, a range of Pd-catalysts, ligands, bases, and solvents were examined.

Subsequently, in the presence of Pd(OAc)_2_, PPh_3_ ligand, and Cs_2_CO_3_ as a base in DCE/toluene, the reaction culminated in an optimal yield. The reaction proceeded through the formation of a ketenimine intermediate *via* a ynamido–palladium–π-allyl complex, with subsequent N-to-C allyl migration. This intermediate underwent a [3 + 2] cycloaddition with isoquinolinium-2-yl amide (generated *via* silver-catalysed 6-*endo* cyclisation of hydrazide) to yield intermediate 58, which underwent a subsequent aza-Claisen rearrangement and aromatisation to afford the final adduct.

**Scheme 21:**
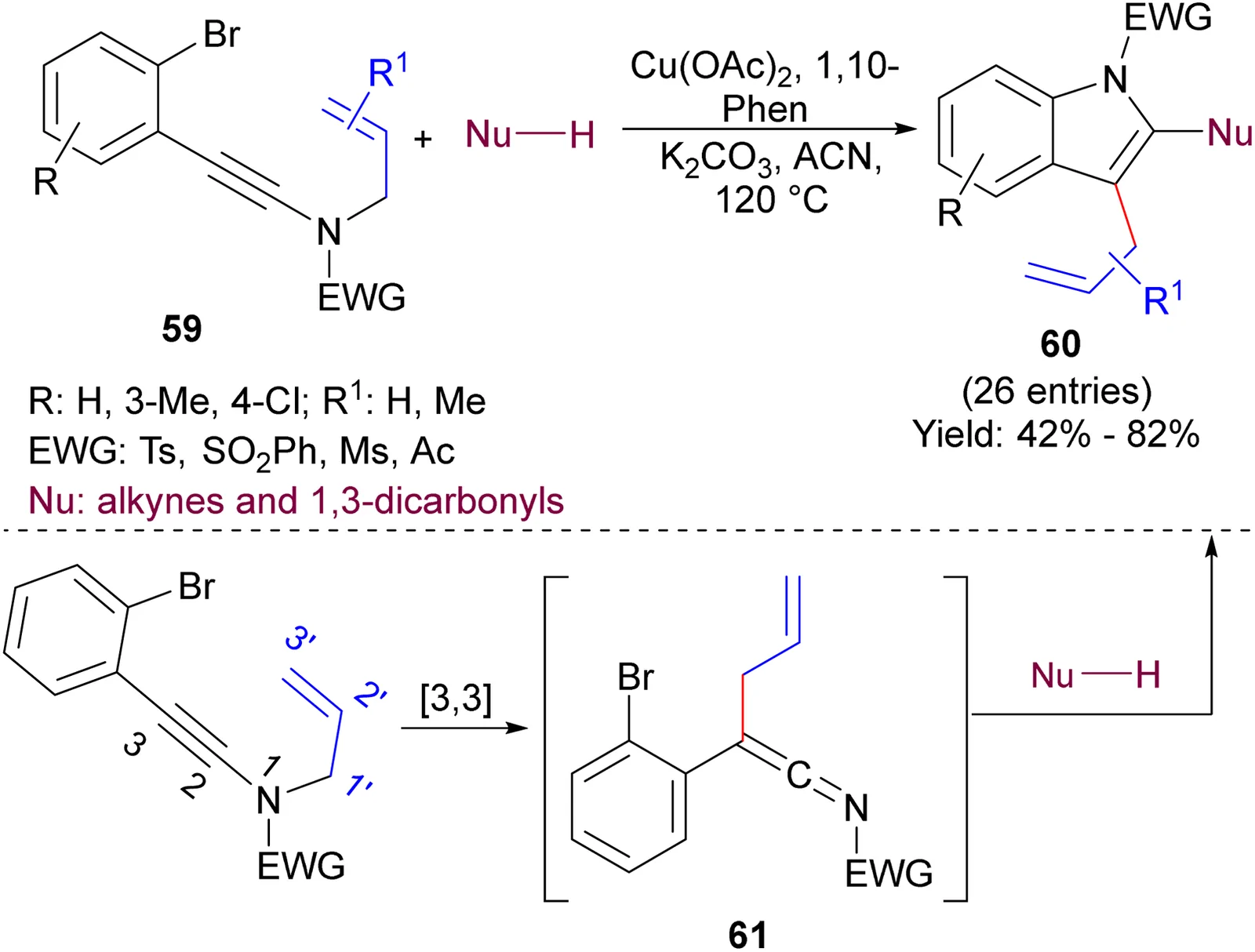
Synthesis of 2,3-disubstituted indoles using the Cu/Phen catalytic system.

In 2019, Yang, Li and co-workers^32^ accomplished a cascade annulation reaction strategy of *N*-allyl-*N*-((2-bromoaryl)ethynyl)amides 59 with terminal alkynes or 1,3-dicarbonyls for the synthesis of 2,3-disubstituted indoles 60 in the presence of a Cu/Phen catalytic environment (Scheme 21). This protocol starts with the generation of ketenimine intermediate 61 through a [3,3]-sigmatropic rearrangement, which further undergoes a series of reactions with terminal alkynes or 1,3-dicarbonyl compounds, *i.e.*, C–H functionalisation, Ullmann C–N coupling, and a cyclisation process to deliver indole derivatives in moderate to good yields. The methodology was well tolerated by various substituents on ynamides and nucleophiles. The resulting products 60 were effectively utilised in subsequent functionalisation processes in the synthesis of complex molecules.^32^

**Scheme 22:**
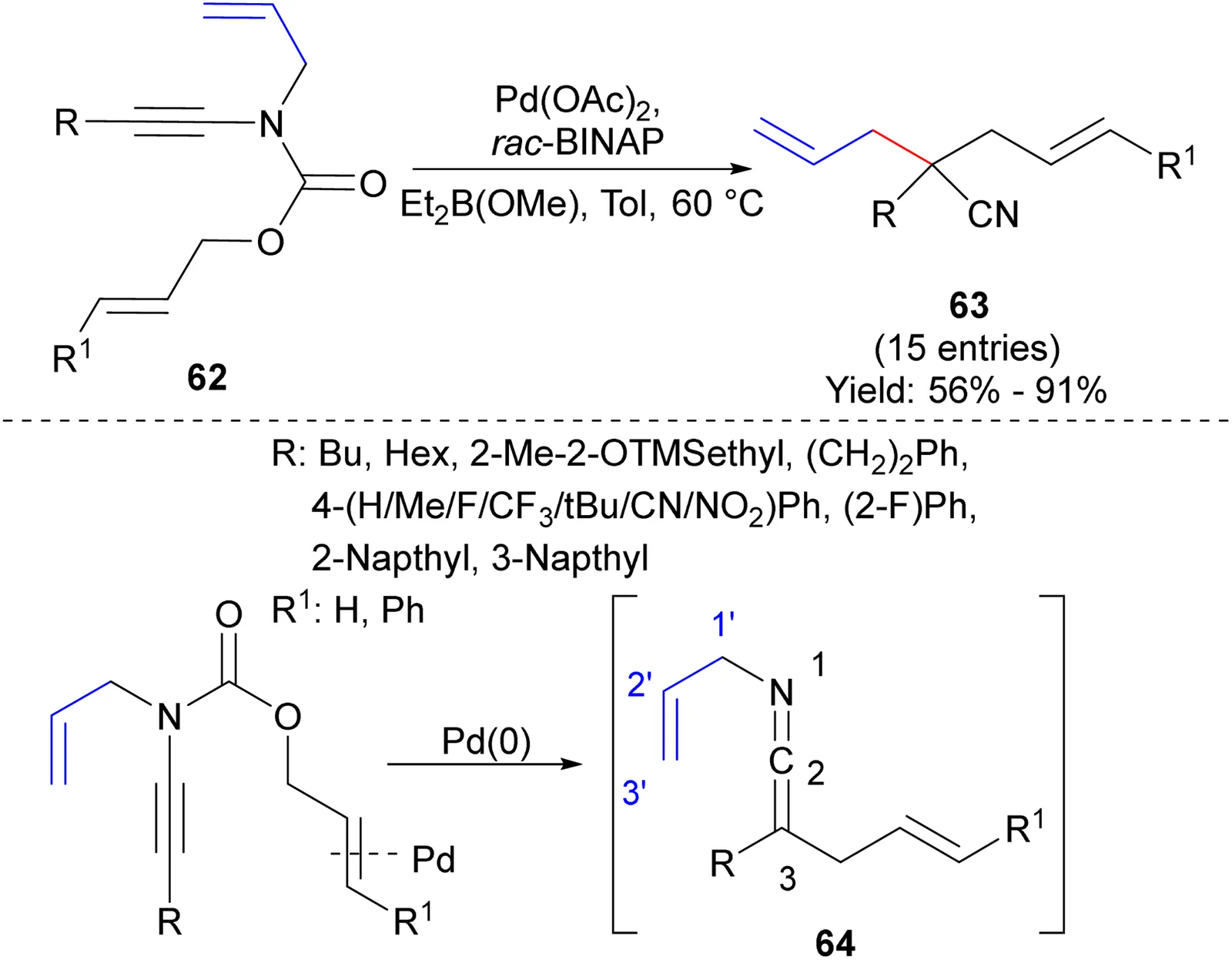
Synthesis of quaternary nitriles *via* aza-Claisen rearrangement.

In 2021, Cook and co-workers presented a Pd-catalysed auto tandem catalysis reaction of *N*-alloc-*N*-allyl ynamides 62 for the formation of quaternary nitriles 63 through an aza-Claisen rearrangement (Scheme 22).^33^ They varied the R group of the ynamide with alkyl and aryl groups having electron-donating and electron-withdrawing substituents to check the adaptability of the single Pd(0) catalyst in performing both π-allyl and aza-Claisen rearrangements simultaneously. However, all the substituents were well tolerated, resulting in the corresponding quaternary nitriles in high yields. The mechanistic pathway involves Pd(0)-catalysed π-allylic ionisation of the C–N bond, resulting in intermediate 64, followed by the aza-Claisen rearrangement, as demonstrated through a detailed computational analysis.

**Scheme 23:**
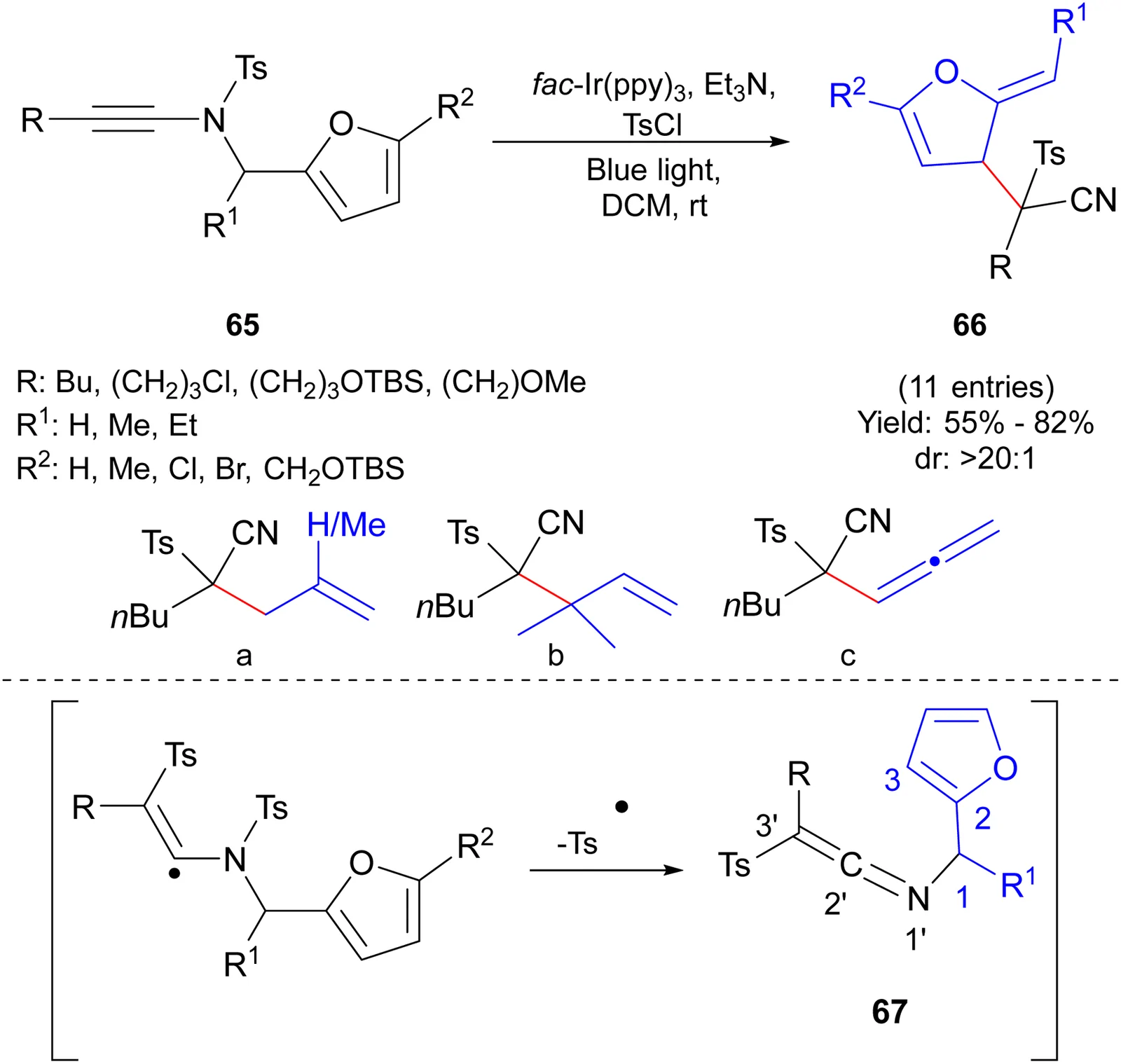
1,3-Sulfonyl shift-triggered aza-Claisen rearrangement of *N*-sulfonyl ynamides.

The first radical 1,3-sulfonyl shift-triggered aza-Claisen rearrangement of *N*-sulfonyl ynamides 65 was introduced by Yuan and Zhu in 2021, providing access to highly regio-, stereo-, chemo-, and diastereoselective functionalized quaternary nitriles 66 (Scheme 23).^34^ The *N*-sulfonyl ynamides having a furan ring at the allylic position to the N-atom, upon irradiation with blue LED light in the presence of an Ir-catalyst and TsCl, underwent a dearomatization reaction through an aza-Claisen rearrangement. This reaction resulted in α-allylated nitriles 66 in good yields and with greater diastereoselectivity. The reaction afforded mild reaction conditions and a broad substrate scope of nitriles, and varied types of substituents, such as Me, Br, Cl, and OTBS groups on the R group of ynamides, as well as on the furan ring, were well accommodated in the optimised reaction conditions. Various functionalized ketenimines were also isolated under the same reaction conditions with efficient yields.

The reaction pathway involved the irradiation of TsCl to generate a sulfonyl radical, which, upon regioselective addition to the ynamides, formed radical intermediate 67. Subsequent β-elimination regenerated the sulfonyl radical, allowing it to participate in the next process. The resulting ketenimine underwent an aza-Claisen rearrangement to give the final adduct.

**Scheme 24:**
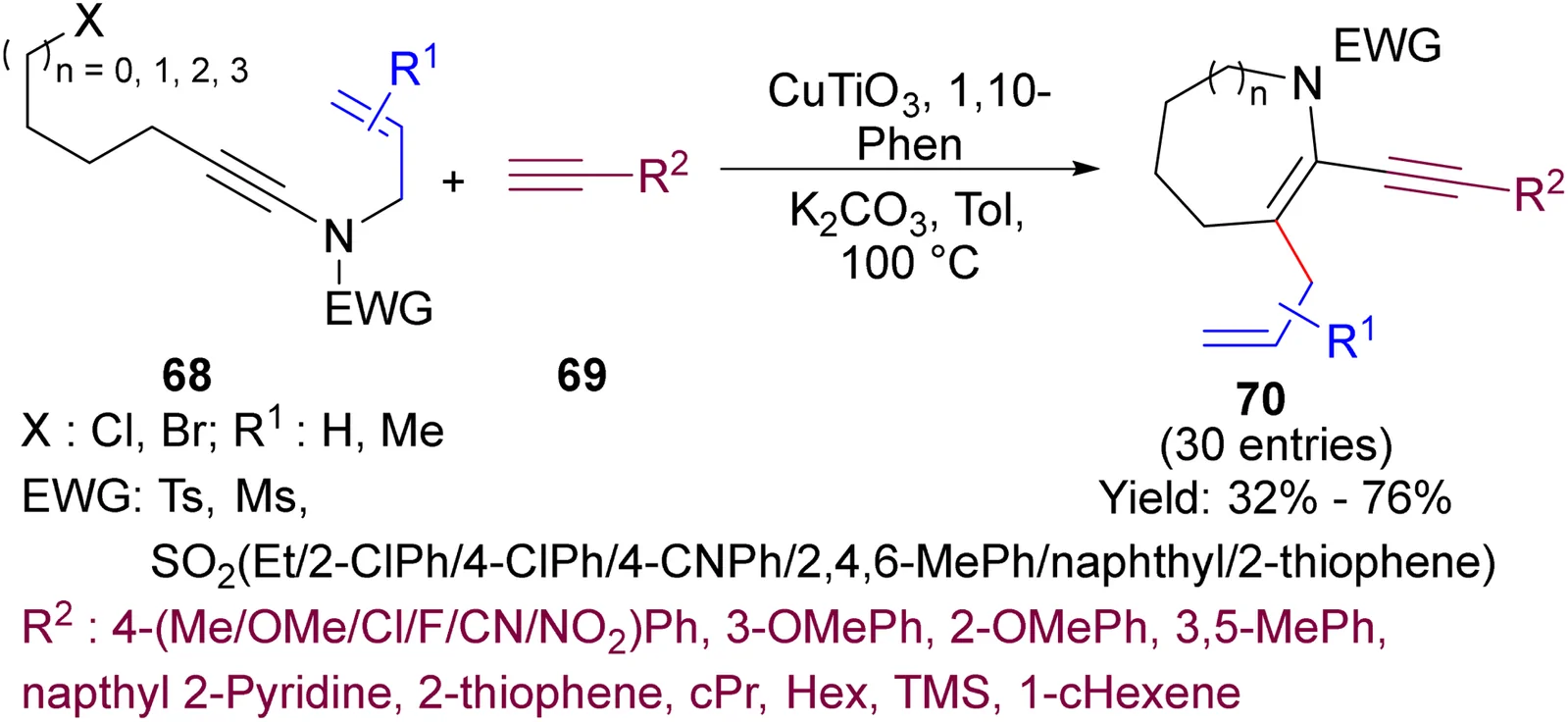
Synthesis of N-heterocyclic systems from ynamides and terminal alkynes.

In a related study by Yang and Li,^32^ in 2021, Wang and his group reported the synthesis of a diverse range of medium-sized N-heterocyclic 70 systems by the reaction of ynamides 68 with terminal alkynes 69 in the presence of a copper/Phen catalytic system (Scheme 24).^35^ The reaction proceeded in successive stages, including aza-Claisen rearrangement, C–H functionalization, C–N coupling, and cyclisation reactions. The first step in this reaction involves the formation of a ketenimine intermediate through the aza-Claisen rearrangement under heating conditions. The resulting ketenimine intermediate undergoes further C–H functionalization, C–N coupling, and cyclisation reactions to give the final product (mechanistic pathway, as shown in Scheme 21). A detailed DFT calculation was performed to establish the mechanistic pathway of this reaction.

**Scheme 25:**
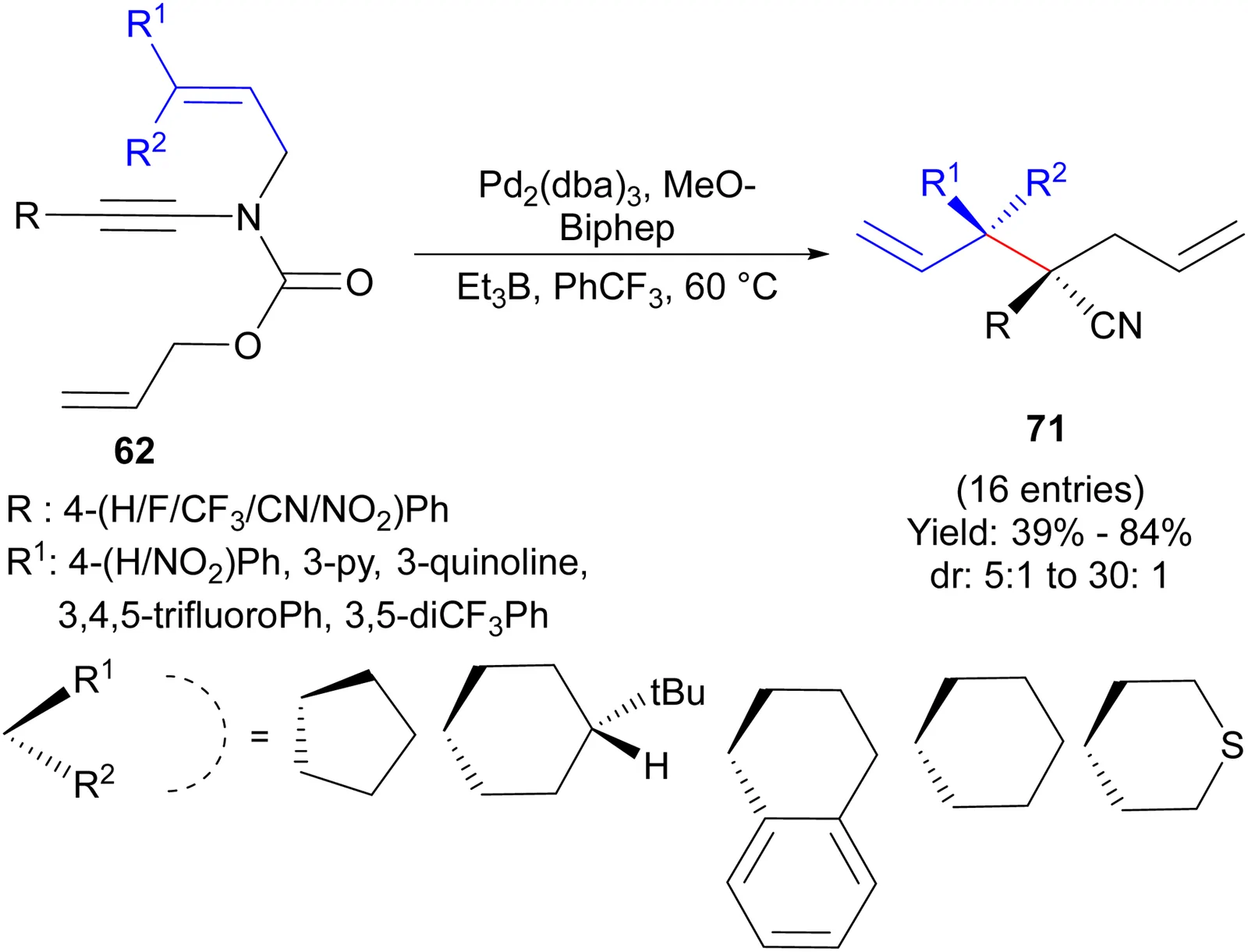
Branched selective aza-Claisen rearrangement of *N*-alloc-*N*-allyl ynamides.

Gaining insights into the Pd(0) catalysed auto tandem catalysis reaction from their previous work,^33^ Cook's group introduced a highly diastereoselective synthesis of quaternary nitriles 71 containing two contiguous all-carbon quaternary centres from the branched selective aza-Claisen rearrangement of substituted *N*-alloc-*N*-allyl ynamides 62 (Scheme 25).^36^ A wide variety of ynamides with mono- and di-substitutions on the γ-carbon were effectively used to produce the desired products in excellent yields and diastereoselectivities. The Ph, 4-NO_2_-Ph, 4-CF_3_-Ph, 4-F-Ph, and 4-CN-Ph substituents on the R group of ynamides were well suited to these auto-tandem catalysis reaction conditions. When ynamides with *E*- and *Z*-carbamates were used, two diastereomers were produced with higher stereospecificity and comparable chemical efficiency. Computational analysis confirms the molecular pathway, which comprises two allylic rearrangements (the π-allyl and aza-Claisen rearrangements) catalysed by Pd(0), resulting in two vicinal stereocentres. The final adducts were successfully utilised in diaryl allyl substitution and spirocyclic nitrile formation.

**Scheme 26:**
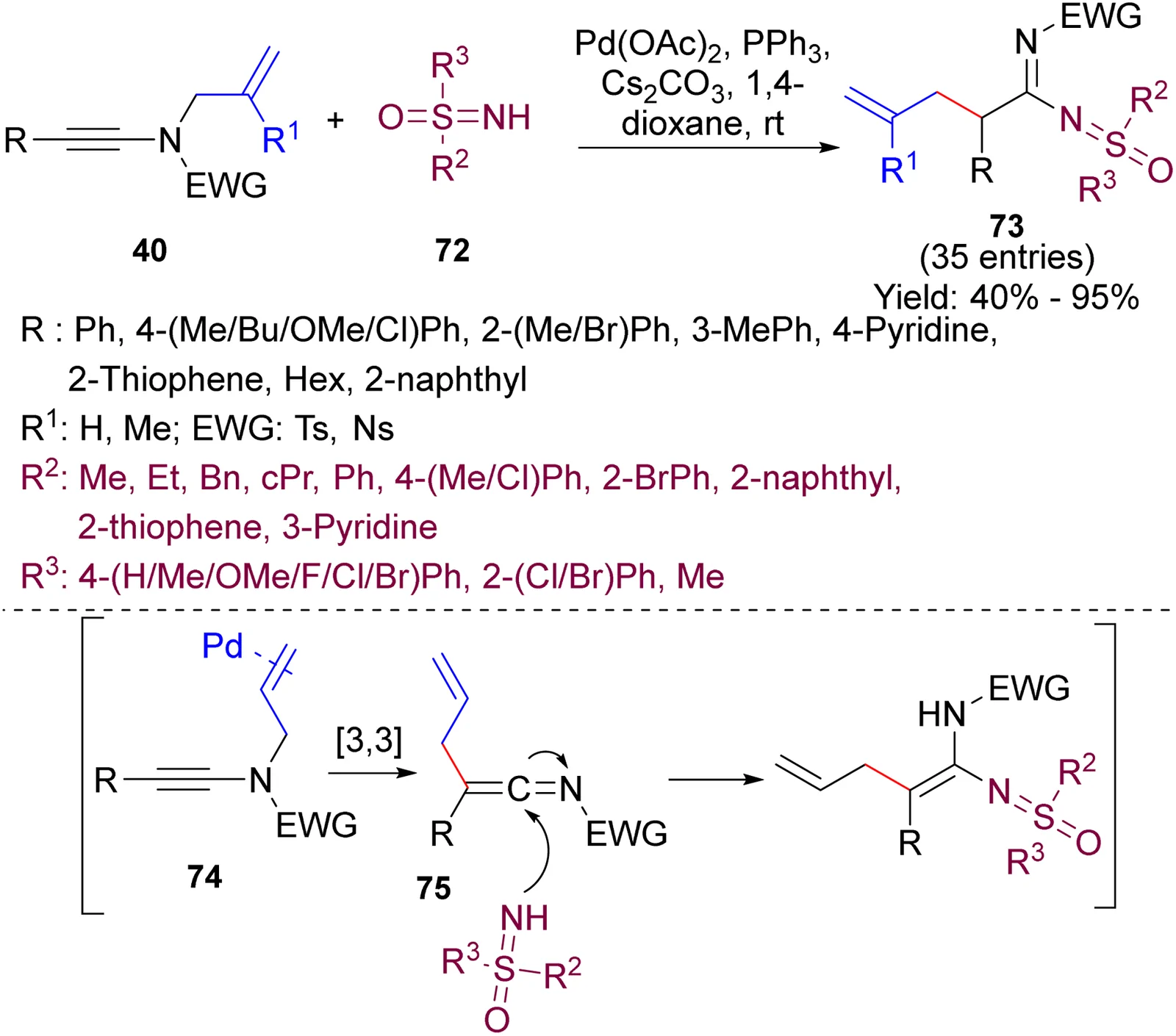
Pd-catalysed cascade aza-Claisen rearrangement of *N*-allyl ynamides.

Very recently, Chen and co-workers reported a Pd-catalysed cascade aza-Claisen rearrangement reaction of *N*-allyl ynamide 40, followed by the nucleophilic addition of NH-sulfoximines 72, resulting in sulfoximidoyl amidines 73 in good to excellent yields (Scheme 26).^37^ Under these mild and atom-economic reaction conditions, a wide variety of functional groups on the imines and ynamides were well tolerated. To maximise the yield, a variety of Pd catalysts, ligands, and bases were used in the reaction. In addition to Pd catalysts, Lewis acid catalysts, including Cu(OTf)_2_, AgOTf, and Rh_2_(OAc)_4_, did not produce any of the intended products. In addition, *N*-allyl alkynyl ynamides with both electron-withdrawing and electron-donating substituents smoothly reacted to produce the corresponding sulfoximidoyl amidines in impressive yields. The reaction involves a sequence of steps: π-allyl–Pd complex formation (74, Scheme 26), [3,3]-sigmatropic rearrangement reaction, and subsequent nucleophilic addition reaction to obtain the target molecule.

#### 2.1.4. Thio-Claisen rearrangement/sulfonium rearrangement

The first example of the thio-Claisen rearrangement (TCR) was reported in 1962, which is the sulfur equivalent of the conventional Claisen rearrangement reaction.^38^ This involves the [3,3]-sigmatropic rearrangement of allyl vinyl sulfide, furnishing a γ,δ-unsaturated thiocarbonyl compound under mild conditions, which has great importance as a molecular backbone in natural product synthesis. Although the mechanistic aspects of the TCR reaction are similar to those of the oxy-Claisen rearrangement, only a few examples of TCR reactions of ynamides have been described. Herein, we have attempted to address recent findings on the TCR reaction of ynamides.

**Scheme 27:**
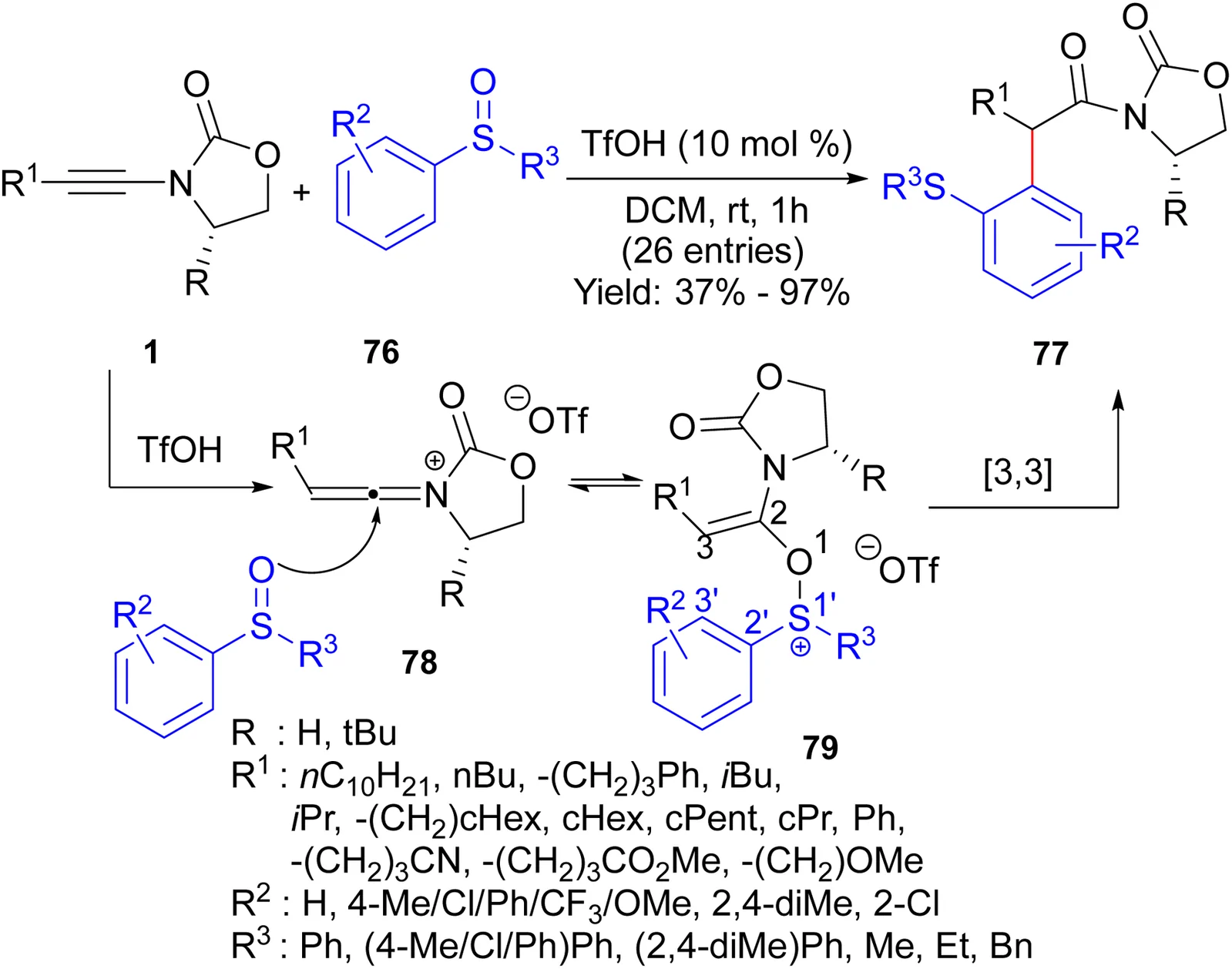
TfOH-catalysed sulfonium rearrangement of ynamides.

Maulide and co-workers in 2014 developed a metal-free TfOH-catalysed redox arylation of ynamides 1 with disubstituted sulfoxides 76 as arylating agents (Scheme 27).^39^ A broad range of ynamides and aryl sulfoxides with electron-withdrawing and -donating substituents were well tolerated to afford α-arylated acyl oxazolidinones in good to excellent yields under mild reaction conditions. Various deuterium labelling control experiments were conducted to investigate the mechanistic cycle, which concluded that the final adduct was formed through a tandem sulfoxide addition to the *in situ*-formed keteniminium 78, resulting in intermediate 79, followed by a Claisen rearrangement and rearomatization process. The asymmetric version of this redox arylation reaction was also established using a chiral auxiliary containing a ynamide, which led to the formation of the corresponding chiral α-arylated acyl oxazolidinone in 92% yield, with good diastereoselectivity (dr = 75 : 25). However, allylic and propargylic alcohols failed to act as aryl donors under the optimised reaction conditions.

**Scheme 28:**
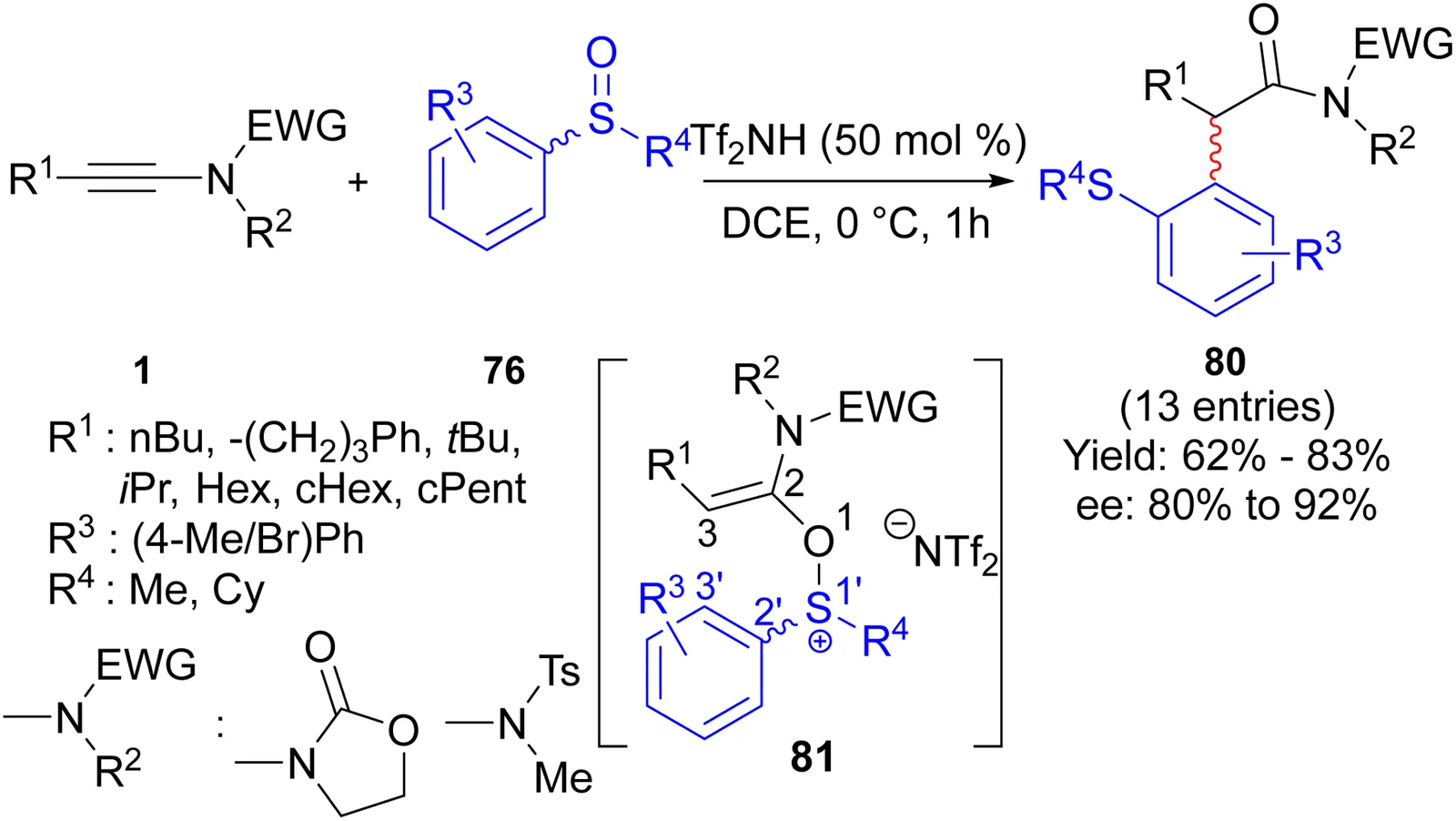
Tf_2_NH-catalysed sulfonium rearrangement of ynamides.

The same group further studied a similar strategy to establish a very rare 1,4-chirality transfer from sulfur to a carbon atom using chiral sulfoxides 76 in the presence of trifluromethanesulfonimide, Tf_2_NH, through a sulfonium [3,3]-sigmatropic rearrangement (Scheme 28).^40^ This protocol provides efficient access to α-arylated amides with yields ranging from good to high and an enantiomeric ratio of up to 99.5 : 0.5 under mild reaction conditions. Under the set experimental conditions, a range of cyclic and acyclic aryl alkyl sulfoxides were examined; nevertheless, it was found that the cyclic sulfoxides and oxazolidinone-based ynamides with bulky groups at the C-terminus produced the best enantioselectivity. Quantum chemical calculations provided insights into the mechanism of chirality transfer through intermediate 81 and the dependence of excellent enantioselectivity on the catalyst and substrate used.

**Scheme 29:**
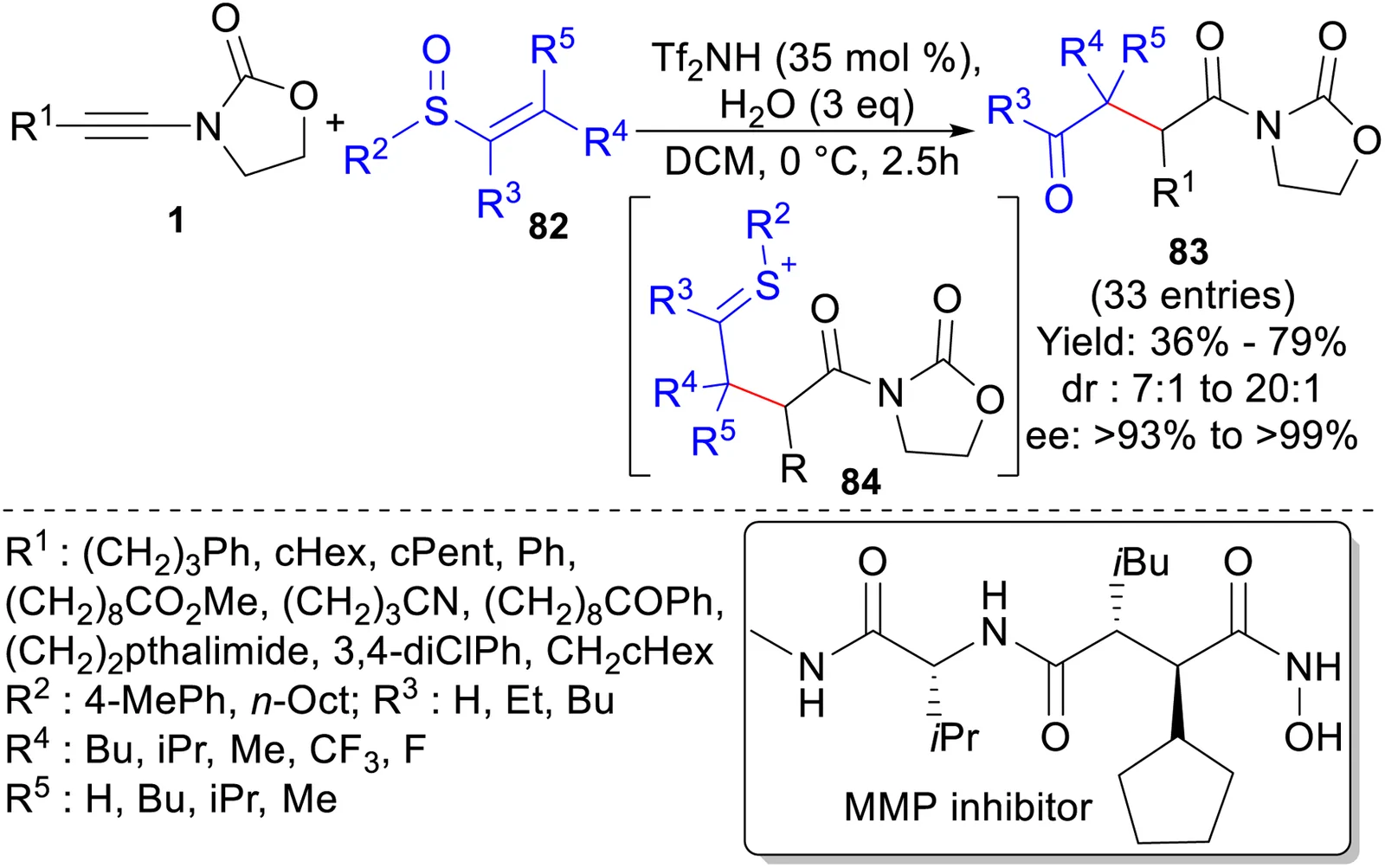
Synthesis of 1,4-dicarbonyls *via* sulfonium rearrangement of ynamides.

Concurrently, the above sulfonium [3,3]-sigmatropic rearrangement chemistry was further extended to a Brønsted acid-catalysed traceless stereodivergent synthesis of acyclic, polysubstituted 1,4-dicarbonyls from the reaction of chiral vinylsulfoxides and ynamides (Scheme 29).^41^ The absolute configuration was controlled by the chirality at the sulfur atom, whereas the *E*/*Z* geometry of the vinylsulfoxide double bond determines the relative stereochemistry in the final products 83. (*E*)-Vinylsulfoxides resulted in the synthesis of *syn*-dicarbonyls, while (*Z*)-vinylsulfoxides generated *anti*-dicarbonyls through a Zimmerman–Traxler-like transition state. Chiral sulfoxide regulated the stereochemistry of the final product; however, the application of chiral ynamide produced diastereomeric compounds with dr values of 10 : 1 and 3 : 1, highlighting the importance of matched and mismatched double stereodifferentiation. This strategy provides a wide range of substrate scope for the highly diastereoselective and excellent enantioselective formation of 1,4-dicarbonyl compounds with vicinal stereocentres in an atom-economical path. The mechanistic cycle follows the same pathway as elucidated in Scheme 27 to generate intermediate 84, which on treatment with H_2_O resulted in the desired product. The final product was converted to an important precursor of the MMP inhibitor *via* aldehyde oxidation, followed by amide coupling.

**Scheme 30:**
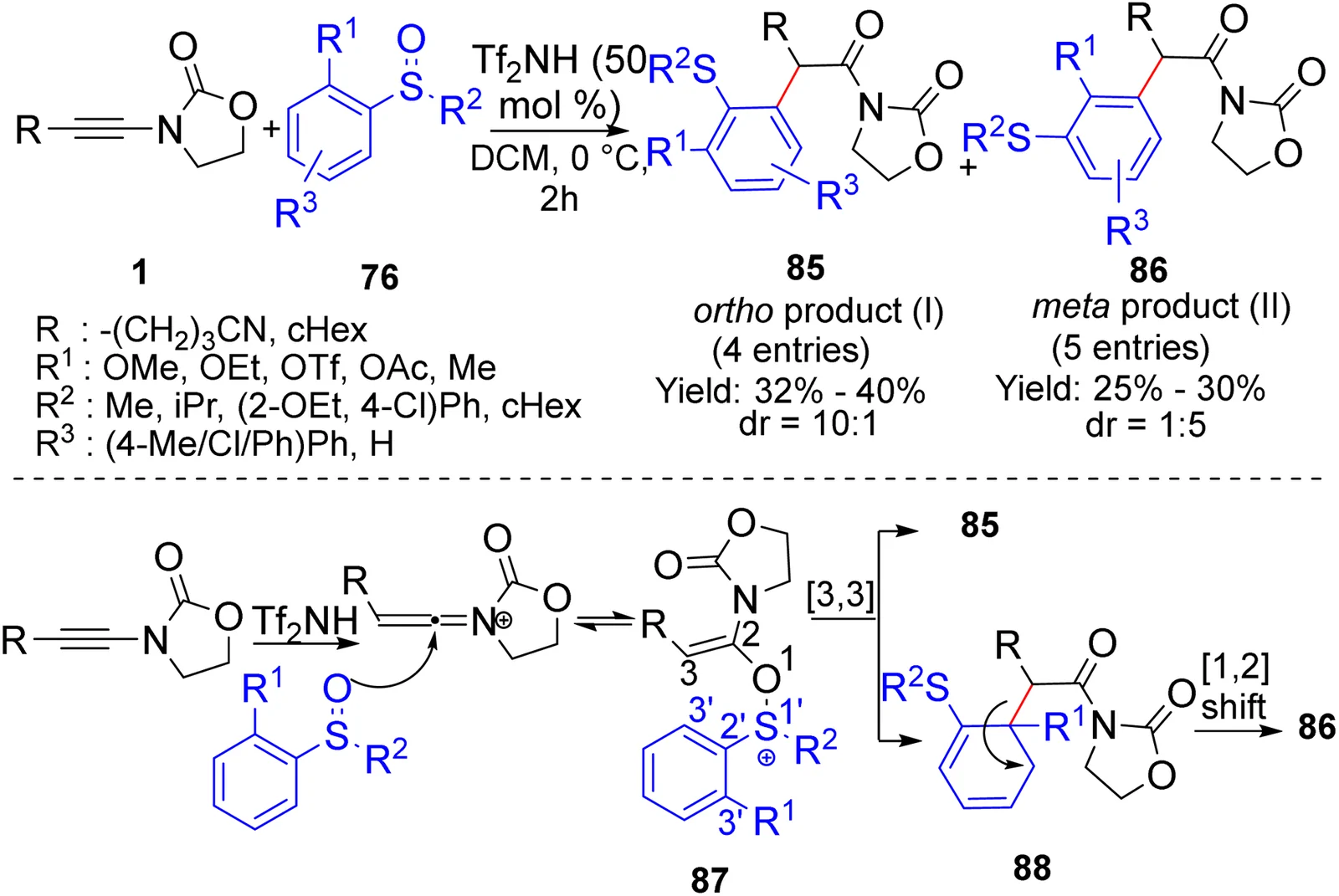
Synthesis of *meta*-selective α-arylamides *via* sulfonium rearrangement of ynamides.

The Maulide group developed an unusual Brønsted acid-catalysed ynamide oxoarylation for the synthesis of *meta*-selective α-arylamides in moderate yields but with high stereocontrol *via* sulfonium [3,3]-Claisen rearrangement (Scheme 30).^42^ In the presence of the Tf_2_NH catalyst, the treatment of ynamide 1 with chiral sulfoxide 76 afforded two diastereomers, 85 and 86. This was explained by two competing reaction routes. According to the ESI-MS and computational studies carried out by the authors, product 85 is formed *via* the generation of intermediate 87, followed by a [3,3]-sigmatropic rearrangement and rearomatization process, whereas product 86 is formed *via* the generation of intermediate 88 (from the [3,3]-sigmatropic rearrangement of 87), which experiences a [1,2] shift and then deprotonation to regenerate aromaticity.

**Scheme 31:**
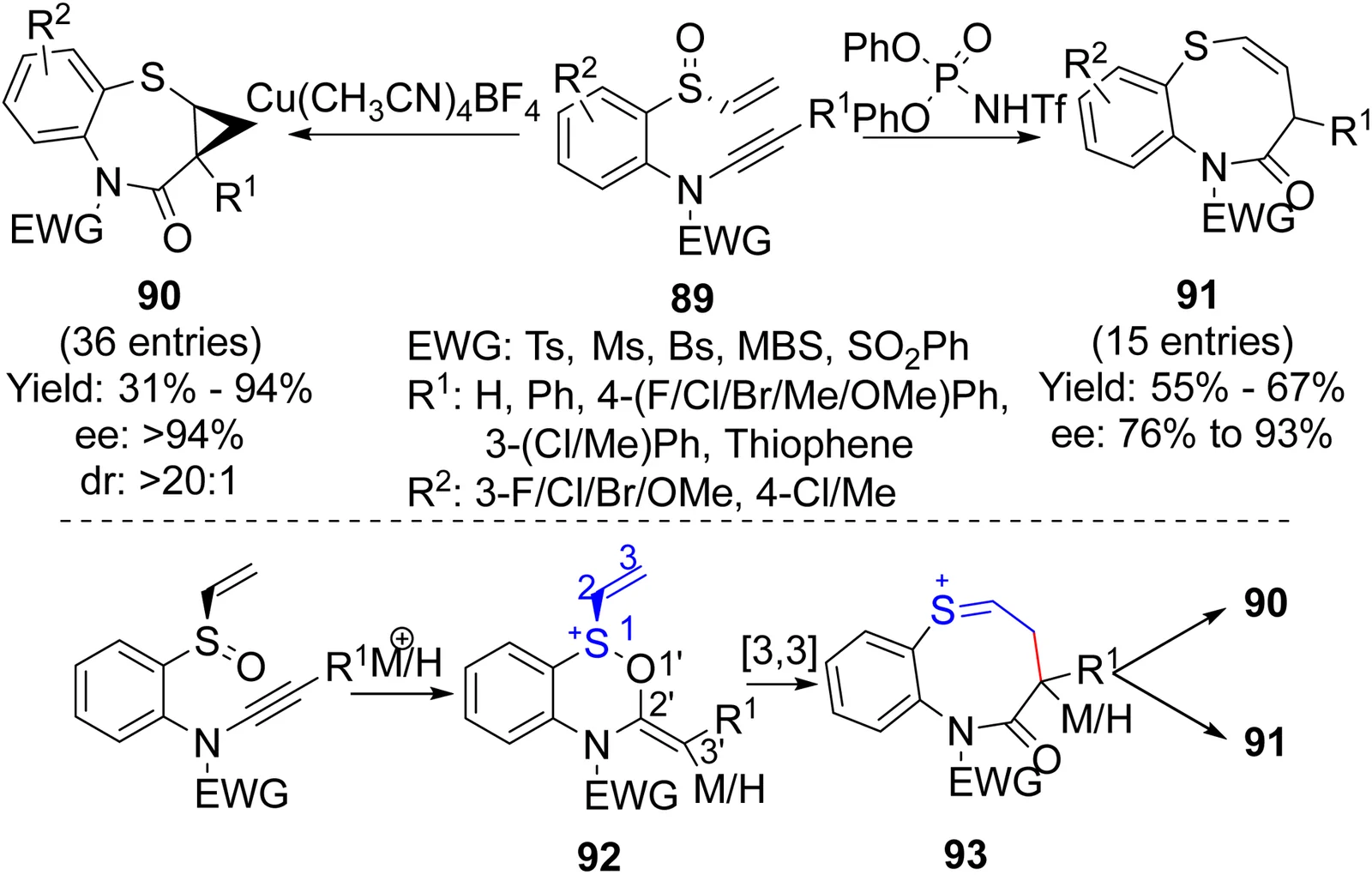
Catalyst-dependent intramolecular sulfonium rearrangement of ynamides.

Ye and co-workers prepared biologically active chiral medium-sized N,S-heterocycles *via* a catalyst-dependent intramolecular [3,3]-sigmatropic rearrangement of sulfoxide-ynamides under mild conditions (Scheme 31).^43^ The ynamides containing a vinyl sulfoxide group, on treatment with transition metal catalysis (Cu-based catalyst), resulted in the formation of 1,5-benzothiazepines 90, while in the presence of a Brønsted acid catalyst (phosphoramide catalyst), 1,6-benzothiazocines 91 were produced as the final adduct. This strategy delivers atom-economic, stereospecific, and divergent synthesis of products with a wide substrate scope in moderate to good yields. The asymmetric version of these medium-sized N,S-heterocycles has been accomplished in the presence of chiral metal catalysts or chiral Brønsted acid catalysis with exceptional enantioselectivities *via* a chirality-transfer approach. Mechanistically, the ynamide is activated either by a metal or acid catalyst, followed by *in situ* addition of the oxygen atom of the sulphoxide to generate intermediate 92. This intermediate undergoes a subsequent [3,3]-sigmatropic rearrangement to deliver intermediate 93. Upon demetallation and deprotonation of intermediate 93, two products 90 and 91 were produced, respectively.

**Scheme 32:**
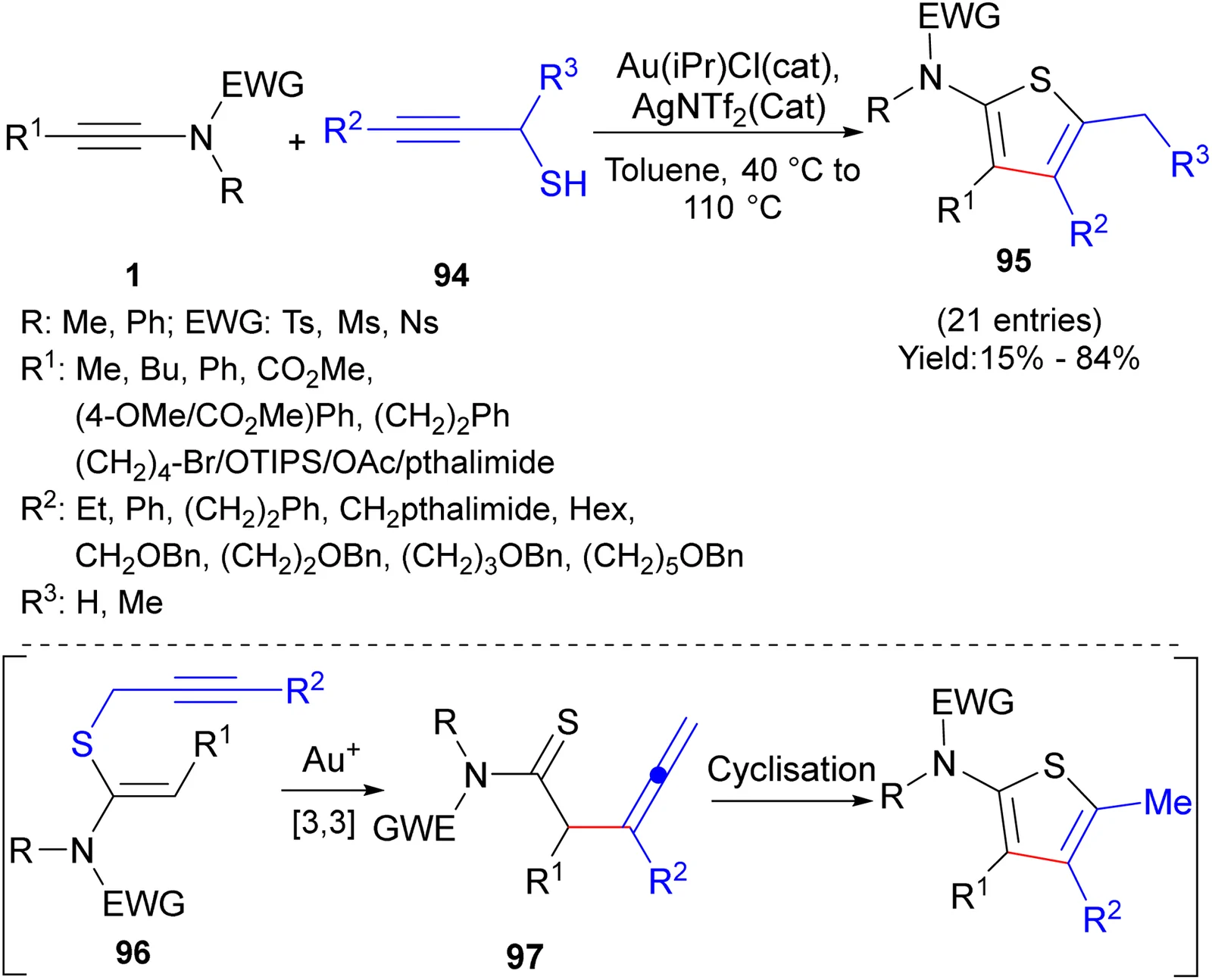
Au(i)-catalysed cascade thio-Claisen rearrangement and cyclisation of ynamides.

Inspired by their previous studies on ynamide chemistry, the Sato group in 2024 presented an Au(i)-catalysed cascade reaction sequence of ynamides and propargylic thiols to produce multisubstituted 2-aminothiophenes 95 in moderate to good yields (Scheme 32).^44^ This cascade reaction involves regioselective hydrothiolation of propargylic thiol 94 with ynamide 1, followed by thio-Claisen rearrangement, resulting in 3,4-dienamide 97*in situ*, which undergoes cyclisation to give the desired product 95. In the presence of Au(IPr)Cl (5 mol%) and AgNTf_2_ (5 mol%) catalysts, a wide range of substrates were converted to 2-aminothiophene derivatives in a single step without the formation of any intermediate. The authors also isolated 3,4-dienamide 97 in a good yield.

#### 2.1.5. Saucy–Marbet rearrangement

Allenes are frequently found in biologically active natural products and are also useful intermediates in chemical synthesis.^45^ The Claisen rearrangement of propargyl vinyl ethers, *i.e.*, the Saucy–Marbet rearrangement, is one of the straightforward and efficient synthetic methods for creating complex functionalized allenes. Moreover, the involvement of ynamides in this reaction methodology resulted in the formation of homoallenyl amides, which could play a significant role in the synthesis of multifunctional scaffolds. The accomplishments in the field of Claisen rearrangement of ynamides with propargyl alcohols are covered in this section.

**Scheme 33:**
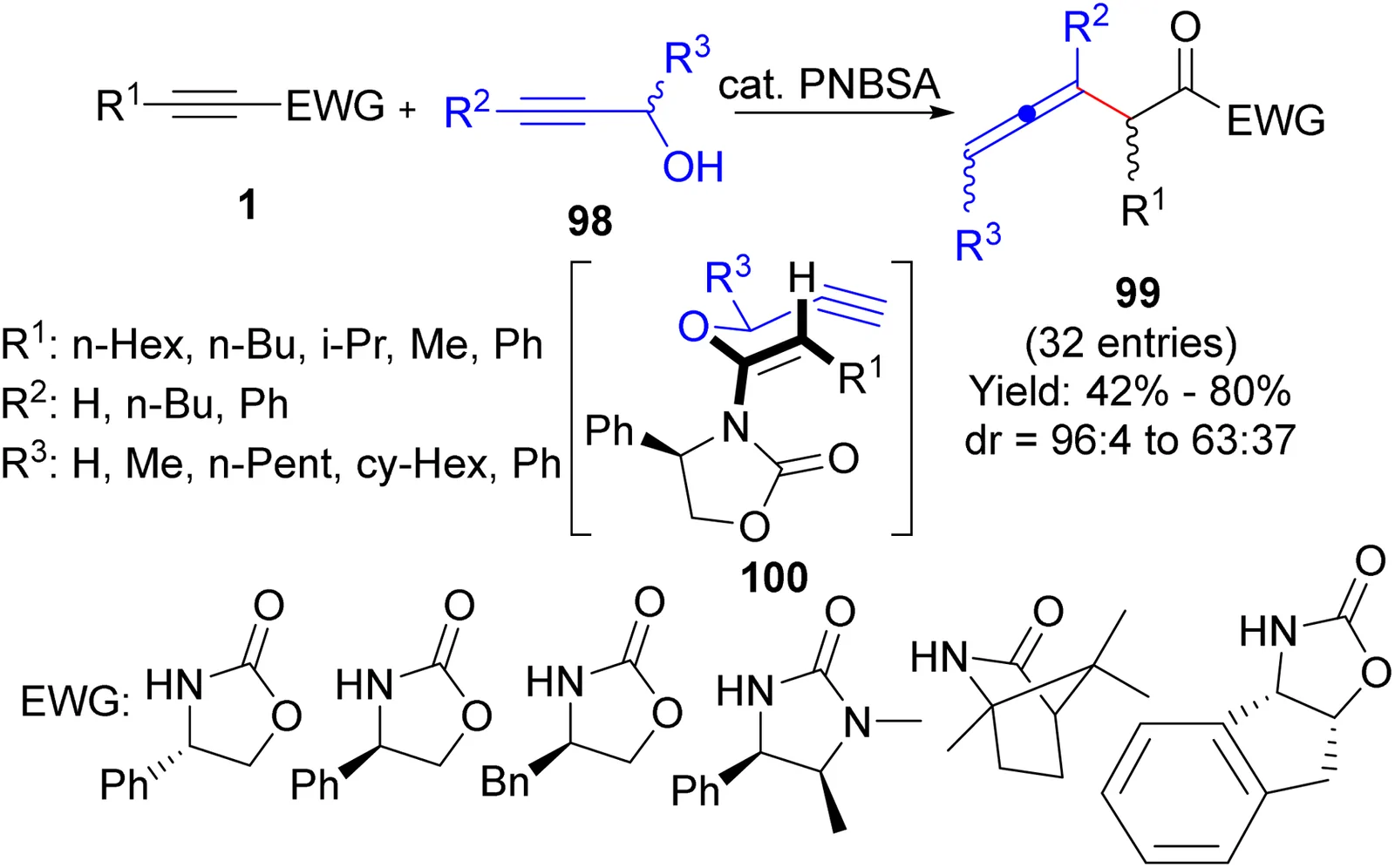
PNBSA-catalysed Saucy–Marbet rearrangement of ynamides.

The first stereoselective Brønsted acid-catalysed Saucy–Marbet rearrangement of chiral oxazolidinone-derived ynamides 1 and propargyl alcohols 98 was reported by Hsung *et al.* in 2003 (Scheme 33).^46*a*^ This rearrangement was catalysed by *para*-nitrobenzenesulfonic acid (PNSBA), leading to the diastereoselective synthesis of highly substituted homoallenyl amides 99. In the presence of 10–20 mol% of the PNBSA catalyst, both the (*R*)/(*S*)-oxazolidinone-substituted ynamides and (*R*)/(*S*)-propargyl alcohols were well tolerated, yielding the final product in moderate to good yields. The authors found that the incorporation of propargyl alcohol with ketene aminal moiety 100 from the *syn*-selective side favored the formation of the final adduct through a chair conformation. The resulting products were further reduced with LiBH_4_ to give chiral homoallenyl alcohols in good yields. In 2006, the same group demonstrated a detailed study on the mechanistic model and the effect of various substituents on the substrates using chiral ynamides and chiral propargyl alcohols under the same reaction conditions.^46*b*^

**Scheme 34:**
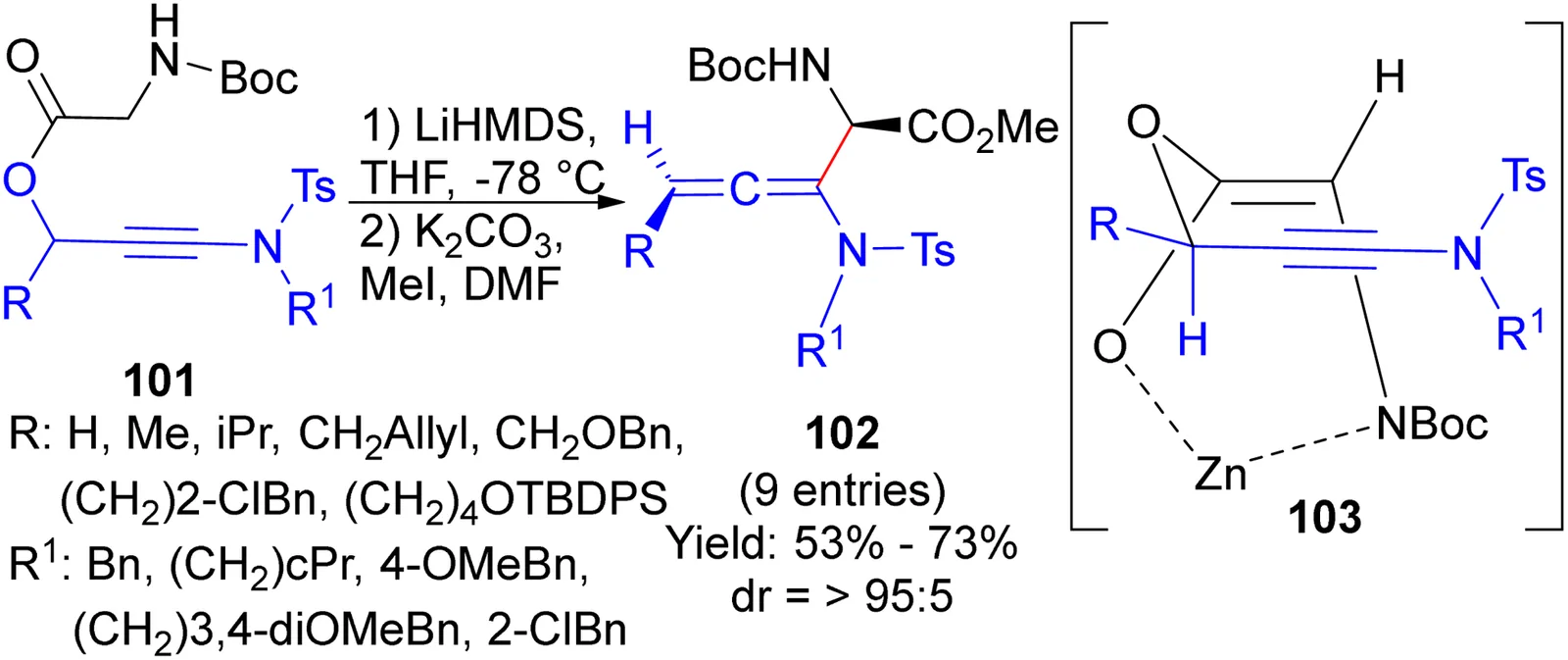
Synthesis of functionalized allenamides *via* the Saucy–Marbet rearrangement of *N*-Boc glycinates.

In 2013, an attractive strategy incorporating *N*-Boc glycinates (derived from propargyl ynamido–alcohols) as substrates was described by Cossy and co-workers to afford highly functionalized allenamides *via* stereoselective Claisen rearrangement (Scheme 34).^47^ The starting material reacts with LiHMDS (THF, −78 °C), followed by treatment with ZnCl_2_, generating chelated α-amino zinc enolate 103, which undergoes a subsequent enolate Claisen rearrangement reaction to furnish the corresponding carboxylic acid. Then, the desired allenamides 102 resulted in a 53–73% yield on treatment with (MeI, K_2_CO_3_, DMF, and rt). Functionalized allenamides were produced in good yields by varying the substituents at the nitrogen atom and the propargylic position. The resulting allenamides were then subjected to silver-catalysed cyclisation to give 3-pyrrolines in excellent yields and high diastereoselectivity, which are valuable precursors for the generation of substituted pyrrolidines.

**Scheme 35:**
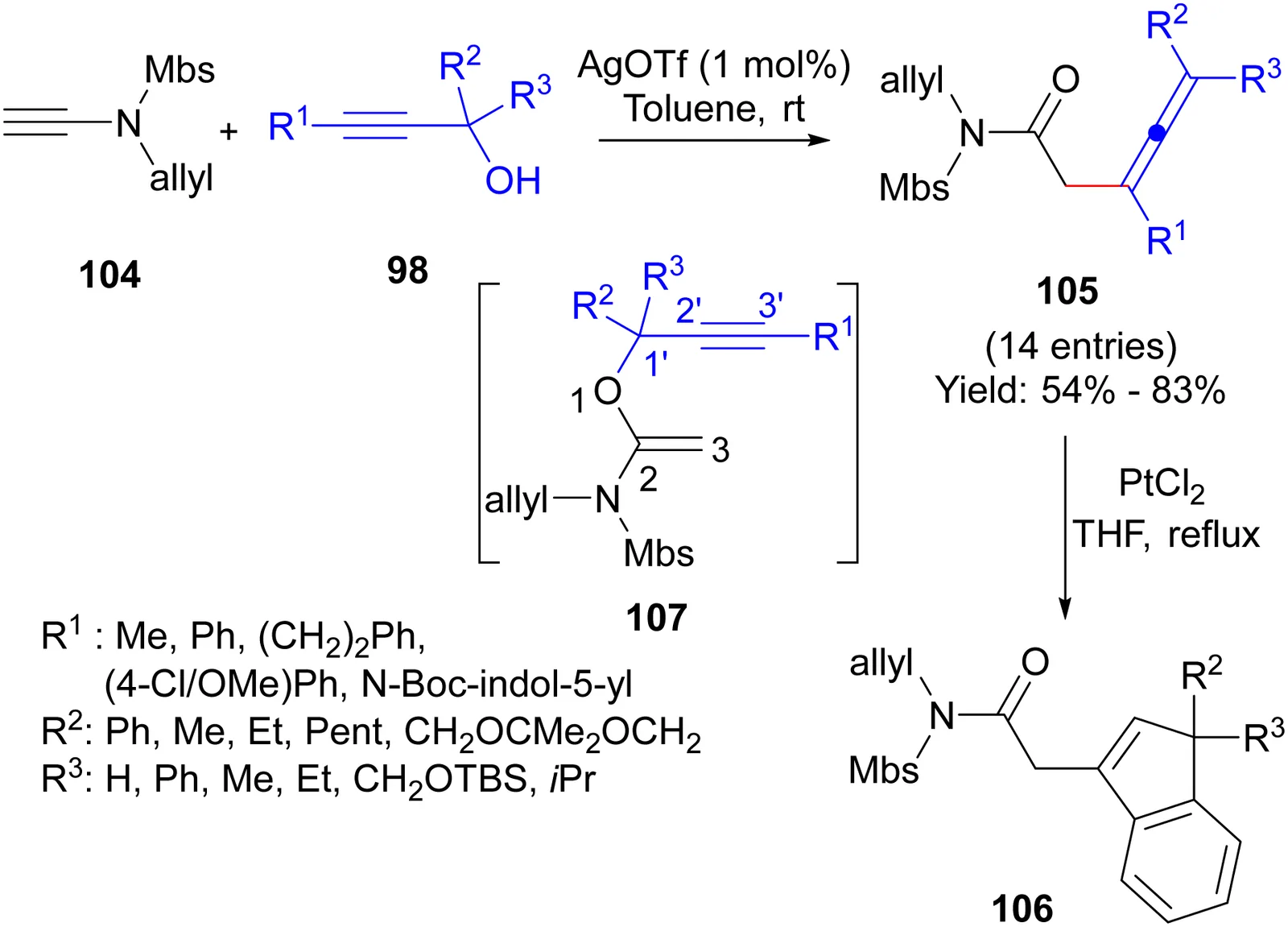
Au-catalysed Saucy–Marbet rearrangement of terminal ynamides.

In 2014, Egi and co-workers demonstrated a silver-catalysed transformation of terminal ynamides 104 and tertiary propargyl alcohols 98 to tetra-substituted allenes 105 and further to highly substituted indenes through a central-axial-central chirality transfer process (Scheme 35).^48^ They varied different substitutions on the ynamides and propargyl alcohols to establish the versatility of their methodology, and surprisingly, all the substrates smoothly reacted under their optimised reaction conditions.

The reaction involved *in situ* formation of propargyl vinyl ether intermediate 107 through the hydroalkoxylation of the alcohol on the vinyl ynamido–silver complex, which underwent a [3,3]-sigmatropic rearrangement reaction to furnish product allenes 105 with good yield and selectivity. The resulting allenes were then transformed into highly substituted indenes 106, bearing a quaternary stereocentre, through a Pt-catalysed intramolecular cyclisation process.

**Scheme 36:**
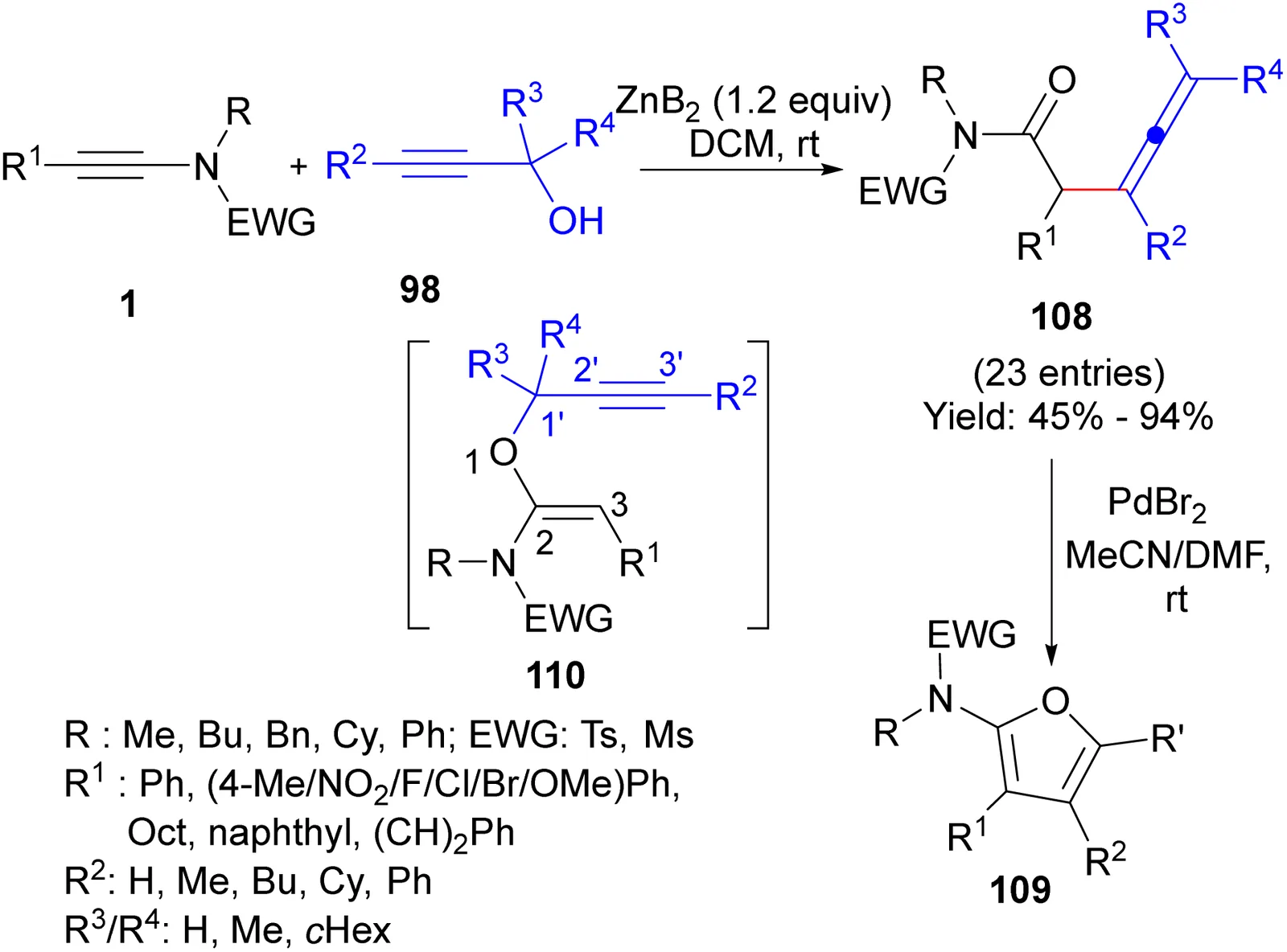
Synthesis of homoallenyl amides *via* the Saucy–Marbet rearrangement of ynamides.

Concurrently, Zhu and co-workers developed a simple and efficient Zn-catalysed reaction of ynamides 1 and propargyl alcohols 98 in 2015 for the synthesis of substituted homoallenyl amides 108, with good to excellent yields (Scheme 36).^49^ Under their optimised reaction conditions, they used a stoichiometric amount of ZnBr_2_ as a catalyst for the rearrangement process. All types of substituents were well tolerated in this methodology; however, the terminal ynamide failed to produce the desired product under the optimised reaction conditions. The *in situ* generated propargylic vinyl ether serves as the key intermediate 110 in this process. The synthesised homoallenyl amides underwent a Pd-catalysed intramolecular cyclisation reaction to form tetra-substituted furans 109 in 51–95% yields.

**Scheme 37:**
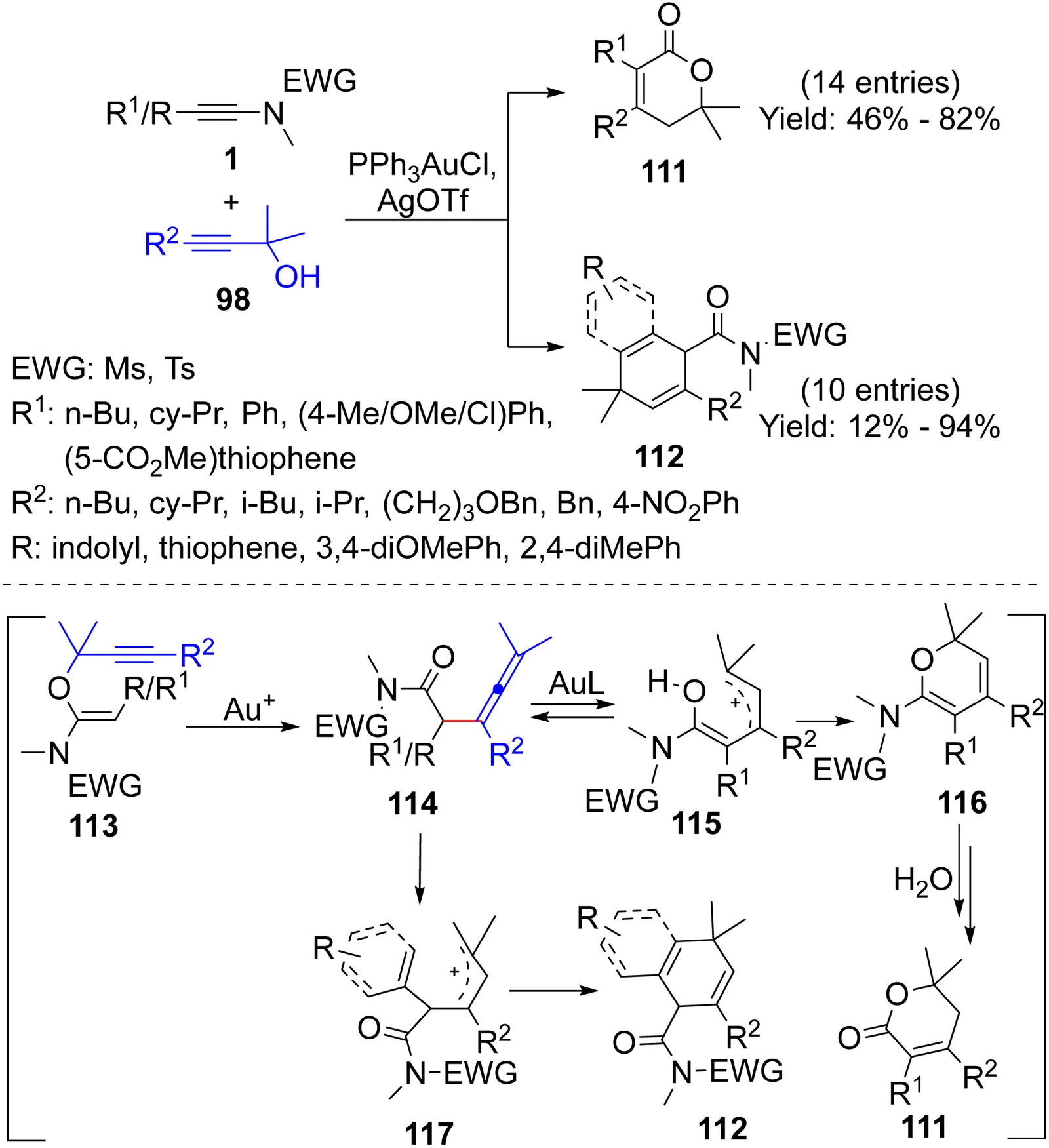
Au-catalysed one-pot annulation reaction of ynamides and propargyl alcohols.

In 2017, Liu *et al.* proposed a gold-catalysed one-pot operation of three distinct strategies for catalytic annulation using ynamide 1 and propargyl alcohol 98, resulting in carbo- and heterocycles (Scheme 37).^50^ Alkoxylation of ynamide generates propargylic enol ether intermediate 113, which undergoes a Saucy–Marbet rearrangement to produce 5-allenyl-1-amide intermediate 114. In the presence of the Au-catalyst, intermediate 114 undergoes rearrangement followed by 6-*endo*-trig cyclisation; substrates bearing R and R^1^ on the ynamide (Scheme 37) deliver products 111 and 112, respectively. The chemoselectivity of these annulations was achieved using various substituted ynamides and 1-yn-3-ols, which resulted in different cyclisations of the 5-allenyl-1-amide intermediate.

**Scheme 38:**
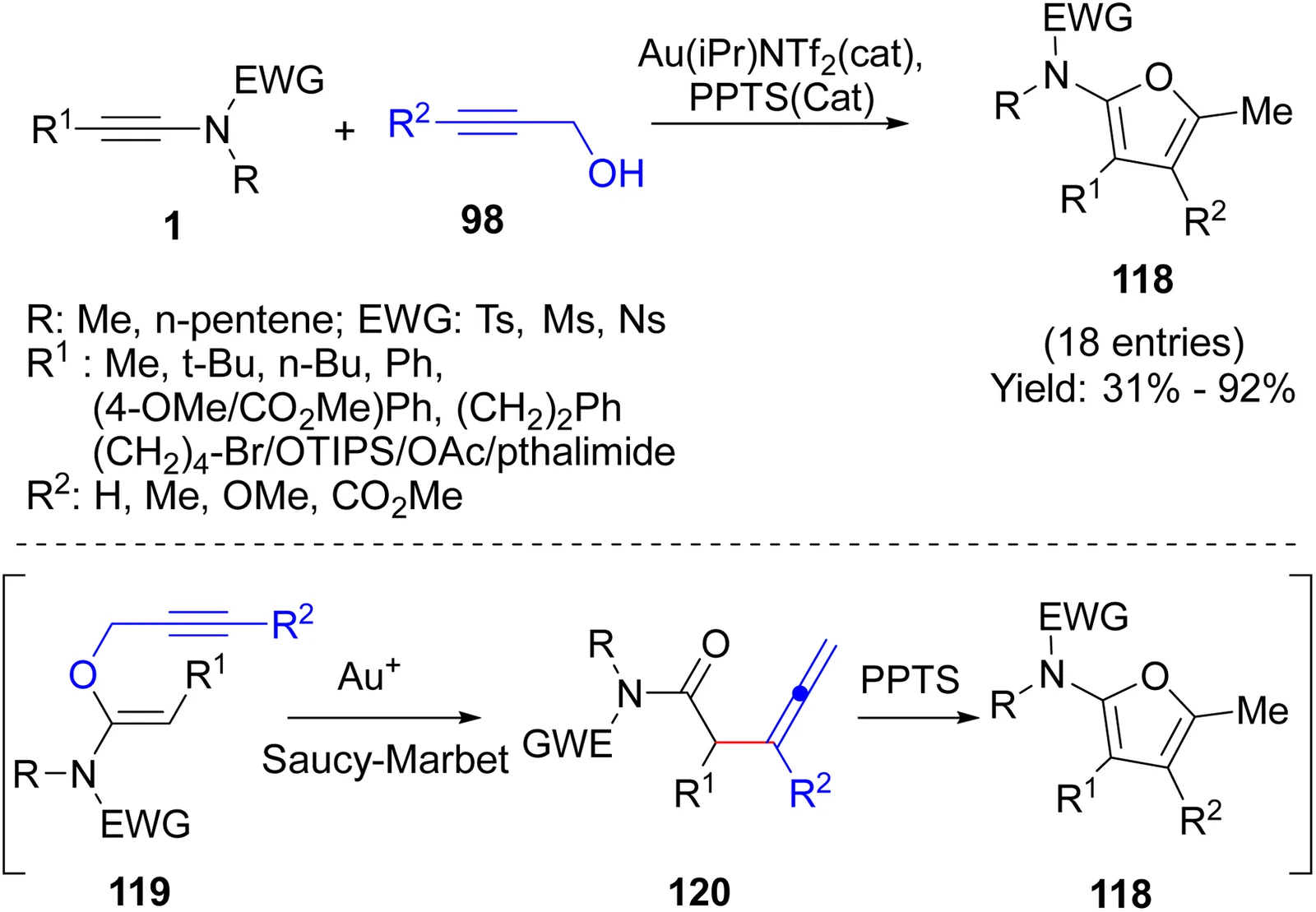
Au-catalysed cascade synthesis of 2-aminofurans from ynamides and propargyl alcohols.

Simultaneously, Sato and co-workers in 2021 demonstrated an Au(i)-catalysed one-pot synthesis of tetra-substituted 2-aminofurans by a cascade reaction between ynamides and propargylic alcohols (Scheme 38).^51^ In the presence of 1 mol% of the Au(IPr)Cl/AgNTf_2_ catalyst, the products 3,4-dienamide 120 and 2-aminofuran 118 were produced in 35% and 38% yields, respectively. The authors added PPTS to the reaction mixture in order to obtain the required 2-aminofuran with an optimal yield in a single step, which led to the complete conversion of the starting material to the cyclized product. Under ideal reaction conditions, a range of ynamides and propargylic alcohols were investigated, and the products were obtained in moderate to good yields.

The reaction proceeds through the regioselective hydroalkoxylation of ynamide by propargyl alcohol, resulting in intermediate 119, which undergoes the Saucy–Marbet rearrangement to afford 3,4-dienamide *in situ*. The enol form of this intermediate undergoes cyclisation in the presence of PPTS by nucleophilic attack of the O-atom at the carbon centre of the diene moiety and a protodeauration process.

### 2.2. [2,3]-Sigmatropic rearrangement

Over the last few decades, [2,3]-sigmatropic rearrangements have been extensively used in the formation of new C–C and C–X (X = N, O, S, and Se) bonds. Among these rearrangements, owing to the high reactivity of the ketenimine intermediate of ynamides, [2,3]-sigmatropic rearrangement involving ynamides has drawn significant attention from synthetic chemists. Herein, recent advances in this protocol in the synthesis of complex molecular architectures are reported.

**Scheme 39:**
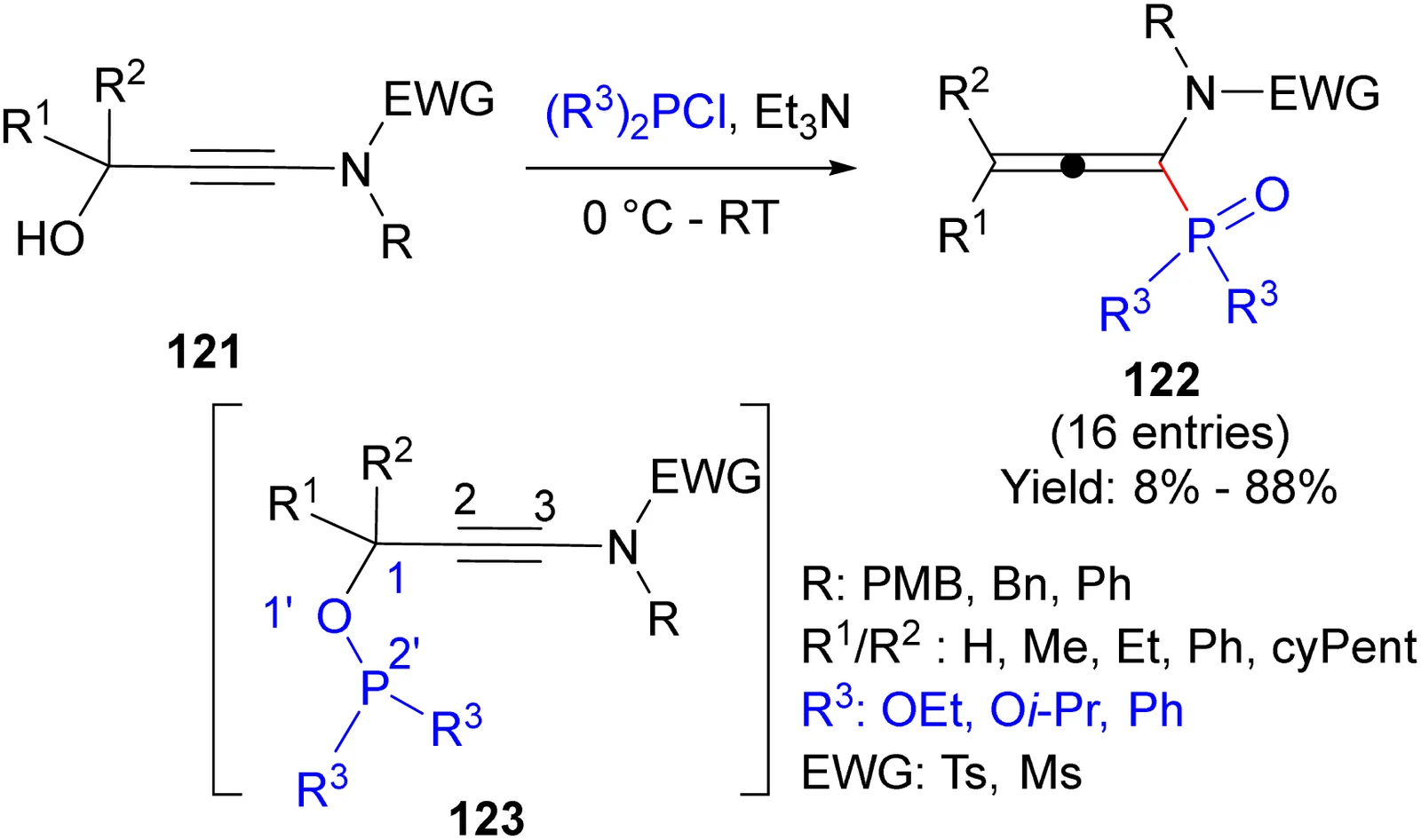
Synthesis of α-amino allenephosphonates *via* [2,3]-sigmatropic rearrangement of ynamides.

In 2012, for the first time, Rabasso and co-workers reported the synthesis of α-amino allenephosphonates *via* the [2,3]-sigmatropic rearrangement of ynamides with dialkyl chlorophosphite in the presence of triethylamine (Scheme 39).^52^ They developed a straightforward method for the synthesis of a series of α-amino allenephosphonates with varying groups on ynamides and phosphites. The electron-withdrawing, *i.e.*, tosylamine and oxazolidinone groups on the ynamides were well tolerated by this protocol, but the pyrrolidinone group failed to afford the desired product. Following the same reaction conditions, the authors also synthesised α-amino allenephosphine oxides using diphenyl chlorophosphine reagents. The reaction proceeds through the formation of intermediate 123*via* a base-catalysed coupling reaction, followed by [2,3]-sigmatropic rearrangement to give the desired α-amino allenephosphonates 122 in moderate to good isolated yields.

**Scheme 40:**
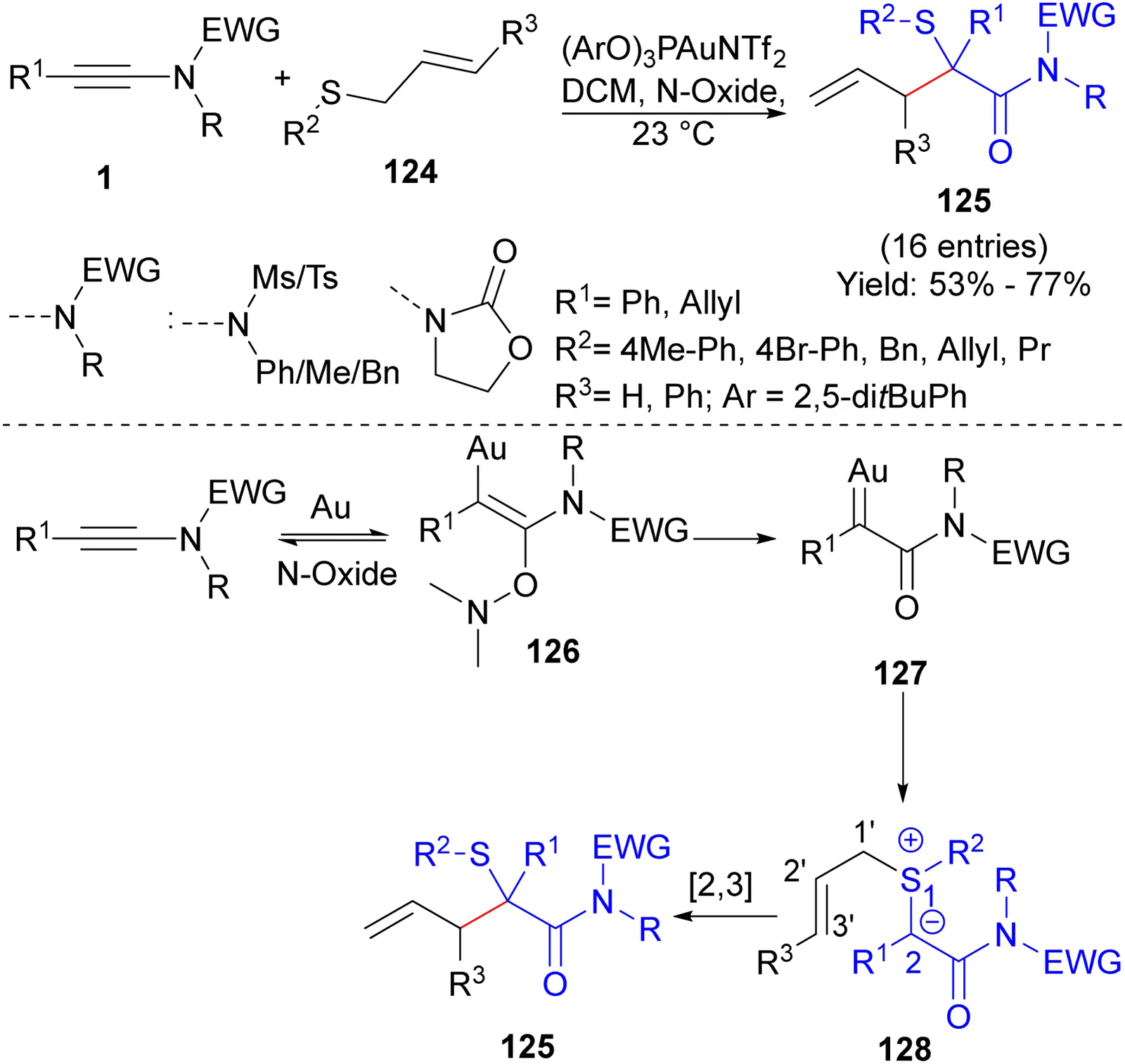
Au-catalysed [2,3]-sigmatropic rearrangement of allyl sulfonium ylides.

In 2014, Santos and Davies developed a gold-catalysed [2,3]-sigmatropic rearrangement of allyl sulfonium ylides using ynamides as the starting materials (Scheme 40).^53^ The reaction pathway involves the coordination of a gold catalyst on the triple bond of the ynamide, followed by oxidation with methylpicolinate *N*-oxide to form vinyl gold carbenoid species 126. This species resulted in allyl sulfonium ylide 128 on reaction with an allyl sulfur compound. Intermediate 128 on [2,3]-sigmatropic rearrangement gave the desired adduct 125 with a quaternary carbon centre in moderate yields. The authors also demonstrated a sole example of the formation of vicinal quaternary–tertiary carbon stereocenters with a diastereoselectivity of 10 : 1 using a substituted allyl sulfide. This method was effective for both cyclic and acyclic allyl sulfur compounds and different ynamide systems.

**Scheme 41:**
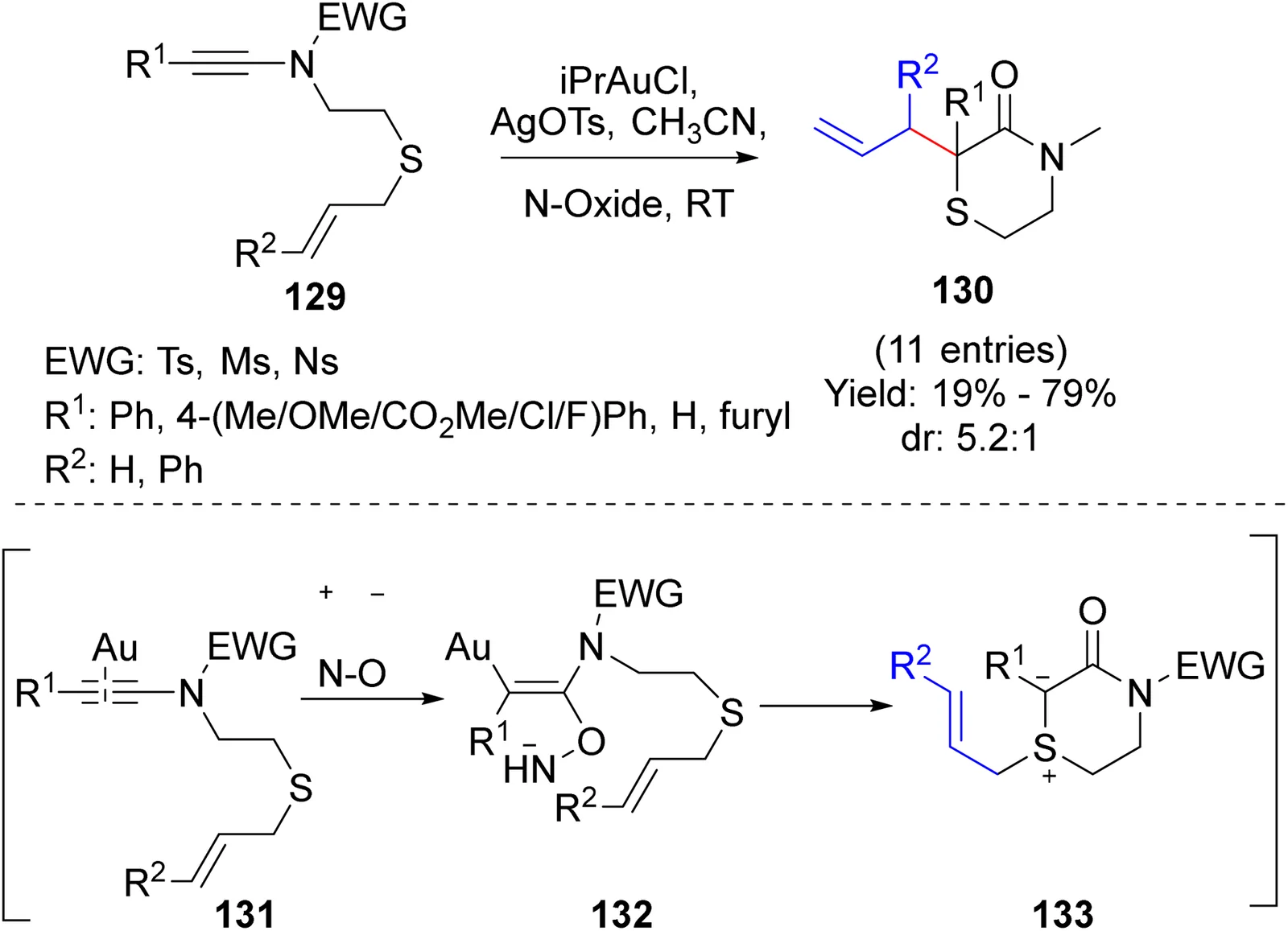
Au-catalysed cascade [2,3]-sigmatropic rearrangement of ynamides.

Inspired by the above results, in 2019, the same group reported a gold-catalysed intramolecular cascade reaction sequence to afford α-mono/α-disubstituted-α-arylthiomorpholin-3-one 130 from easily assembled ynamides 129 with tethered thioethers (Scheme 41).^54^ This protocol was conducted under mild reaction conditions with an IPrAuCl/AgOTs complex, involving oxidation, cyclisation, and then a [2,3]-sigmatropic rearrangement in a single step. This resulted in four new bonds and a sulfur-substituted quaternary centre from only one triple bond, with moderate to good yields.

The reaction tolerated various allyl units and electronically varied groups at the C-terminus of the ynamides, but trimethylsilyl-protected ynamides failed to give the preferred silylated thiomorpholin-3-one derivative. The mechanistic pathway of this reaction involves the coordination of the Au-catalyst on the triple bond 131, which undergoes intermolecular addition with the oxidant, *i.e.*, pyridine-*N*-oxide, to generate intermediate 132. This intermediate on cyclisation gives sulfonium ylide 133, which further rearranges to deliver thiomorpholin-3-one derivative 130 as the major product.

**Scheme 42:**
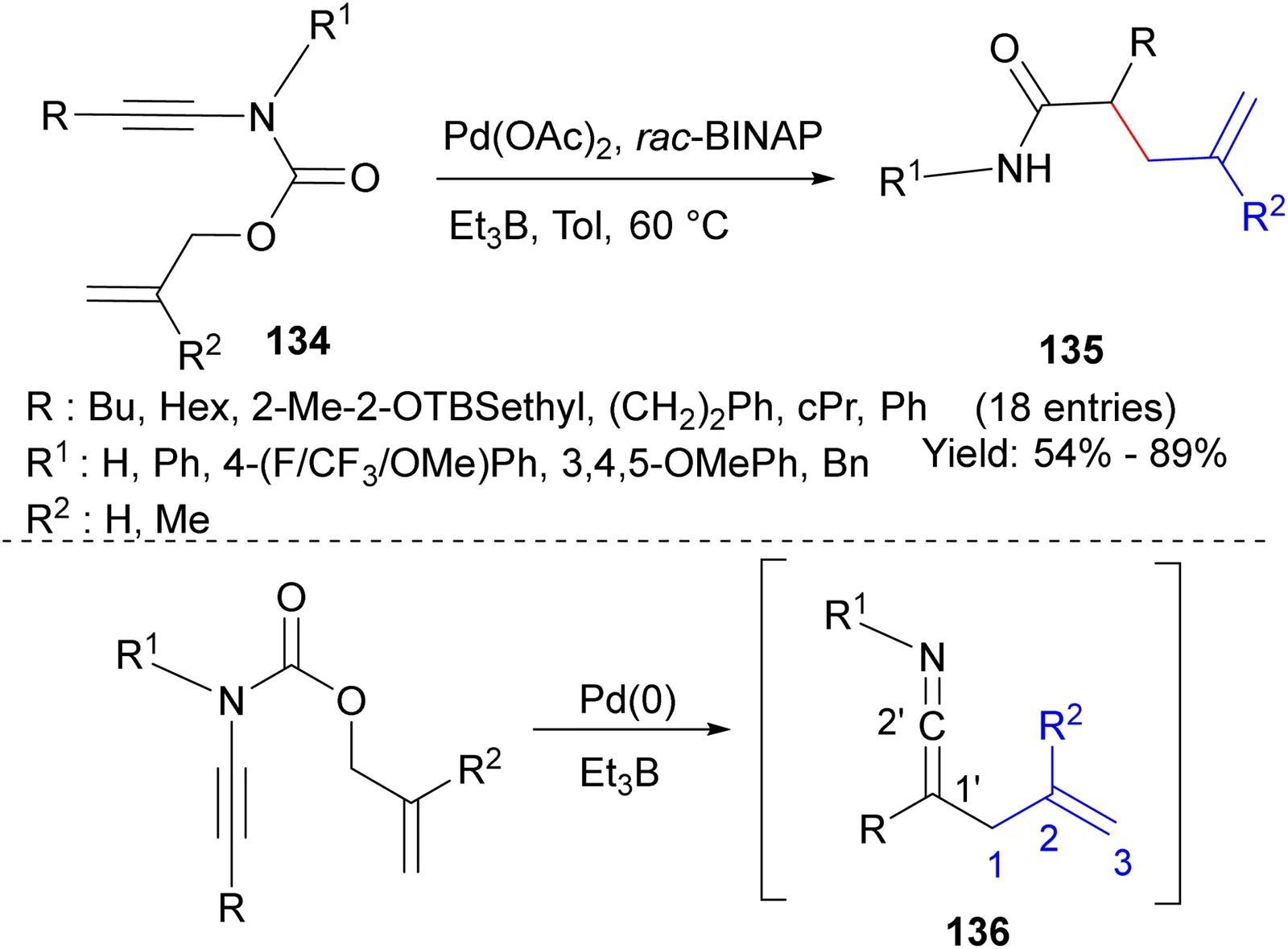
Synthesis of ketenimines *via* [2,3]-sigmatropic rearrangement.

In 2017, Alexander and Cook presented a novel method for synthesising ketenimines *via* [2,3]-sigmatropic rearrangement, which could then be transformed into α-tertiary amides 135 (Scheme 42).^55^ The reaction proceeded with the formation of ketenimine 136 through a Pd(0)-catalysed decarboxylative allylic rearrangement of *N*-alloc ynamide 134. The resulting ketenimine then undergoes a [2,3]-sigmatropic rearrangement, followed by acid hydrolysis, affording the desired product, α-tertiary amides 135, in moderate to good yields. There is only one reported example demonstrating the use of this ketenimine intermediate in the [2,3]-Wittig rearrangement process with sulfur ylide. No significant change in the reaction yield was observed when electron-donating and electron-withdrawing groups were introduced on the aryl ring of the ynamide.

**Scheme 43:**
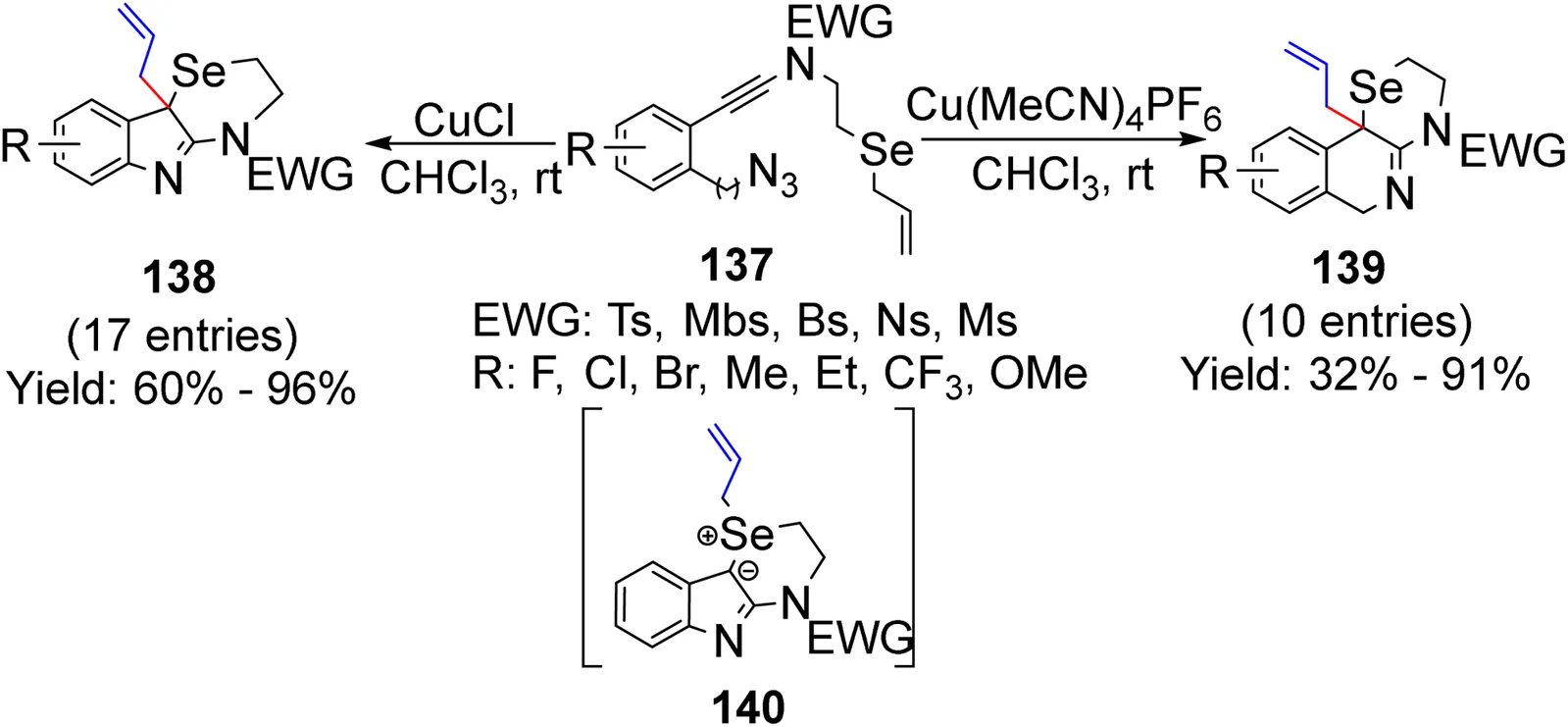
Cu(i)-catalysed [2,3]-sigmatropic rearrangement of azide-ynamides.

In 2025, Zhu and co-workers reported the first copper-catalysed [2,3]-sigmatropic rearrangement of azide-ynamides 137*via* the formation of selenium ylides (Scheme 43).^56^ The use of comparatively cheaper catalysts and easily synthesized ynamides made this reaction more feasible than the above gold-catalysed reactions. The reaction provided access to [1,4]selenazino[3,2-*b*]indoles 138 and [1,4]selenazino[3,2-*b*]isoquinolines 139 bearing new C–C and C–Se bonds, along with a quaternary carbon stereocenter. A range of substrates have been employed smoothly under mild reaction conditions to deliver the desired products in good to excellent yields. The group also performed a catalytic asymmetric reaction in the presence of the (*S*)-DTBM-GARPHOS chiral bisphosphine ligand to afford the desired product with a chiral centre in low yield and enantioselectivity. The reaction proceeds through a sequence of steps: coordination of the copper catalyst to the triple bond of allyl selenide-linked azide-ynamide, intramolecular nucleophilic attack by the azide group, extrusion of N_2_ for the formation of α-imino copper carbene, and trapping by the selenium atom to give cyclized intermediate 140. Thereafter, selenium ylide 140 undergoes a [2,3]-sigmatropic rearrangement to deliver the final product.

### 2.3. Miscellaneous

**Scheme 44:**
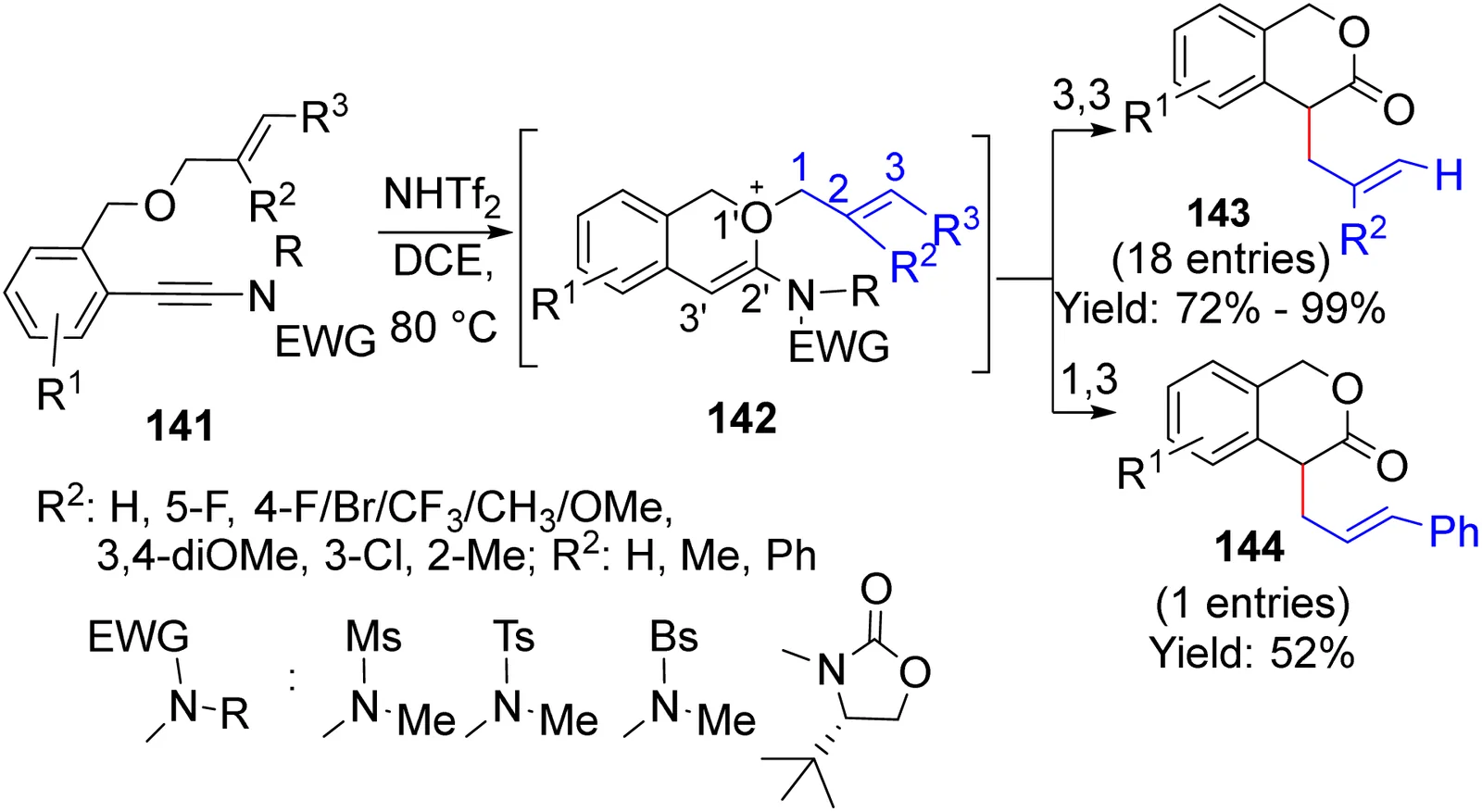
Metal-free synthesis of 3-isochromanones *via* intramolecular alkoxylation–cyclisation of ynamides.

In continuation of their previous studies on ynamide reactivity,^13,14^ in 2019, Ye and co-workers reported a metal-free synthesis of 3-isochromanones *via* an intramolecular alkoxylation-initiated cyclisation of an allyl ether-containing ynamide 141 (Scheme 44).^57^ This methodology resulted in the formation of highly functionalised 3-isochromanones in good to excellent yields *via* an atom-economic, mild reaction pathway. Initially, oxonium intermediate 142 is formed by the attack of the alkoxyl moiety on the acid-activated ynamide system; this intermediate undergoes a subsequent [3,3]-sigmatropic rearrangement and is trapped with a trace amount of water present in the reaction, forming target molecule 143. Chiral ynamides underwent stereocontrolled [3,3]-sigmatropic rearrangement to afford enantioenriched 3-isochromanones in excellent yields. However, when the ynamide with a phenyl substitution on the allyl ether moiety was subjected to the same reaction conditions, the reaction proceeded *via* tandem alkoxylation/[1,3]-sigmatropic rearrangement, furnishing the desired product 144. This occurred due to the cation-stabilising ability or steric hindrance created by the phenyl group, which can ease the [1,3]-sigmatropic rearrangement through dissociation of the stable allyl cation.

**Scheme 45:**
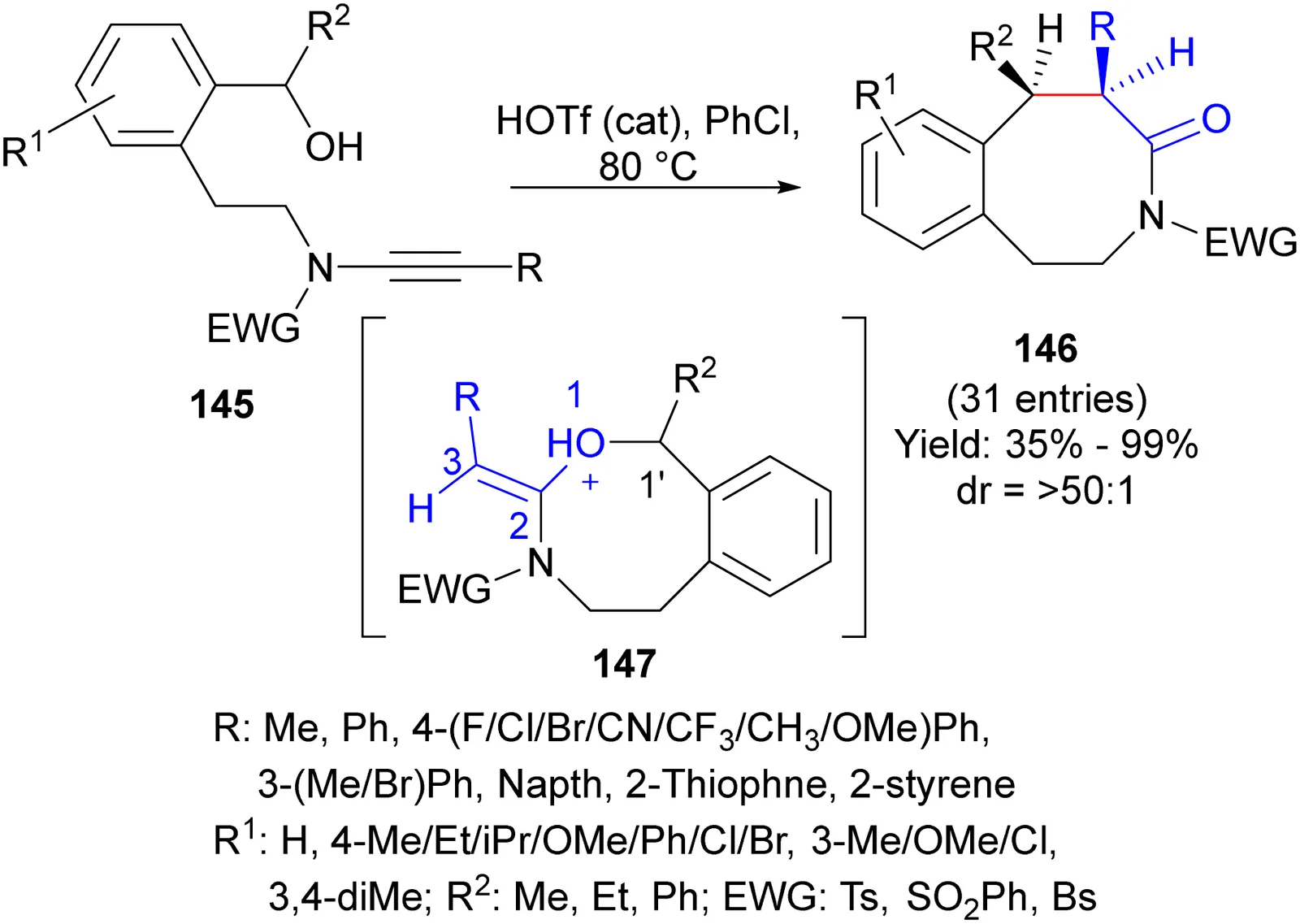
[1,3]-Sigmatropic rearrangement of ynamides.

After exploring the ynamide reactivity in the field of [3,3]-sigmatropic rearrangement, the same group in 2019 demonstrated [1,3]-sigmatropic rearrangement of ynamides (Scheme 45) in a stereospecific way.^58^ They proposed atom-economical and metal-free cascade intramolecular hydroalkoxylation, followed by a stereospecific [1,3]-sigmatropic rearrangement process to access the synthesis of medium-sized lactams in the presence of a Brønsted acid catalyst. This novel method provided outstanding diastereoselectivity and a wide variety of substrate scope. Furthermore, the asymmetric version of this methodology was developed using a chiral spirophosphoramide catalyst, which resulted in greater enantiomeric ratios. The reaction starts with the attack of the hydroxyl group on the TfOH-activated ynamide to give the vinyl oxonium ether intermediate 147 by regeneration of the acid catalyst. This vinyl ether intermediate 147 on subsequent [1,3]-sigmatropic rearrangement afforded eight-membered lactams 146 in good yield.

## 3 Conclusion

The aforementioned synthetic methods clearly demonstrate the importance of ynamides as versatile structural motifs in stereoselective sigmatropic rearrangements. Transition metal- and Lewis acid-catalysed stereoselective sigmatropic rearrangements of ynamides have made substantial progress in the past decades. The discussion presented in the preceding sections highlighted several noteworthy contributions in this field, categorised by [3,3]- and [2,3]-sigmatropic rearrangements. In this review, effective techniques featuring a wide substrate scope, good regio- and stereoselectivity, and a variety of tandem processes, as well as new applications in target-oriented synthesis, are comprehensively discussed. Numerous elegant intramolecular approaches have been proposed that employ allyl-substituted ynamides to enable the rapid assembly of a wide range of functionalised heterocycles in a concise and flexible manner. In some cases, a quaternary centre in a cyclic framework is generated with good diastereoselectivity. In contrast, the generation of quaternary stereocenters in acyclic frameworks has been reported in a few publications, highlighting the difficulty of preparing such centres in acyclic molecules.

## Conflicts of interest

There are no conflicts to declare.

## Data Availability

No primary research results, software or code have been included and no new data were generated or analysed as part of this review.
